# Algal methylated compounds shorten the lag phase of *Phaeobacter inhibens* bacteria

**DOI:** 10.1038/s41564-024-01742-6

**Published:** 2024-07-05

**Authors:** Martin Sperfeld, Delia A. Narváez-Barragán, Sergey Malitsky, Veronica Frydman, Lilach Yuda, Jorge Rocha, Einat Segev

**Affiliations:** 1 Department of Plant and Environmental Sciences, Weizmann Institute of Science, Rehovot, Israel; 2 Department of Life Sciences Core Facilities, Weizmann Institute of Science, Rehovot, Israel; 3 Department of Chemical Research Support, Weizmann Institute of Science, Rehovot, Israel; 4 Agricultura en Zonas Áridas, Centro de Investigaciones Biológicas del Noroeste, La Paz, Mexico; 5 Present Address: Institute of Microbiology, ETH, Zurich, Switzerland

**Keywords:** Water microbiology, Bacteriology

## Abstract

The lag phase is key in resuming bacterial growth, but it remains underexplored particularly in environmental bacteria. Here we use transcriptomics and ^13^C-labelled metabolomics to show that the lag phase of the model marine bacterium *Phaeobacter inhibens* is shortened by methylated compounds produced by the microalgal partner, *Emiliania huxleyi*. Methylated compounds are abundantly produced and released by microalgae, and we show that their methyl groups can be collected by bacteria and assimilated through the methionine cycle. Our findings underscore the significance of methyl groups as a limiting factor during the lag phase and highlight the adjustability of this growth phase. In addition, we show that methylated compounds, typical of photosynthetic organisms, prompt diverse reductions in lag times in bacteria associated with algae and plants, potentially favouring early growth in some bacteria. These findings suggest ways to accelerate bacterial growth and underscore the significance of studying bacteria within an environmental context.

## Abstract (web summary)

Algal-associated bacteria assimilate methyl groups through the methionine cycle as ready-made carbon building blocks that jump-start growth.

## Main

Marine heterotrophic bacteria rely on organic carbon synthesized by photosynthetic microorganisms and consume approximately half of the carbon fixed by these primary producers^1–3^. The primary producers, namely microalgae, produce organic carbon by fixing CO_2_ in a process that is subjected to temporal dynamics^4,5^. Photosynthetic productivity is poor at night and in winter, when light intensities decline or nutrients are depleted. Consequently, heterotrophic bacteria endure prolonged phases of starvation^6^. With the onset of microalgal productivity, bacteria must rapidly activate their metabolism to outgrow co-occurring competitors^7,8^.

The transition from starvation and preparation for cell division occur during the lag phase^9^. Recent studies highlight that bacteria undergo a global metabolic reorganization during the lag phase^10^ and synthesize essential building blocks (such as nucleotides and amino acids), which are then incorporated into larger macromolecules including DNA, RNA and proteins^11–13^. Some building blocks can be acquired from external sources and alleviate metabolic needs during the lag phase^13^. While the lag period has been well studied in human pathogens and food-spoiling bacteria^10^, much is unknown about the lag phase in the marine environment.

Several groups of marine heterotrophic bacteria, such as the common *Roseobacter* group^14^, often associate with microalgae^1^. Much knowledge exists regarding the metabolic pathways through which bacteria use algal metabolites for growth^15,16^. However, a substantial knowledge gap remains regarding the early physiological response of bacteria upon encountering algae after a period of starvation. As bacteria often endure periods of starvation in nature^17^, strategies to optimize the initial interaction with algal hosts could prove beneficial for the ecological success of marine bacteria. In this study, we sought to understand the post-starvation response of a model *Roseobacter* species, *Phaeobacter inhibens*, to microalgal metabolites and to determine the underlying mechanisms.

We analysed the metabolism of *P. inhibens* while it grows with *Emiliania huxleyi* microalgae (also known as *Gephyrocapsa huxleyi*^18^) or is supplemented with selected microalgal metabolites. We discovered that methyl groups represent a metabolic ‘bottleneck’ to bacterial growth even when ample carbon and nutrients are made available. Nano- to micromolar concentrations of methylated compounds, which are abundantly produced by microalgae^19,20^, shorten the bacterial lag phase without affecting the growth rate. To understand the mechanisms underlying this lag phase shortening, we analysed bacterial transcriptomes, estimated cellular methyl group requirements and examined the incorporation of ^13^C-labelled methyl groups. Our results suggest that during the bacterial lag phase, assimilatory methyl group requirements can be alleviated by abundant algal metabolites, thereby shortening the lag duration.

## Results

### Algae impact bacterial gene expression of methyl metabolism

We first investigated the metabolic response of *P. inhibens* bacteria during co-cultivation with *E. huxleyi* algae. This algal–bacterial pair naturally co-occurs in the environment and was previously studied by us^21,22^ and by others^23–25^. In co-cultures, bacteria rely entirely on algal-secreted metabolites for growth^21^. As a reference condition, we analysed bacterial pure cultures that received glucose as a sole carbon source (Supplementary Figs. 1–3, Supplementary Tables 1 and 2, and Supplementary Data 1). Comparing pure bacterial cultures in nutrient-rich conditions with glucose as the sole carbon source with bacteria in co-cultures receiving a continuous but limited supply of algal metabolites has limitations. However, this comparison can reveal important differences and generate hypotheses for further exploration.

**Fig. 1:**
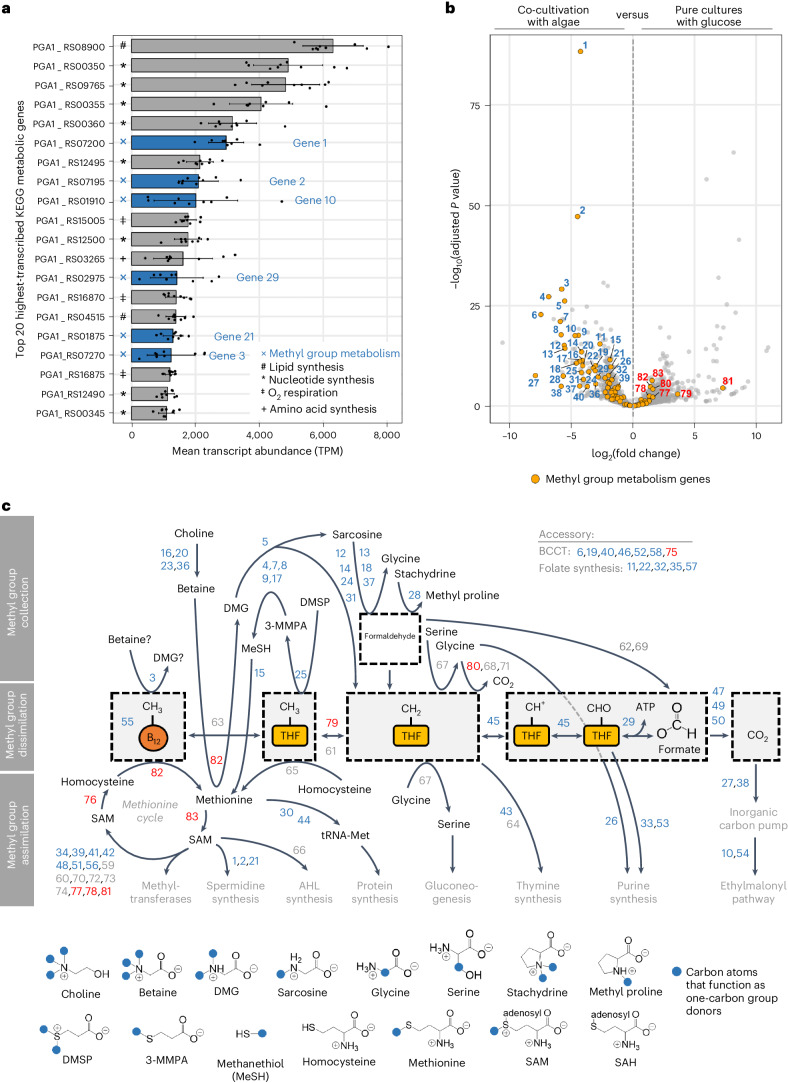
Identification of metabolic genes that are transcribed in *P. inhibens* bacteria during co-cultivation with *E. huxleyi* microalgae. **a**, Co-culture RNA-sequencing revealed that bacterial genes involved in methyl group metabolism were among the highest-transcribed KEGG metabolic genes in the presence of algae (blue bars). The bars represent the mean of nine algal–bacterial co-cultivation samples taken from three different time points (see Supplementary Fig. 1 for the sampling points). The error bars depict standard deviations. Labels on the *y*-axis are RefSeq accession numbers. KEGG annotations of respective methyl group metabolism genes: *S*-adenosylmethionine decarboxylase (gene 1), trimethylamine—corrinoid protein Co-methyltransferase (gene 3), spermidine synthase (gene 2), crotonyl-CoA carboxylase/reductase (gene 10), THF ligase (gene 29) and ornithine decarboxylase (gene 21). See Supplementary Data 1 for transcript abundances per sample. **b**, Methyl group metabolism genes were upregulated in bacteria that were co-cultivated with algae (left; blue numbers) compared with bacteria in pure cultures that grew exponentially with glucose (right; red numbers). Samples used for differential gene expression analysis are presented in Supplementary Fig. 1. **c**, Bacterial methyl group metabolism genes include reactions involved in collecting, dissimilating and assimilating methyl groups from methylated donor compounds. Included are genes that either are directly involved in transforming methyl groups or are accessory and encode transporters or THF synthesis. The blue and red numbers indicate up- and downregulated genes, respectively (thresholds for colouring: adjusted *P* < 0.05; log_2_(fold change) < −0.585 and > +0.585). Gene numbers are identical throughout the text and figures, with accession numbers and functional annotations listed in Supplementary Table 3. The blue dots in the chemical structures represent carbon atoms (mainly methyl groups) that function as one-carbon group donors. BCCT, betaine–carnitine–choline transporter family; DMG, dimethylglycine; DMSP, dimethylsulfoniopropionate; 3-MMPA, 3-*S*-methylmercaptopropionate; MeSH, methanethiol; SAM, *S*-adenosylmethionine; SAH, *S*-adenosylhomocysteine.

Based on KEGG metabolic pathway definitions, we identified catabolic and anabolic processes that are highly expressed in bacteria during co-cultivation with algae. We found that genes involved in using methylated compounds were among the 20 highest-transcribed bacterial metabolic genes (Fig. 1a and Supplementary Data 1). Examples include a gene encoding a cobalamin-dependent methyltransferase (gene 3 in Fig. 1a), a gene encoding a formate–tetrahydrofolate (THF) ligase (gene 29) and genes involved in spermidine synthesis (genes 1, 2 and 21). Methyl group metabolism emerged as a central process during bacterial growth with algae. Consequently, to identify relevant genes that might have escaped annotation in the KEGG database, we conducted a manual search for bacterial genes involved in metabolizing methylated compounds, culminating in a comprehensive list of 83 genes (Supplementary Table 3 and Supplementary Fig. 4). Differential gene expression (DE) analysis indicated that the identified genes were markedly upregulated in the presence of algae, compared with bacterial pure cultures grown with glucose (see DE results of all genes in Supplementary Data 1 and Fig. 1b). We depicted bacterial utilization of methyl groups during interactions with algae by overlaying methyl group metabolism reactions with our DE results (Fig. 1c). This overview underscores that bacteria collect, dissimilate and assimilate methyl groups during interactions with algae, suggesting that methyl groups are an important resource for algal-associated bacteria.

### Lag phase shortening is triggered by DMSP

**Fig. 2:**
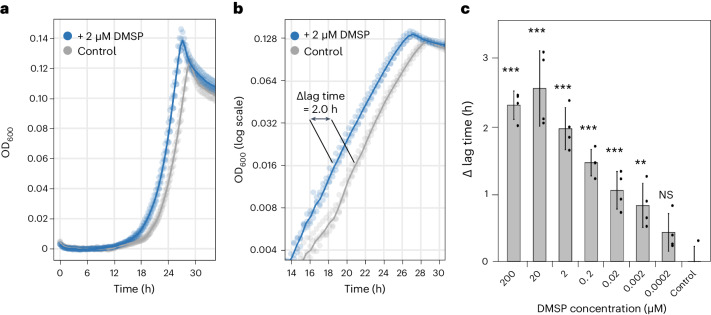
The methylated compound DMSP induces lag phase shortening in *P. inhibens* bacteria. **a**, Micromolar concentrations of DMSP stimulated earlier growth of bacteria (blue) compared with control cultures supplemented with water (grey). **b**, Semi-log_2_-transformed growth curves show that doubling times were unchanged, as evident from the identical slopes, while lag times were shortened. Lag phase shortening is expressed as the difference between mean lag times measured for supplemented cultures compared with control cultures (Δlag time; Methods). **c**, Exposure to nanomolar concentrations of DMSP resulted in significant bacterial lag phase shortening. All growth experiments were conducted in artificial seawater medium (ASW_b_) with 1 mM glucose as carbon source, using glucose-grown, stationary phase pre-culture bacteria as inoculum. For each condition, four biological replicates were used (four wells of a 96-well plate were inoculated with the same pre-culture). In **a** and **b**, dots represent individual measurements for each of the four replicates per time point, and lines depict a smoothed average. Error bars in **c** indicate the standard deviation of the estimated difference between means, with dots representing biological replicates. One-way ANOVA and Dunnett’s test were used to identify significant differences between control and supplemented cultures (adjusted *P* value thresholds: ***P ≤ 0.001; **P ≤ 0.01; not significant (NS) > 0.05). Source data

To investigate the influence of methylated compounds on bacteria, we conducted a series of growth experiments using the methylated compound dimethylsulfoniopropionate (DMSP) as a model substrate. DMSP is naturally produced by microalgae and was extensively studied in the context of bacterial metabolism and growth^26,27^. In our experiments, bacterial pure cultures were pre-cultivated with glucose as a sole carbon source until reaching the stationary phase. Then, stationary phase bacteria were used to initiate fresh cultures that contained the same glucose medium, but that were additionally supplemented with DMSP (or with water as control). We found that micromolar concentrations of DMSP stimulated the growth of bacteria (Fig. 2a). Plotting the growth curves semilogarithmically revealed that the low levels of added DMSP affected specifically the bacterial lag period; lag times were shortened while growth rates were not affected (Fig. 2b and Supplementary Fig. 5a). We further quantified the lag phase shortening effect by calculating the Δlag time, which is the time difference between the onset of growth in control cultures and that of supplemented cultures (Fig. 2b and Supplementary Fig. 5b). By analysing Δlag times under different DMSP concentrations, we found that lag times were shortened by up to 2.5 h and that nanomolar concentrations of DMSP already resulted in significantly lower lag times (Fig. 2c and Supplementary Fig. 5c). Interestingly, concentrations above 20 μM did not increase the delta lag phase. This suggests that there may be a threshold at which the demethylation machinery becomes saturated, leading to diminished sensitivity to DMSP, as previously suggested^28^. Our results show that in the presence of a utilizable carbon source such as glucose (provided at a concentration of 1 mM in our experiments), exposure to low levels of the methylated compound DMSP leads to the shortening of the bacterial lag phase.

### Methyl groups are involved in lag phase shortening

**Fig. 3:**
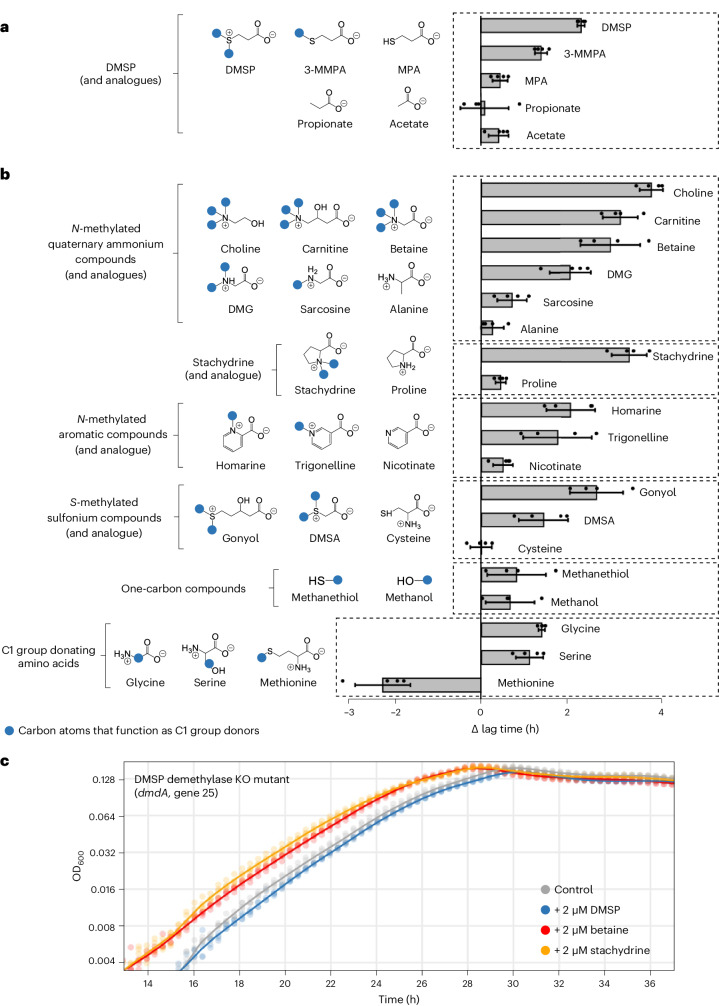
Methyl groups and other one-carbon moieties induce lag phase shortening in *P. inhibens* bacteria. **a**, Growth experiments with analogues of DMSP (2 μM) revealed that methyl groups are required for lag phase shortening. The number of methyl groups per molecule correlated with the extent of lag phase shortening. **b**, Growth experiments further revealed that multiple methylated compounds (2 μM) trigger lag phase shortening, while non-methylated analogues at the same concentration were less or not effective. In **a** and **b**, the blue dots in chemical structures represent carbon atoms (mainly methyl groups) that function as one-carbon group donors. The bars represent differences between mean lag times measured for supplemented cultures compared with control cultures (Δlag time). Each condition was analysed with four biological replicates, represented by dots, and error bars indicate the standard deviation of the difference between estimated means. **c**, Lag phase shortening was no longer evident in a *P. inhibens dmdA* knock-out mutant upon exposure to DMSP, while other methylated compounds (betaine and stachydrine) still triggered lag phase shortening. The dots represent individual measurements for four biological replicates, and the lines depict a smoothed average. Source data

To determine whether the methyl groups of DMSP are involved in lag phase shortening, we used analogues of DMSP that harbour different numbers of methyl groups (Fig. 3a and Supplementary Figs. 6 and 7). We found that the extent of lag phase shortening correlated with the number of methyl groups per molecule, while non-methylated analogues did not have a measurable impact on the lag period (see Supplementary Table 4 for statistical significance and Fig. 3a). This finding suggests that the methyl groups of DMSP drive lag phase shortening.

To substantiate the role of methyl groups, we investigated whether other methylated compounds that are commonly produced by microalgae induce lag phase shortening (Fig. 3b). We selected methylated compounds with different chemical properties including *N*-methylated, *S*-methylated, small one-carbon compounds and amino acids (which are themselves not methylated but can donate one-carbon groups to the cellular methyl group metabolism). All compounds were added at a concentration of 2 μM. The results show that supplementation with all tested methylated compounds resulted in shortening of the lag phase (see Supplementary Table 4 for statistical significance and Fig. 3b), while the doubling times were largely unchanged (Supplementary Figs. 6 and 7). The extent of lag phase shortening correlated with the number of methyl groups per molecule, in line with our results with the DMSP analogues (Fig. 3a). Of note, methionine was an exception and prolonged the lag phase. Growth inhibitions by methionine were reported for other bacteria and may result from feedback loops that regulate intracellular methionine concentrations^29^. Interestingly, we observed a more pronounced difference in the reduction of the lag phase due to altered amounts of methyl groups on DMSP (Fig. 3a) compared with changes in the concentration of the added DMSP (Fig. 2c). While the molecular mechanisms underlying this difference are not fully understood, a possible explanation previously suggested is the insensitivity of DMSP catabolic enzymes to increasing levels of DMSP^28^ and the involved transporters that may become saturated at higher concentrations^30^. In summary, our findings show that different methylated compounds prompt a reduction in the bacterial lag phase and suggest a correlation between the quantity of methyl groups present in a compound and the degree of lag reduction.

### Lag phase shortening requires methyl group collection

The involvement of methyl groups in lag phase shortening could be explained by three possible scenarios: (1) methyl groups are a limiting resource, (2) methylated compounds are sensed by receptor proteins that trigger a signal cascade and (3) methylated compounds act as intracellular solutes with protective functions. To distinguish between these possibilities, we generated a *P. inhibens* knock-out (KO) mutant that is unable to collect methyl groups from a respective donor compound. We deleted the *dmdA* gene (Fig. 1c; gene 25), which codes for the well-characterized DMSP demethylase^31^. The deletion of the demethylase gene is expected to hamper the collection of methyl groups from DMSP, while it is not expected to affect extracellular receptor binding or internalization of the compound. Our data show that DMSP-dependent lag phase shortening was abolished in the *dmdA* KO mutant (Fig. 3c). Importantly, the mutant still showed lag phase shortening when supplemented with other methylated compounds (Fig. 3c). Our results suggest that methyl groups are a limiting resource during the lag phase and that bacteria can overcome this bottleneck by collecting methyl groups from external compounds.

### Calculations highlight assimilatory methyl group needs

Methyl groups may function as a limiting resource either by being assimilated as one-carbon (C1) group substrates or by being dissimilated for ATP generation (Fig. 1c). To explore the possible fate of methyl groups during the lag phase, we estimated the assimilatory and dissimilatory C1 requirements needed for one cell duplication. Based on numbers determined for exponentially growing *Escherichia coli*^32^ and according to the metabolic model of *P. inhibens*^33^, we calculated that a single *P. inhibens* cell requires 190.1 amol of C1 groups to synthesize the building blocks needed for one cell duplication, and needs 23,800 amol ATP (see Supplementary Table 5 and Methods for further details and explanations), with one ATP generated per methyl group (Fig. 1c; gene 29). Under our experimental conditions, a freshly initiated *P. inhibens* culture at a density of 25,000 colony-forming units (CFUs) ml^−1^ already exhibited considerable lag phase reduction when supplemented with 2 nM DMSP (Fig. 2c). This DMSP concentration can fulfill almost 84% of the assimilatory C1 requirements while only 0.7% of the dissimilatory needs. Despite potential biases in our estimations, this calculation highlights the considerable potential of methyl groups to alleviate assimilatory needs and accelerate the lag phase, rather than substantially affecting the high demand of dissimilatory processes.

### Upregulation of methyl assimilation genes during lag phase

To explore the fate of methyl groups within cells, we analysed the bacterial transcriptome in response to DMSP, focusing on the lag phase. If methyl groups are assimilated during this phase, we expect transcriptional regulation of associated assimilation functions. The transcriptome of *P. inhibens* was previously shown to be remarkably dynamic, revealing rapid responses occurring within minutes following treatment^34^. Therefore, RNA was sampled 15 min and 40 min after initiation of fresh cultures, using glucose-grown stationary phase bacteria as inoculum. The cultures either contained glucose as sole carbon source or were supplemented with DMSP (Supplementary Figs. 8–10, Supplementary Tables 6 and 7, and Supplementary Data 2). Our data show that the addition of DMSP resulted in the upregulation of 43 methyl group metabolism genes during the lag phase and the downregulation of 12 genes (Supplementary Figs. 11–13), an effect that was most pronounced 15 min after the initiation of fresh cultures.

**Fig. 4:**
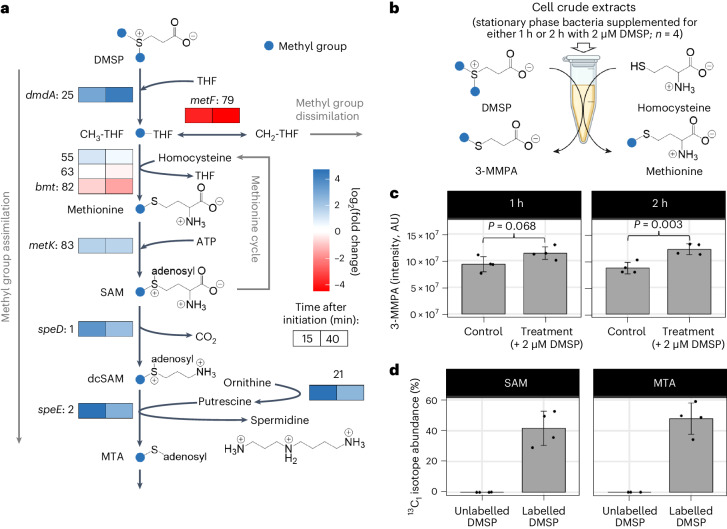
Transcriptional, metabolic and enzymatic response of *P. inhibens* bacteria towards DMSP during the lag phase. **a**, The transcriptional response was analysed in freshly initiated bacterial cultures that were supplemented with 50 µM DMSP, compared with control cultures without DMSP (all cultures contained 1 mM glucose; Supplementary Fig. 8). Gene expression changes (log_2_ fold change) are indicated as coloured boxes that correspond to the age of the culture after initiation—15 min (left box) and 40 min (right box). Colours show upregulation (blue) and downregulation (red) in response to DMSP. Numbers to the left of the boxes or above are gene numbers (see Supplementary Table 3 for accession numbers and functional annotations). RNA-sequencing was conducted using three biological replicates per condition. **b**, Evaluating methionine synthesis activity in response to DMSP. Stationary phase bacteria were supplemented with either 2 µM DMSP or water as control (four biological replicates per condition) and sampled for cell disruption 1 h and 2 h after supplementation. Resulting cell crude extracts were used for in vitro enzymatic reactions (volume = 1.5 ml; 0.35 mg protein per reaction) with added homocysteine (2 mM) and DMSP (200 µM) to catalyse the formation of 3-MMPA, which is the demethylated form of DMSP. Product formation was measured by LC–MS analysis. **c**, Cell crude extracts of DMSP-supplemented stationary phase bacteria produced slightly more 3-MMPA after 1 h (*P* = 0.068), and significantly more 3-MMPA after 2 h (*P* = 0.003), compared with the non-supplemented control cultures. The bars represent the means of four biological replicates (dots), with error bars showing the standard deviation. Two-tailed Student’s *t*-test was used to calculate *P* values. **d**, Tracking the metabolic fate of DMSP methyl groups during the lag phase. Freshly initiated bacterial cultures were supplemented with 50 µM [^13^C-methyl]DMSP, in which both methyl groups are ^13^C-labelled. The intracellular formation of ^13^C-labelled metabolites was monitored 2 h after initiation by LC–MS analysis—a time point at which higher DMSP demethylation activity was observed (shown in **c**). The method identified ^13^C-labelled SAM (isotope abundance for M + 1: 41.6%) and ^13^C-labelled MTA (isotope abundance for M + 1: 47.9%).

Specifically, genes involved in DMSP catabolism were upregulated (Supplementary Fig. 11; genes 4, 7, 8, 9, 17 and 25), including the DMSP demethylase gene (*dmdA*: gene 25). The most upregulated genes were involved in synthesizing spermidine (Supplementary Fig. 11; genes 1, 2 and 21), which is a metabolite associated with cell growth^35^. The most downregulated gene was involved in methyl group oxidation—a regulation that may impede methyl group dissimilation (Supplementary Fig. 11; gene 79). Interestingly, DMSP demethylation (*dmdA*: gene 25), spermidine synthesis (*speD*: gene 1, *speE*: gene 2, and gene 21) and methyl group oxidation (*metF*: gene 79) are all linked via the methionine cycle (Fig. 4a). This link is further supported by the upregulation of *metK* (gene 83), a gene that is involved in converting methionine into *S*-adenosylmethionine (SAM; Fig. 4a). In summary, transcriptome analyses suggest that DMSP methyl groups are assimilated during the bacterial lag phase through the methionine cycle.

### DMSP methyl groups are assimilated via the methionine cycle

To verify the transcriptomics results suggesting the involvement of the methionine cycle in processing collected methyl groups, we sought direct experimental validation. First, we examined whether bacteria in the stationary phase, before transitioning to the lag phase, possess the necessary enzymatic machinery to demethylate DMSP and generate methyl groups for use in the methionine cycle. We therefore generated crude cell extracts from stationary phase bacteria supplemented with DMSP (or water as control) for 1 h or 2 h. These extracts were then incubated with the substrates required for DMSP demethylation and the utilization of methyl groups in the methionine cycle—homocysteine and DMSP (Fig. 4b). The formation of the demethylated product of DMSP, 3-*S*-methylmercaptopropionate (3-MMPA), was measured using Liquid chromatography–mass spectrometry (LC–MS) analysis (Fig. 4c). Our results indicate that crude extracts from DMSP-supplemented stationary cells produced more 3-MMPA, with higher levels observed after a longer incubation period with DMSP (2 h compared with 1 h). This suggests that exposure to DMSP during the stationary phase influenced the enzymatic demethylation machinery that was subsequently activated in the crude extract (Fig. 4c). Importantly, analysis of the stationary bacteria used to prepare the crude extract showed significantly lower levels of 3-MMPA, regardless of DMSP supplementation (Supplementary Fig. 14). These findings confirm that stationary *P. inhibens* bacteria possess the enzymatic machinery necessary for DMSP demethylation.

To trace the fate of collected methyl groups, we supplemented bacteria with [^13^C-methyl]DMSP, where both methyl group carbons were labelled. Using glucose-grown stationary phase bacteria, we initiated fresh cultures with either 50 µM [^13^C-methyl]DMSP or 50 µM unlabelled DMSP, with 1 mM glucose as a sole carbon source. After 2 h (Supplementary Fig. 8)—a time point after which DMSP demethylation activity was higher (Fig. 4c)—cells were collected and subjected to metabolite extraction and LC–MS analysis. Our findings reveal that DMSP methyl groups contribute to SAM and *S*-methyl-5′-thioadenosine (MTA) synthesis with isotope abundances of 41.6% and 47.9%, respectively (values given for M + 1 isotopologues with a single ^13^C label; Fig. 4d, Supplementary Fig. 15 and Supplementary Data 4). SAM is formed via the methionine cycle and is required, among others, as a substrate for spermidine synthesis. MTA is a methylated side product of spermidine synthesis (Fig. 4a). The similar isotope abundance suggests that the majority of MTA is produced from SAM. We cannot exclude that methyl groups are also used for processes such as purine synthesis (Supplementary Fig. 12); however, results of [^13^C-methyl]DMSP LC–MS analysis are in accordance with the transcriptional upregulation of spermidine synthesis genes in response to DMSP (Fig. 4a). These results show that DMSP methyl groups are assimilated during the bacterial lag phase through the methionine cycle for the synthesis of essential building blocks.

### Differential lag shortening in various bacteria

*P. inhibens* bacteria use algal methylated compounds to reduce methyl group demands during the lag phase, enabling earlier growth. This rapid response has potential significance in environmental contexts when starved bacteria encounter algal hosts. We hypothesized that many algal-associated bacteria share this capability, but it may vary among species to be advantageous. Our findings show that certain algal-associated bacteria reduce lag phases in response to DMSP and betaine, although not universally across species. Moreover, the extent of lag phase reduction varies among bacteria and in response to different methylated compounds (Table 1 and Supplementary Fig. 16).

**Table 1:** Bacteria that are naturally associated with algae and plants exhibit various lag reduction capabilities in response to DMSP and betaine

Bacterial species	Lag reduction (h)	
	DMSP	Betaine
Algal-associated bacteria		
*Sulfitobacter pontiacus* iR4	1.2 (±0.1)	1.6 (±0.1)
*Labrenzia* sp. DT40	4.2 (±0.3)	5.6 (±0.3)
*Vibrio* sp. DT22	1.7 (±0.1)	1.7 (±0.2)
*Phaeobacter* sp. DT104	2.7 (±0.9)	ND
*Pseudoalteromonas* sp. DT35	ND	1.4 (±0.1)
*Labrenzia* sp. DT112	ND	ND
*Vibrio* sp. DT18	ND	ND
*Marinomonas* sp. DT74	ND	ND
Plant-associated bacteria		
*Bacillus subtilis* 168	0.8 (±0.2)	2.1 (±0.1)
*Pseudomonas aeruginosa* PAO1	1.2 (±0.7)	1.5 (±0.2)
*Pseudomonas koreensis* PK	1.5 (±0.6)	ND
*Ewingella americana* EA	ND	ND
*Lelliottia amnigena* LA	ND	ND
*Enterobacter aerogenes* Ea	ND	ND

Values represent the Δlag time of bacteria exposed to 2 µM of DMSP or betaine (compared with controls with water). Values in brackets indicate the standard deviation of the difference between estimated means, using three biological replicates per condition. ND, not detected.

Source data

The synthesis of methylated compounds is a hallmark of photosynthetic organisms, including both algae and plants^36,37^. Therefore, we investigated whether plant-associated bacteria respond similarly to algal-associated bacteria. Our findings show varying lag phase reductions in plant-associated bacteria in response to betaine and DMSP (Table 1 and Supplementary Fig. 17). In addition, we analysed the genomes of these bacterial strains to assess their possession of the 83 methyl metabolism genes with differential expression in *P. inhibens* (Fig. 1). However, no correlation was found between the presence of these genes and lag phase reduction (Supplementary Fig. 18), probably because of technical factors such as incomplete annotation and unavailable precise genomes. For instance, although *Vibrio* bacteria reduce their lag phase in response to DMSP, the key DMSP demethylase gene, *dmdA* (gene 25), is absent from their genome (Supplementary Fig. 18). While previous studies have shown DMSP catabolism in *Vibrio*, the specific enzymes involved remain unidentified^38^. These uncertainties highlight the need for further study to uncover the mechanisms driving lag phase shortening in diverse bacterial strains. Our data indicate that bacteria interacting with photosynthetic organisms can initiate growth earlier using host methylated compounds. The specific bacterial responses to these compounds may be shaped by their natural environments. These findings underscore how previously overlooked bacterial physiologies, like lag reduction, emerge when studied within an ecological framework encompassing factors such as starvation and microbial interactions.

## Discussion

The lag phase, recognized over a century ago, occurs when starved bacterial cultures encounter fresh nutrients. Although observed and defined in controlled laboratory settings, our understanding of its molecular intricacies remains limited. Technical challenges arise because conventional lab methods require high bacterial densities, while the lag phase occurs when populations are sparse at culture onset. Recent advancements shed light on cellular events during the lag phase^10^, yet much is unknown in both model and environmental strains. Analogies can be drawn between lab-based lag phases and potential occurrences in natural settings, where bacteria face nutrient scarcity and periods of no growth^39^. Heterotrophic bacteria that rely on organic compounds from photosynthetic organisms experience resource fluctuations owing to diurnal and seasonal variations in photosynthesis, potentially inducing bacterial lag phases during these transitions. Although direct observation of the lag phase in natural settings is impractical, laboratory model systems help evaluate the environmental relevance of the lag phase by simulating key factors such as starvation and microbial interactions.

Investigating the lag phase requires precisely defining its start and end. In the laboratory, the lag phase begins at culture initiation, but determining its end, when bacteria enter growth, is challenging. Typically, at the population level, the common method involves plotting bacterial growth semilogarithmically and identifying the intersection between the initial inoculum level and the extrapolated slope of the logarithmic growth phase. At the single-cell level, the lag phase ends with the first division. Recent single-cell analysis reveals population heterogeneity, with diverse lag phase phenotypes observed^40^.

Monitoring bacterial population growth and defining lag phases become complex when studying environmental strains. Bacteria such as *P. inhibens* can attach to algal hosts, complicating conventional counting methods when multiple bacteria adhere to an algal cell. Technological advancements enabling single-cell tracking while attached to hosts are crucial for precision. Understanding cellular processes during the lag phase could aid in developing metabolic and genetic markers for studying both populations and single cells.

During algal–bacterial interactions, *P. inhibens* bacteria exhibit substantial methyl group metabolism, particularly during the lag phase. Our data highlight spermidine as a key metabolite during the lag phase, which is produced downstream of the methionine cycle. In DMSP-supplemented bacteria, the *metK* gene, involved in methionine-to-SAM conversion^41^ (a precursor for spermidine), shows pronounced transcriptional upregulation. In addition, the three most upregulated methyl group metabolism genes were associated with the conversion of SAM into spermidine—a pathway that yields MTA as a side product (genes 1, 2 and 21; Supplementary Fig. 11). Notably, ^13^C-labelled methyl groups are incorporated into SAM and MTA during the lag phase (Fig. 4d). Spermidine, a polycationic polyamine, interacts with macromolecules and plays diverse cellular roles^35,42^. Polyamines are associated with protein synthesis^43^, and external supplementation can enhance protein production^42^.

In addition, our results suggest that translation initiation, the crucial step in protein synthesis, could be a destination of methyl groups during lag phase. Translation initiation heavily relies on formylated methionine as the initial amino acid. The production of formylated methionine requires at least two C1 groups, emphasizing the importance of methyl groups during the lag phase. In our transcriptome analyses, the *metG* gene that is responsible for charging tRNA^Met^ with methionine was markedly upregulated in DMSP-supplemented *P. inhibens* bacteria during the lag phase (gene 44; Supplementary Fig. 11). Moreover, we found that various genes involved in adding C1 modifications to tRNA and rRNA components of the translation apparatus were upregulated during the lag phase (for example, genes 39, 41, 42 and 43; Supplementary Fig. 11 and Supplementary Table 3). Thus, while requiring further substantiation, our data suggest a link between methyl group assimilation and translation initiation.

As protein synthesis is a primary cellular activity during the preparatory lag phase, the functional connection between translation initiation and spermidine synthesis in the context of methyl metabolism warrants additional investigation.

The metabolism of algal-associated bacteria is fuelled by algal-produced metabolites. Thus, the bacterial diet naturally includes methylated compounds that are a hallmark for photosynthetic organisms^19,20,36^. Our findings suggest that algal-associated bacteria can efficiently collect and assimilate methyl groups from algal donor compounds. While bacteria are capable of synthesizing methyl groups from glucose, collecting methyl groups from host metabolites can provide a cost-effective metabolic shortcut. In the marine environment, expediting the transition from lag phase to exponential growth is critical to compete with other marine bacteria for valuable algal resources. Specifically, we observed that algal methylated compounds shortened the lag time by up to 2.5 h in *P. inhibens* (Fig. 2c), similar to the doubling time of these bacteria (Supplementary Fig. 7). Consequently, under optimal conditions, bacteria may yield twice as many cells in response to pulses of algal metabolites, compared with bacteria that are not adapted to collecting methyl groups from external compounds. Maximizing the utilization efficiency when resources are available presents a selective advantage to bacteria^44^. Given that different algal hosts synthesize and release a unique composition of metabolites^45,46^, including methylated compounds^47,48^, the differential bacterial response may represent host-specific adaptations. The relationship between the spectrum of methylated compounds produced by specific microalgae and the demethylation abilities of their associated bacteria remains to be explored. Our study shows the tunability of the lag phase, highlighting gaps in our understanding of non-growing bacteria and their transition into growth.

## Methods

Key resources and corresponding references are provided in Supplementary Table 9.

### Statistics and reproducibility

The study design and the statistical analyses of the data were conducted according to common practices in the field. No statistical method was used to predetermine sample size, samples included at least three replicates, no data were excluded from the analyses, all attempts of replication were successful, the samples were allocated randomly and the investigators were blinded to group allocation during data collection and analysis.

### Microalgae, medium composition and general culture conditions

The algal strain *E. huxleyi* CCMP3266 was purchased as an axenic culture from the National Center for Marine Algae and Microbiota (Bigelow Laboratory for Ocean Sciences). The absence of bacteria in axenic algal cultures was monitored periodically both by plating on ½ YTSS agar plates (yeast extract, 2 g l^−1^; trypton, 1.25 g l^−1^; sea salts, 20 g l^−1^; agar, 16 g l^−1^) and by observing under the microscope. The absence of bacteria was additionally confirmed by recent transcriptome sequencing^49^. Algae were maintained in artificial seawater (ASW_a_) medium at 18 °C with a light–dark cycle of 18 h of light and 6 h of dark, and an illumination intensity of 150 mmol m^−^^2^ s^−^^1^. The ASW_a_ medium was based on the protocol of a previous study^50^ and contained mineral salts (NaCl, 409.41 mM; Na_2_SO_4_, 28.22 mM; KCl, 9.08 mM; KBr, 0.82 mM; NaF, 0.07 mM; Na_2_CO_3_, 0.20 mM; NaHCO_3_, 2 mM; MgCl·6H_2_O, 50.66 mM; CaCl_2_, 10.2 mM; SrCl_2_·6H_2_O, 0.09 mM), f/2 vitamins (thiamine HCl, 100 μg l^−1^; biotin, 0.5 μg l^−1^; vitamin B_12_, 0.5 μg l^−1^), L1 trace elements (Na_2_EDTA·2H_2_O, 4.36 mg l^−1^; FeCl_3_·6H_2_O, 3.15 mg l^−1^; MnCl_2_·4H_2_O, 178.1 μg l^−1^; ZnSO_4_·7H_2_O, 23 μg l^−1^; CoCl_2_·6H_2_O, 11.9 μg l^−1^; CuSO_4_·5H_2_O, 2.5 μg l^−1^; Na_2_MoO_4_·2H_2_O, 19.9 μg l^−1^; H_2_SeO_3_, 1.29 μg l^−1^; NiSO_4_·6H_2_O, 2.63 μg l^−1^; Na_3_VO_4_, 1.84 μg l^−1^; K_2_CrO_4_, 1.94 μg l^−1^) and L1 nutrients (NaNO_3_, 882 μM; NaH_2_PO_4_·2H_2_O, 36.22 μM). The components were dissolved in Milli-Q water (IQ 7003; Merck) and the pH adjusted to 8.2 with HCl. Stock solutions of f/2 vitamins, L1 trace elements and L1 nutrients were purchased from the Bigelow Laboratory for Ocean Sciences (Boothbay, ME, USA).

### Marine bacteria and medium composition

The bacterial strain *Phaeobacter inhibens* DSM 17395 was purchased from the German Collection of Microorganism and Cell Cultures (DSMZ, Braunschweig, Germany). The bacterial strain *Sulfitobacter pontiacus* iR4 was isolated from a xenic culture of *E. huxleyi* CCMP1516 (ref. ^51^). The bacterial strains *Vibrio* sp. DT22, *Labrenzia* sp. DT40, *Phaeobacter* sp. DT104, *Pseudoalteromonas* sp. DT35, *Labrenzia* sp. DT112, *Vibrio* sp. DT18 and *Marinomonas* sp. DT74 were isolated from the Mediterranean Sea and Red Sea^52^, and belong to the Tawfik Lab marine bacteria collection, which is now part of the Segev Lab strain collection at the Weizmann Institute of Science, Israel. The marine bacteria were cultivated in ASW_b_ medium (identical to the ASW_a_ medium, but with additional 5 mM NH_4_Cl and 33 mM Na_2_SO_4_; f/2 vitamins were omitted and glucose added as indicated), filtered seawater medium (Mediterranean Sea water enriched with L1 trace elements, f/2 vitamins and L1 nutrients in the same concentrations as used for ASW_a_) or ½ YTSS medium (yeast extract, 2 g l^−1^; trypton, 1.25 g l^−1^; sea salts, 20 g l^−1^), as indicated. Bacterial isolates were kept at −80 °C in ½ YTSS medium with 20% glycerol for long-term storage.

### Non-marine bacteria and medium composition

The bacterial strains *Pseudomonas koreensis* PK, *Pseudomonas aeruginosa* PAO1, *Ewingella americana* EA, *Lelliottia amnigena* LA and *Enterobacter aerogenes* Ea were provided by Jonathan Friedman (The Hebrew University of Jerusalem, Israel)^53^. *Bacillus subtilis* strain 168 was obtained from the Bacteriology Unit of the Weizmann Institute of Science, Israel. The strains were cultivated in M9 medium (Na_2_HPO_4_·7H2O, 64 g l^−1^; KH_2_PO_4_, 15 g l^−1^; NH_4_Cl, 5 g l^−1^; NaCl, 2.5 g l^−1^) with 20 mM glucose. Bacterial isolates were kept at −80 °C in LB medium (tryptone, 10 g l^−1^; yeast extract, 5 g l^−1^; NaCl, 10 g l^−1^ NaCl) with 20% glycerol for long-term storage.

### Co-culture RNA-sequencing

#### Experimental design

To analyse the transcriptome of *P. inhibens* DSM 17395 bacteria during co-cultivation with the *E. huxleyi* CCMP3266 algae, a dual RNA-sequencing approach was applied that allows probing prokaryotic and eukaryotic RNA in the same samples, without separating cells by, for example, filtration^54^. For cultivation, 250 ml Erlenmeyer flasks with 60 ml ASW_a_ medium were inoculated with either both organisms (co-cultures) or one of the two organisms (pure cultures). Specifically, 24 flasks were inoculated with both organisms (20,000 *E. huxleyi* cells and 2,000 *P. inhibens* CFUs; bacteria were pre-cultivated for 48 h in ASW_b_ medium with 2 mM glucose and f/2 vitamins, and added to algae 4 days after inoculation). Another 24 flasks were inoculated with a pure culture of algae (20,000 *E. huxleyi* cells). All 48 flasks were sealed with aluminium foil and cultivated in standing cultures in a growth room at 18 °C under a light–dark cycle of 16 h of light and 8 h of dark. The illumination intensity during the light period was 150 mmol m^−^^2^ s^−1^. At each sampling point, the cultures in three flasks selected randomly per condition (three algal–bacterial co-culture flasks and three algal pure-culture flasks) were sacrificed and sampled to enumerate algal cells and bacterial CFUs and to collect cells for RNA extraction (starting from day 0; see Supplementary Fig. 1 for sampling points). Nine additional replicate flasks were inoculated with pure cultures of bacteria (2,000 *P. inhibens* CFUs; 0.88 mM NH_4_Cl and 2 mM glucose were added to the ASW_a_ medium to facilitate bacterial growth). The nine bacterial flasks were cultivated under the same conditions as the flasks with algae. Three flasks were repeatedly sampled to enumerate bacterial CFUs, while the cultures in three flasks were sacrificed per sampling point to collect cells for RNA extraction (see Supplementary Fig. 1 for growth curves and RNA sampling points).

Algal cell numbers were determined for each sampled flask as technical triplicates, using a CellStream flow cytometer (Merck). Bacterial CFU numbers were determined for each flask as technical duplicates, using serial plating on ½ YTSS medium agar plates. To collect cells for RNA extraction, 50 ml of culture was centrifuged for 5 min at 18 °C and 3,220 *g* (5810 R swing-out centrifuge; Eppendorf) and the supernatant was removed using a vacuum suction. Cell pellets were resuspended in 450 µl RNA lysis buffer (RLT; QIAGEN) with 1% β-mercaptoethanol, transferred into a 2 ml screw-capped tube with 300 mg of 100 µm acid-washed Low Binding Silica Beads (SPEX SamplePrep), plunged into liquid nitrogen and stored at −80 °C for 1 month until use.

#### RNA extraction and library preparation

RNA extracts were generated from 30 selected cell pellets (3 biological replicates per sampling point) that originated from algal–bacterial co-cultures, axenic algae and bacteria grown in pure culture with 2 mM glucose (see Supplementary Fig. 1 for sampling points and sample designations). The cell pellets were disrupted by 5 min of bead beating at 30 s^−1^ in a mixer mill MM 400 (Retsch). RNA was extracted from disrupted cells using the ISOLATE II RNA Mini Kit, which integrates a step for an on-column DNase treatment (Meridian Bioscience). The extracted RNA was subjected to a second DNAse treatment using the TURBO DNase kit (Thermo Fisher Scientific) and subsequently purified with 2× concentrated RNAClean XP magnetic beads (Beckman Coulter). RNA quantity and integrity were evaluated with the Qubit RNA HS Assay (Invitrogen, Thermo Fisher Scientific) and the Tapestation RNA ScreenTape analysis (Agilent Technologies), respectively (Supplementary Fig. 2). Ribosomal RNA was depleted using the riboPOOL rRNA depletion kit in combination with Pan-Bacteria probes and custom probes generated for *E. huxleyi* rRNA from the nucleus, plastid and mitochondrion (siTOOLs Biotech). For ribosomal RNA depletion, 0.9 µg of RNA was used as an input with 50 pmol of each of the two depletion probes. After rRNA depletion, RNA was purified with 2× concentrated RNAClean XP magnetic beads (Beckman Coulter). Subsequent library preparation was performed as described^54^, which is a stranded, poly(A)-independent protocol that targets total RNA. Briefly, depleted RNA was fragmented by heat, treated with DNase and FastAP, and then 3′-tagged by ligating barcoded adapters 1 to 30 (ref. ^54^) (RNase-free high-performance liquid chromatography purified; ordered from Integrated DNA Technologies). After barcoding, we implemented a calibration sequencing run to quantify the relative abundance of bacterial RNA in each sample. This quantification allows determining the optimal sample-specific sequencing depths and prevents over-sequencing of samples that contain either dead algae or only one of the two organisms. For the calibration RNA-sequencing run, we proceeded with half of the barcoded RNA and stored the rest at −80 °C. All 30 samples were pooled in an equivolume and purified with RNA Clean & Concentrator-5 columns (Zymo Research). RNA was reverse transcribed with the SuperScript IV First-Strand Synthesis System (Invitrogen, Thermo Fisher Scientific), using the AR2 primer^54^. The resulting cDNA was 3′-ligated to the 3Tr3 adaptor^54^ (HPLC purified; ordered from Integrated DNA Technologies). The 3′-ligated cDNA was then amplified with 9 PCR cycles (see Supplementary Fig. 19a for PCR cycle optimization). Finally, the amplified cDNA was purified with a double-sided clean-up step (right: 0.5×, left: 1.5×; RNAClean XP beads, Beckman Coulter), followed by a left-sided clean-up step (0.7×). The size distribution of the final calibration sequencing library is shown in Supplementary Fig. 19b. After the clean-up, the library was sequenced on a NextSeq 500 instrument with a 150-cycle Mid Output Kit (Illumina) in paired-end mode (read 1: 88 bp, read 2: 78 bp, index 1: 0 bp, index 2: 0 bp). Sequencing data were analysed for the relative abundance of algal and bacterial RNA per sample, using bioinformatics methods described in the section below. The library preparation protocol was then repeated for the final co-culture RNA-sequencing run, starting from the stored, barcoded RNA. The barcoded RNA was pooled in a way that co-culture samples with algae are expected to yield equal amounts of bacterial sequencing reads (Supplementary Fig. 1; co-culture day 4, 6 and 9; expected bacterial feature counts per sample: 250,000–500,000), while fewer sequencing reads were allocated to algal pure cultures (axenic day 4, 6, 9 and 11/12; expected algal feature counts per sample: 40,000,000), co-cultures with bleached algae (co-culture day 11/12; expected algal feature counts per sample: 25,000,000) and bacterial pure cultures (glucose 1 and glucose 2; expected bacterial feature counts per sample: 5,000,000). After reverse transcription and ligation, the 3′-ligated cDNA was amplified with 11 PCR cycles (see Supplementary Fig. 20a for PCR cycle optimization). The final co-culture RNA-sequencing library (Supplementary Fig. 20b) was deep sequenced on a NovaSeq 6000 instrument with a 100-cycle S2 Kit (Illumina) in paired-end mode (read 1: 64 bp, read 2: 54 bp, index 1: 0 bp, index 2: 0 bp; Supplementary Table 1). The expected distribution of algal and bacterial sequencing reads per sample was confirmed as described below (see Supplementary Table 2 for resulting algal and bacterial feature counts per sample).

#### Data analysis

Illumina raw reads were demultiplexed with fastq-multx^55^, allowing one mismatch per barcode (v1.3.1; -m 1). Raw reads were then quality filtered and trimmed with cutadapt^56^ (v1.18; -a AGATCGGAAGAGCACACGTCTGAACTCCAGTCAC -A AGATCGGAAGAGCGTCGTGTAGGGAAAGAGTGT -g T{20} -A A{20}–times 2–nextseq-trim=20 -m 20–pair-filter=both; see Supplementary Table 2 for summary; reads were deposited under the BioProject accession PRJNA976961). The quality-filtered and trimmed reads were mapped to a reference fasta file that included the previously generated ‘synthetic genome’ assembly (sGenome) of *E. huxleyi* CCMP3266 (ref. ^49^) and the genome of *P. inhibens* DSM 17395 (accession: GCF_000154765.2). Read mapping was conducted with STAR^57^ (v2.7.5c; –outSAMattributes All –alignIntronMax 5000 –alignMatesGapMax 5000 –outSAMtype BAM SortedByCoordinate –outFilterMultimapNmax 100 –outSAMmultNmax 1). For compatibility with STAR, empty lines generated by cutadapt were replaced with N’s (sed ‘s/^$/N/’). Reads that mapped to *E. huxleyi* features were counted using featureCounts from the subread package^58^ (v2.0.0; -p -C -a Ehux3266_sGenome_v2.gff -F GTF -O –fraction; see Supplementary Data 3 for the annotation file). Of note, a slightly modified version of the previously generated *E. huxleyi* CCMP3266 annotation file was used with featureCounts^49^, in which plastid and mitochondrion annotations were replaced by public annotations (accessions: JN022705.1 and JN022704.1, respectively). For compatibility with featureCounts, the NH:i attribute of STAR bam files was changed to ‘1’ (sed -r ‘s/NH:i:[0–9]+/NH:i:1/g’; STAR reports the number of alternative alignments in the NH:i attribute, which interferes with featureCounts fractional counting). Reads that mapped to *P. inhibens* features were counted with the same featureCounts options but using the *P. inhibens* RefSeq annotations. The output of featureCounts is summarized in Supplementary Table 2. To evaluate the efficiency of ribosomal RNA depletion, also non-rRNA features were summarized (Supplementary Table 2), in which algal rRNA from the nucleus (*E. huxleyi* CCMP3266 sGenome gene ID: G8853), the plastid (Plas_rrn5, Plast_rrnL, Plast_rrnS) and the mitochondrion (Mito_rrnL, Mito_rrnS), as well as bacterial 23S rRNA (PGA1_RS00070, PGA1_RS12745, PGA1_RS14220, PGA1_RS16070), 16S rRNA (PGA1_RS00055, PGA1_RS12760, PGA1_RS14235, PGA1_RS16085) and 5S rRNA (PGA1_RS00075, PGA1_RS12740, PGA1_RS14215, PGA1_RS16065) were excluded.

Bacterial gene transcription was analysed only in co-culture RNA-sequencing samples that contained *P. inhibens* (Supplementary Table 2 and Supplementary Fig. 1). As sample-specific rRNA depletion efficiencies may skew results, bacterial rRNA features were removed from the feature count table for downstream analysis. To analyse sample variability, bacterial non-rRNA feature count results were normalized for sequencing depth and RNA composition using the median of ratios method of DESeq2, followed by a regularized log transformation and principal component analysis^59^ (v1.36.0; blind = TRUE; ntop = 500; R version 4.2.0; Supplementary Fig. 3). DESeq2 was further used to identify differentially expressed genes by comparing samples of bacteria growing with algae (co-culture day 4, 6 and 9; Supplementary Figs. 1 and 3) with samples of bacteria growing exponentially in pure cultures (glucose 1; Supplementary Figs. 1 and 3). The apglm method was used to shrink log_2_ fold changes and account for overweighing of features with low counts and high variability^60^. Shrunken log_2_ fold changes and adjusted *P* values are given in Supplementary Data 1. To compare absolute transcript abundances between genes and samples, bacterial feature counts were converted to transcripts per kilobase million (TPM), a normalization method that accounts for gene length and sequencing depth (Supplementary Data 1). To identify the 20 highest-transcribed metabolic genes, the *P. inhibens* feature table (Supplementary Data 1) was filtered for genes that are part of the KEGG metabolic pathway annotation map^61^ (pga01100; https://www.genome.jp/pathway/pga01100). The resulting 840 KEGG metabolic genes were filtered for the 20 genes with the highest mean transcript abundances in samples with algae (co-culture day 4, 6 and 9).

To identify methyl group metabolism genes in *P. inhibens*, publicly available functional annotations were added to the feature table (Supplementary Data 1). These annotations include automatically generated NCBI RefSeq annotations^62^, partially curated GenBank submitter annotations^63^ and KEGG annotations^61^, as well as links to BioCyc^64^ and the unifying UniProt Archive (UniParc^65^; links redirect to, for example, pre-computed domain annotations at the InterPro database^66^). Features of *P. inhibens* were then filtered for relevant genes, based on differential expression patterns (adjusted *P* ≤ 0.05; log_2_(fold change) ≤ −0.585 and ≥0.585; Supplementary Data 1). The DE-filtered genes were manually curated by sourcing the public annotations, which identified methyl group metabolism genes that are involved in transporting and catabolizing methylated compounds; in collecting, dissimilating or assimilating one-carbon groups; or in synthesizing THF. Also, genes that are involved in the ethylmalonyl-CoA pathway were included, which replenishes the TCA cycle by capturing CO_2_ during growth on the methylated compound DMSP^67^. Complementary to this approach, methyl group metabolism genes were identified by revising literature on one-carbon metabolism^68,69^ and searching for homologue genes in *P. inhibens* using nucleotide and protein BLAST^70^. In the latter approach, genes were considered as relevant if they exhibited ≥50 mean TPM in at least one of the sampling points. The resulting list, which includes 83 curated *P. inhibens* methyl group metabolism genes, is given in Supplementary Table 3. For clarification, genes were excluded from the list that did not meet the above-described cut-off filters, such as a putative DMSP cleavage enzyme (*dddP*; PGA1_RS09315) that was poorly transcribed in all samples (<50 TPM), and non-regulated methyltransferases.

### Growth response in bacteria to supplements

To test the effect of methylated compounds on the growth of *P. inhibens* wild type and KO mutant with a deleted *dmdA* gene (Fig. 3c), bacteria were streaked from glycerol stocks on ½ YTSS medium agar plates and incubated at 30 °C for 48 h. After incubation, a single colony was used to inoculate a pre-culture with 10 ml ASW_b_ medium (1 mM glucose, 100 ml Erlenmeyer flasks). The pre-culture was cultivated at 30 °C with 130 rpm shaking for 48 h. After 48 h, the pre-culture was in the stationary phase and exhibited OD_600_ ≈ 0.1. The stationary phase pre-culture was used to inoculate the main cultures (1 ml ASW_b_ medium, 1 mM glucose, start OD_600_ = 0.00001 (based on OD_600_ in pre-culture and applied dilution factor)), which contained supplements as indicated (mainly 2 µM of a methylated compound; listed in the key resources table). Supplements were prepared as 50 mM stock solutions in Milli-Q water and sterilized by filtration with syringe filters (Millex-GV, 0.22 µm, PVDF, Merck). Each inoculated main culture was divided into three or four different wells (biological replicates) of a 96-well microtitre plate (150 µl main culture per well) and overlaid with 50 µl hexadecane to prevent evaporation^71^. Cultivation of the main cultures was conducted at 30 °C in an Infinite 200 Pro M Plex plate reader (Tecan Group) with alternating cycles of 4 min of shaking and 16 min of incubation. Optical density measurements were conducted at 600 nm following the shaking step.

The same growth protocol was used to analyse growth responses when supplemented with 2 µM of the methylated compounds DMSP or betaine, or water as control in the marine bacteria *Vibrio* sp. DT22, *Labrenzia* sp. DT40, *Phaeobacter* sp. DT104, *Pseudoalteromonas* sp. DT35, *Labrenzia* sp. DT112, *Vibrio* sp. DT18 and *Marinomonas* sp. DT74 (Supplementary Fig. 16). A slightly modified protocol was used for *S. pontiacus* iR4 marine bacteria, in which ASW_b_ medium was replaced by filtered seawater medium (Supplementary Fig. 16). To study the responses to 2 µM DMSP or betaine, the plant-associated bacteria *B. subtilis* 168, *Pseudomonas koreensis* PK, *Pseudomonas aeruginosa* PAO1, *Ewingella americana* EA, *Lelliottia amnigena* LA and *Enterobacter aerogenes* Ea were grown in M9 minimal media supplemented with 20 mM glucose (Supplementary Fig. 17).

### Growth data analysis

For data analysis, microtitre plate absorption values were converted to OD_600_ values by multiplying measured values with a factor of 3.8603. This factor was determined by comparing the absorption (at 600 nm) of a culture sample in a spectrophotometer using a 1 cm cuvette (Ultrospec 2100 Pro, Biochrom) with the absorption in the microtitre plate reader (150 µl culture + 50 µl hexadecane per well). The background absorption, which is the mean absorption during the non-growth phase with stable OD_600_ values (≈2–8 h), was subtracted from each individual well. Results were plotted in R (v4.2.0) using ggplot2 (v3.4.1). As a visual aid, smoothing lines were added that average the replicates for each condition (geom_smooth; method: loess). Doubling times and lag times were calculated for each replicate using the Growthcurver package^72^ (v0.3.1). For this package, background subtracted OD_600_ values were used as an input, which span the time from the lowest to the highest measured absorptions. Growthcurver was used to fit the OD_600_ values into a logistic equation that describes the population size *N*_*t*_ (OD_600_) at time *t*. The output of Growthcurver includes the modelled growth rate *r*, the carrying capacity *K* and the initial population size *N*_0_:

$${N}_{t}=\frac{K}{\left[1+\left(\frac{K-{N}_{0}}{{N}_{0}}\right){e}^{-r t}\right]}$$

To calculate lag times, we formulated a definition in which lag times equal the time *t* at which *N*_*t*_ (OD_600_) reaches an absorption of 0.01. Using this definition, the logistic equation of Growthcurver was rearranged to *t* and solved with *N*_*t*_ = 0.01:

$$t=\left(-\frac{1}{r}\right)\left[\mathrm{ln}\left(\frac{K-{N}_{t}}{K-{N}_{0}}\right)-\mathrm{ln}\left(\frac{{N}_{t}}{{N}_{0}}\right)\right]$$

To calculate Δlag times, we first determined the mean lag time for each condition. Then, the mean lag times of supplemented treatment conditions were subtracted from the mean lag time of the control condition. Thus, the resulting Δlag times are a measure for the effect of supplements on the length of the lag phase. To determine the standard deviation of the estimated difference between means *σ*_*M*1−*M*2_, a formula was used that incorporates the standard deviation for the lag time of the treatment condition (*σ*_1_), the control condition (*σ*_2_) and the number of replicates *n* per condition:

$${\sigma }_{M1-M2}=\sqrt{\frac{{\sigma }_{1}^{2}}{{n}_{1}}+\frac{{\sigma }_{2}^{2}}{{n}_{2}}}$$

Doubling times were calculated by solving the logistic equation of Growthcurver with *N*_*t*_ = 2*N*_0_. The R package multcomp^73^ (v1.4-23) was used to identify significant differences between lag times and doubling times. Specifically, an ANOVA model (aov function) was fitted to the two growth parameters, followed by a Dunnett’s test (glht function).

### Knock-out mutants with deleted *dmdA*

Primers and plasmids used to generate *P. inhibens* KO mutants are listed in the key resources table. PCR amplification and restriction-free cloning^74^ were performed using the Phusion High Fidelity DNA Polymerase (Thermo Fisher Scientific). PCR products were purified with the NucleoSpin Gel and PCR Clean-Up Kit (MACHEREY-NAGEL). Plasmids were purified with the QIAprep Spin Miniprep Kit (QIAGEN).

To generate KO mutants for the *dmdA* gene (accession: PGA1_RS19020; gene 25), we first PCR-amplified ~1,000 bp upstream and downstream regions of the respective loci (primers 1–4). A gentamycin resistance gene was amplified from the plasmid pBBR1MCS-5 (primers 7–8). The PCR products (upstream region + gentamycin resistance + downstream region) were then assembled and cloned into the pCII-TOPO vector (Invitrogen, Thermo Fisher Scientific) using restriction-free cloning^74^. This resulted in the KO plasmid pDN12 (*dmdA*). For transformation, 10 µg of KO plasmid was added to 300 µl of competent *P. inhibens* cells (prepared using the method in ref. ^75^) and electroporated with a pulse of 2.5 kV (MicroPulser, Bio-Rad Laboratories). Electroporated cells were recovered in 2 ml ½ YTSS medium for 12 h at 30 °C and transferred to selective ½ YTSS medium agar plates with 30 µg ml^−1^ gentamycin. To screen for transformants, single colonies were used as template for PCRs (primers 5–6 and 9–10). Gene KOs were additionally verified in single-cell clones by Sanger sequencing.

### Stoichiometric calculations

To estimate the amount of methyl group requirements per *P. inhibens* cell during growth on glucose, we identified metabolites that are built from C1 groups^76,77^ and are thus C1 sinks. The focus was on C1 sinks that are used as building blocks to construct macromolecules such as DNA, RNA and proteins, and for which quantitative data are available in *E. coli*^32^. We identified seven C1 sinks in *P. inhibens* (adenosine triphosphate (ATP), guanosine triphosphate (GTP), deoxyadenosine triphosphate (dATP), deoxyguanosine triphosphate (dGTP), deoxythymidine triphosphate (dTTP), histidine and methionine; Supplementary Table 5). Not included were serine, phosphatidylcholine, spermidine, macromolecule methylations and low-abundance metabolites. Serine is an important C1 group donor in cells (Fig. 1; gene 67); however, serine synthesis from glucose does not consume C1 groups. Phosphatidylcholine has a choline backbone with three *N*-methyl groups and is produced by many bacteria^78^. It was reported that some *Phaeobacter* species produce phosphatidylcholine^79^; however, only minor amounts of this lipid were detected in *P. inhibens* DSM 17395 during growth with glucose^80^. The metabolite spermidine was excluded owing to uncertainties about methyl group recycling. The bacterium *P. inhibens* possesses all genes required to synthesize spermidine by first decarboxylating SAM to *S*-adenosyl 3-(methylsulfanyl)propylamine (dcSAM), and then condensing dcSAM with putrescine to spermidine (Fig. 4a). Importantly, the *S*-methyl group of dcSAM is not directly incorporated into spermidine but remains attached to a side product of spermidine synthesis, which is MTA (Fig. 4a). While the *S*-methyl group of MTA is recycled in other bacteria via the methionine salvage pathway, this pathway appears to be incomplete in *P. inhibens*. Thus, the incomplete methionine salvage pathway may result in the loss of one methyl group per synthesized spermidine molecule. However, considering that a yet unknown methionine salvage pathway may exist in *P. inhibens*^81^, spermidine was not included as a C1 sink (Supplementary Table 5). Methylated forms of the macromolecules DNA^82^, RNA^83^ and proteins^84^ were also not included because, to the best of our knowledge, no robust data exist on the quantity of macromolecule methylations in the cell. Lastly, not included were low-abundance C1 sinks such as pantothenate (required for CoA synthesis) or NAD/NADP, which represent negligible fractions of the bacterial biomass^32^. After identifying C1 sinks in *P. inhibens*, we gathered data on the amount of the respective sinks present in *E. coli* (Supplementary Table 5). The values represent *E. coli* cells grown at 37 °C in aerobic glucose minimal medium and at a doubling time of 40 min (ref. ^32^). A single *E. coli* cell has a dry weight of 280 fg under these conditions, which we used to calculate the amount of C1 sinks present per cell (amol building blocks per cell; Supplementary Table 5). We then multiplied the amount of C1 sinks per cell with the net amount of C1 groups that are incorporated into each of the sinks (amol C1 groups per cell; Supplementary Table 5). By summing up the results for the individual C1 sinks, we estimated that a single *E. coli* cell requires 190.1 amol C1 groups to synthesize its building blocks. To calculate the C1 group requirement per *P. inhibens* cell, we assumed that *P. inhibens* and *E. coli* produce the same amounts of the respective C1 sinks. This assumption is corroborated by the fact that *P. inhibens* is, like *E. coli*, a copiotrophic bacterium^85^ with a single cell length of 1–2 µm (ref. ^79^). To estimate ATP costs, we retrieved the growth-associated ATP maintenance costs (GAM) from the metabolic model of *P. inhibens*, which is given as 85 mmol ATP per gram dry weight^33^. Based on a dry weight of 280 fg per cell^32^, this equals 23,800 amol ATP per *P. inhibens* cell.

### Lag phase RNA-sequencing

To analyse transcriptional changes induced by DMSP during the lag phase of *P. inhibens*, a glycerol stock of these bacteria was streaked on a ½ YTSS medium agar plate and incubated at 30 °C for 48 h. After incubation, a single colony was used to inoculate four pre-culture flasks, each containing 100 ml ASW_b_ medium with 1 mM glucose. The four pre-cultures were incubated for 48 h (30 °C, 130 rpm shaking), resulting in stationary phase bacteria (OD_600_ ≈ 0.1). The stationary pre-cultures were then pooled and washed by centrifugation (5 min, 3,220 *g* and 4 °C) using a 5810 R swing-out centrifuge (Eppendorf). After centrifugation, the supernatant was removed using a vacuum suction, and the pellets were resuspended in ASW_b_ medium (without glucose). A second washing step was performed, after which the pellets were resuspended in 30 ml ASW_b_ medium (without glucose) to concentrate the cells. The concentrated cells (OD_600_ = 0.82) were used to inoculate six main culture flasks with DMSP (50 µM) and six flasks without DMSP. Each flask contained 60 ml ASW_b_ medium (1 mM glucose, pre-warmed at 30 °C, start OD_600_ = 0.01). The 12 inoculated main culture flasks were incubated at 30 °C with 130 rpm shaking. Six flasks (three flasks with 50 µM DMSP and three flasks without DMSP) were collected 15 min after inoculation for RNA extraction. The other six flasks were collected 40 min after inoculation. Separate cultivation experiments were conducted to confirm that DMSP stimulates growth under the applied conditions (Supplementary Fig. 8). The protocol used for cell collection and RNA extraction is described in the section RNA Extraction and Library Preparation with the adaptation that cells were centrifuged at 4 °C. Results of the RNA extraction are shown in Supplementary Fig. 9. All 12 extracted RNA samples were subjected to RNA library preparation as described in the same section, but with minor modifications. RNA fragmentation was conducted with 300 ng RNA per sample, and rRNA depletion was performed after the pooling step, using 100 pmol Pan-Bacteria probes. Of note, the 3′-ligated cDNA was amplified with 12 PCR cycles (see Supplementary Fig. 21a for PCR cycle optimization). The resulting lag phase RNA-sequencing library (Supplementary Fig. 21b) was sequenced on a NextSeq 500 instrument with a 150-cycle Mid Output Kit (Illumina) in paired-end mode (read 1: 89 bp, read 2: 79 bp, index 1: 0 bp, index 2: 0 bp; Supplementary Tables 6 and 7).

The pipeline described in the section Data Analysis was used to analyse lag phase RNA-sequencing data. Quality-filtered and trimmed sequencing reads (deposited under the BioProject accession PRJNA977030) were mapped to a reference fasta file that contained only the genome of *P. inhibens* DSM 17395 (accession GCF_000154765.2). After the *P. inhibens* non-rRNA feature count table was generated, the data were used as input with the DESeq2 PCA plot function to confirm close clustering of replicates (Supplementary Fig. 10). DESeq2 was also used to compare gene expression in DMSP-supplemented samples with control samples 15 min and 40 min after inoculation (Supplementary Fig. 11). The DE results were included in the TPM-normalized non-rRNA feature count table (Supplementary Data 2).

### DMSP demethylation from cell crude extracts

Enzymatic DMSP demethylation was measured in cell crude extracts using a method adapted from published protocols^86,87^. A glucose-grown, stationary phase pre-culture of *P. inhibens* bacteria was used to inoculate eight main culture flasks (100 ml ASW_b_ medium, 5.5 mM glucose). The main cultures were then cultivated for 4 days (30 °C, 130 rpm) until reaching stationary phase (OD_600_ ≈ 1.0). Four stationary phase main culture flasks were supplemented with 2 µM DMSP (100 µl from a sterile 2 mM DMSP stock solution, dissolved in Milli-Q water) while the other four flasks were kept as non-supplemented controls (adding 100 µl Milli-Q water). Cells were collected 1 h and 2 h after supplementation by centrifugation (5 min, 3,220 *g*, 4 °C), and pellets were resuspended in 1 ml solution A (100 mM HEPES–KOH (pH 7.5), 1 mM DTT; all steps were conducted on ice, using pre-cooled solutions at 4 °C). Resuspended cells were then washed twice with solution A by centrifugation (5 min, 10,000 *g*, 4 °C). To disintegrate cell membranes, resuspended cells were transferred into 2 ml screw-capped tubes filled with 600 mg of 100 µm acid-washed Low Binding Silica Beads (SPEX SamplePrep), plunged into liquid nitrogen, thawed and disrupted by 5 min of bead beating at 30 s^−1^ in a mixer mill MM 400 (Retsch). Disrupted cells were centrifuged (5 min, 15,000 *g*, 4 °C) and the supernatant transferred into a new tube, resulting in the cell crude extract. Protein concentrations were measured using the Protein Assay Dye Reagent Concentrate (Bio-Rad Laboratories) and diluted to 0.7 mg ml^−1^ with solution A. Enzymatic reactions were performed by mixing 0.5 ml of 0.7 mg ml^−1^ diluted protein (cell crude extract) with 1 ml of solution B (20 mM HEPES–KOH (pH 7.5), 3 mM homocysteine, 300 µM DMSP, 2 mM DTT), resulting in 0.35 mg protein per 1.5 ml reaction mix. The reaction mixes were incubated for 1 h at 30 °C. After incubation, 500 µl of the enzymatic reactions was transferred into fresh tubes, plunged in liquid nitrogen and lyophilized for subsequent metabolite extraction and LC–MS analysis, as described in the section Lag Phase LC–MS Analysis with [13C-Methyl]DMSP (but in negative ionization mode). The identification of 3-MMPA was confirmed using an authentic standard.

### Lag phase LC–MS analysis with [^13^C-methyl]DMSP

To analyse DMSP methyl group assimilation during the lag phase, [^13^C-methyl]DMSP, which contained two ^13^C-labelled methyl groups, was synthesized in the Organic Synthesis Unit, Chemical Research Support, Weizmann Institute of Science, Israel. Labelled [^13^C-methyl]DMSP was prepared according to a previous study^88^. The synthesis was carried out by the acid-catalysed addition of [^13^C_2_]dimethyl sulfide to acrylic acid, using the experimental conditions described in the literature for the deuterium-labelled compound. The ^13^C-labelling was confirmed by ^1^H NMR, based on the appearance of the methyl signals as a doublet centred at *δ* = 3 ppm, the typical ^1^H-^13^C J coupling constant of 145 Hz and the mass spectrum showing the molecular ion at *m*/*z* 137. The NMR and the mass spectra did not show a signal for unlabelled DMSP.

Growth conditions for experiments with [^13^C-methyl]DMSP were comparable to those applied for lag phase RNA-sequencing. Briefly, pre-cultures of *P. inhibens* were cultivated for 48 h (100 ml ASW_b_ medium, 1 mM glucose) and washed twice by centrifugation. A concentrated pre-culture pool of stationary phase bacteria was used to inoculate eight main culture flasks (60 ml ASW_b_ medium, 1 mM glucose; start OD_600_ = 0.01)—four flasks contained 50 µM [^13^C-methyl]DMSP and four flasks contained 50 µM non-labelled DMSP. The eight main culture flasks were collected 2 h after inoculation by centrifugation (10 min, 3,220 *g*, 4 °C; see Supplementary Fig. 8 for growth curves), and cell pellets were subsequently snap-frozen in liquid nitrogen. The selection of the 2 h time point was informed by the observation of higher DMSP demethylation activity in crude extracts of stationary phase bacteria supplemented with 2 µM DMSP (Fig. 4b).

Extraction and analysis of polar metabolites were performed as previously described^89^^,^^90^ with some modifications. The bacterial pellets were extracted with 1 ml of a pre-cooled (−20 °C) homogenous methanol:methyl *tert*-butyl ether (MTBE) 1:3 (v/v) mixture. The tubes were vortexed and sonicated for 30 min in an ice-cold sonication bath (taken for a brief vortex every 10 min). Then, 0.5 ml of a ultra-performance liquid chromatography (UPLC)-grade water:methanol (3:1, v/v) solution with 1:500 diluted ^13^C- and ^15^N-labelled amino acid standard mix (Sigma-Aldrich, Merck) was added to the tubes, followed by vortexing and centrifugation. The upper organic phase was discarded. The polar phase was re-extracted with 0.5 ml of MTBE. The lower polar phase was then dried under a gentle stream of nitrogen and kept at −80 °C for metabolite analysis. Dry polar samples were resuspended in 80 µl methanol:double-distilled water (DDW,50:50) and centrifuged twice to remove the debris. The resuspended samples (40 µl) were transferred to HPLC vials for injection.

LC–MS metabolic profiling was done as described previously^90^ with minor modifications. Briefly, analysis was performed using an Acquity I class UPLC System combined with a Q Exactive Plus Orbitrap mass spectrometer (Thermo Fisher Scientific), operated in positive and negative ionization modes. The LC separation was done using a SeQuant Zic-pHilic column (150 mm × 2.1 mm) with a SeQuant guard column (20 mm × 2.1 mm; Merck). Mobile phase B contained acetonitrile, and mobile phase A contained 20 mM ammonium carbonate with 0.1% ammonia hydroxide in water:acetonitrile (80:20, v/v). The flow rate was kept at 200 μl min^−1^, and the gradient was as follows: 0–2 min, 75% of B; 14 min, 25% of B; 18 min, 25% of B; 19 min, 75% of B; for 4 min, 23 min 75% of B. Data processing was done using the Compound Discoverer software (v3.3; Thermo Fisher Scientific); when detected, compounds were identified using accurate mass, retention time, isotope pattern and fragments (Supplementary Data 4). The software reports the fractional label incorporation (exchange rate) after natural abundance correction for each compound. LC–MS data were manually searched for metabolites involved in methyl group metabolism. This included DMSP, 3-MMPA, SAM, dcSAM, MTA, methionine, serine, glycine, 5-methyltetrahydrofolate, 5,10-methylenetetrahydrofolate, 5,10-methenyltetrahydrofolate, *N*^10^-formyltetrahydrofolate, thymidine-5′-phosphate (dTMP), *S*-methyl-5-thio-d-ribofuranose, *S*-methyl-5-thio-α-d-ribose 1-phosphate (1-PMTR), *S*-methyl-5-thio-d-ribulose 1-phosphate and ATP. The manual search confirmed the presence of labelled DMSP, SAM and MTA (Supplementary Data 4), which were identified using the MS2 fragmentation patterns.

### Bioinformatic analysis of methyl-group-related metabolism in algal-associated and plant-associated bacteria

Orthology clusters were used to assess the presence or absence of methyl group metabolism genes that were identified in *P. inhibens* (Supplementary Table 3) across the genomes of algal-associated and plant-associated bacteria (Table 1 and Supplementary Figs. 1 and 17). The analysis was conducted using OrthoFinder^91^, which uses MCL^92^ and FastME^93^ algorithms with default settings. Diamond BLAST results from an all-against-all search served as input data. Reference genomes were used in cases in which a specific genome was unavailable. The used genomes and their accession numbers are detailed in Supplementary Table 8.

### Reporting summary

Further information on research design is available in the Nature Portfolio Reporting Summary linked to this article.

### Supplementary information

Supplementary Information Supplementary Figs. 1–21, Tables 1–9, Data 1–4 descriptions and References.

Reporting Summary

Supplementary Data 1 *P. inhibens* feature table (genes) with results from co-cultivation RNA-sequencing run. The dataset includes bacterial gene accession numbers, functional annotations, transcript abundances (TPM-normalized read counts) and results of DESeq2 differential gene expression analysis (Fig. 1; Supplementary Figs. 1a, 3 and 4; Supplementary Tables 1 and 2).

Supplementary Data 2 *P. inhibens* feature table (genes) with results from lag phase RNA-sequencing run. The dataset includes bacterial gene accession numbers, functional annotations, transcript abundances (TPM-normalized read counts) and results of DESeq2 differential gene expression analysis (Fig. 4a, Supplementary Figs. 10–13, Supplementary Tables 6 and 7).

Supplementary Data 3 *E. huxleyi* CCMP3266 sGenome gene annotation file version 2 (GFF3 format).

Supplementary Data 4 Feature quantification obtained using Compound Discoverer. The table presents the output analysis using Compound Discoverer (v3.3), with a putative identification of metabolites.

### Source data

Source Data Fig. 2 Unprocessed OD_600_ measurements.

Source Data Fig. 3 Unprocessed OD_600_ measurements.

Source Data Table 1 Unprocessed OD_600_ measurements.

## Data Availability

All data are available in the Article or Supplementary Information. Further information and requests for resources and reagents should be directed to and will be fulfilled by the corresponding author. Plasmids generated in this study and bacteria that are not deposited in public strain collections will be made available on reasonable request. RNA-sequencing (RNA-seq) data have been deposited in the NCBI Sequence Read Archive (SRA) and are publicly available under accessions PRJNA976961 (algal–bacterial RNA-seq) and PRJNA977030 (bacterial lag phase RNA-seq). Supplementary Data 1–4 are available also via Zenodo at 10.5281/zenodo.10980548 (ref. ^94^). Bacterial methyl metabolism functional annotations were sourced from NCBI RefSeq annotations (10.1093/nar/gkv1189), partially curated GenBank submitter annotations (10.1038/ismej.2012.62), KEGG annotations (10.1093/nar/28.1.27), BioCyc (10.1093/bib/bbx085) and the unifying UniProt Archive (UniParc at 10.1093/nar/gkac1052 and InterPro database at 10.1093/nar/gkac993). Metabolomics raw data have been uploaded to the EMBL-EBI MetaboLights database with the identifier MTBLS9742 and can be accessed at https://www.ebi.ac.uk/metabolights/MTBLS9742. Metabolomics raw data can also be obtained from the corresponding author Einat.Segev@weizmann.ac.il. Source data are provided with this paper.
