# Supplementary material for: Algal methylated compounds shorten the lag phase of Phaeobacter inhibens bacteria
Source: Nat Microbiol. 2024 Jul 5;9(8):2006–21. doi: 10.1038/s41564-024-01742-6 (PMC11306105; doi:10.1038/s41564-024-01742-6)
Supplement: Supplementary file 1 — Supplementary Figs. 1–21, Tables 1–9, Data 1–4 descriptions and References. [file 41564_2024_1742_MOESM1_ESM.pdf]

nature microbiology

Supplementary information

https://doi.org/10.1038/s41564-024-01742-6

Algal methylated compounds shorten the lag phase of *Phaeobacter inhibens* bacteria

In the format provided by the authors and unedited

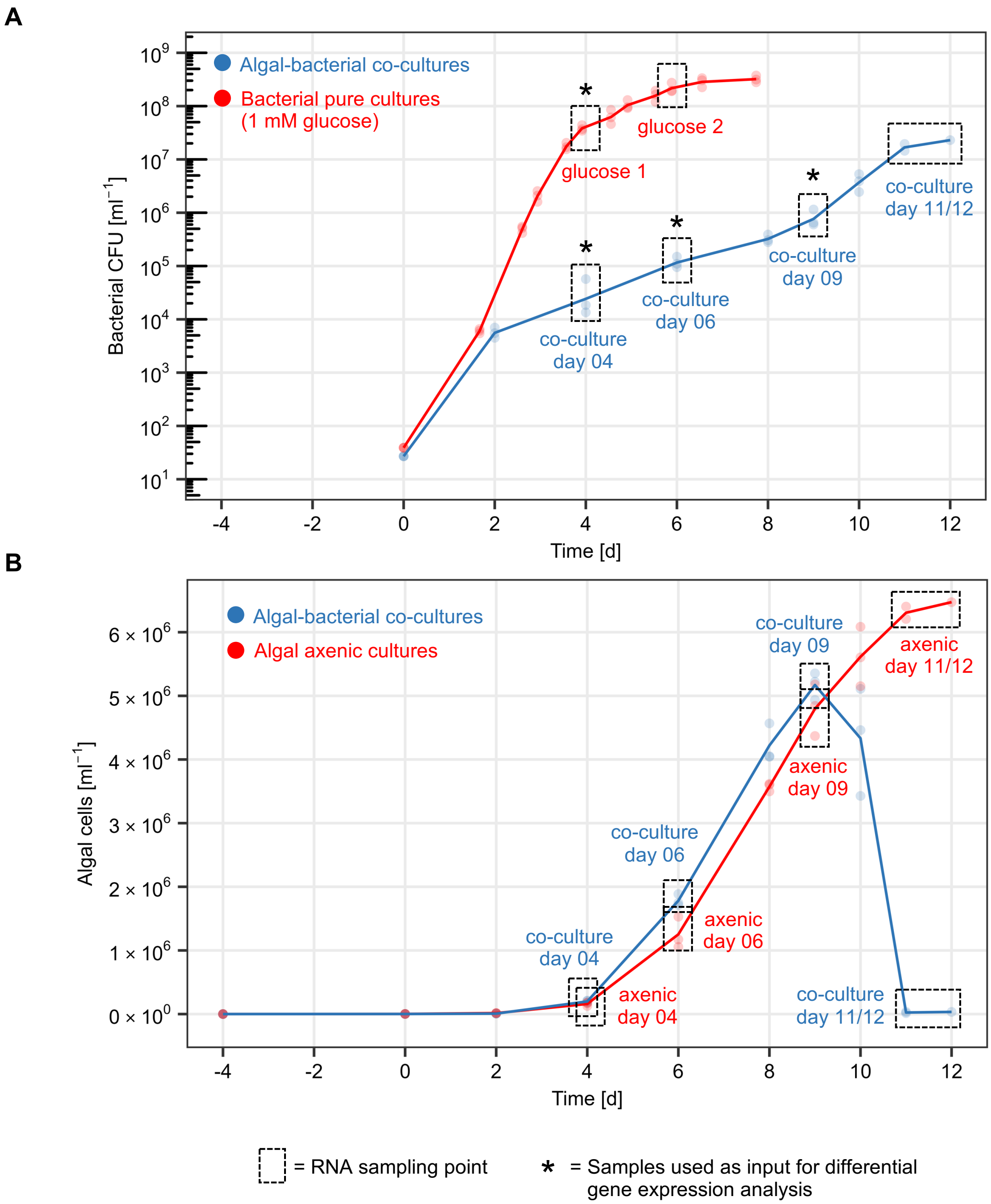

Fig. S1: RNA sampling points of microbial cultures. (**A**) RNA was sampled from the late exponential and stationary phases of *P. inhibens* bacteria grown in pure cultures with glucose (red; samples designated glucose 1 and 2, respectively), as well as from the different interaction phases of *P. inhibens* in co-cultures with *E. huxleyi* algae (blue; samples designated co-cultures day 04 to day 11/12). All RNA samples (dashed box) were subjected to co-culture RNA-sequencing (see *Method Details*). Since exponentially growing bacteria in co-cultures are under constant nutrient limitation, they were compared with the late-exponential growth phase in pure cultures, in which nutrients become limiting. As mentioned in the text, the limitations of such a comparison should be acknowledged, yet this analysis can provide valuable observations and serve to generate hypotheses for further exploration. Samples marked with asterisk were included in the differential gene expression analysis to compare exponentially growing bacterial pure cultures (glucose 1) with bacteria in co-cultures with algae (co-culture day 04, 06 and 09). Three flasks were sampled per time point and condition (biological triplicate), and each dot represents the bacterial CFU number (determined as technical duplicate). (**B**) Bacteria stimulate algal growth during the early interaction phase and promote algal death during the late interaction phase (blue: algal growth in co-cultures with bacteria, corresponding to panel A; red: algal growth in axenic cultures, i.e. without bacteria). RNA samples of algae without bacteria (axenic day 04 – 11/12) were not analyzed for differential gene expression in this study. Three flasks were sampled per time point and condition. Each dot represents the mean of algal flow cytometry cell counts, determined as technical triplicate for every sampled flask. The lines in (**A**) and (**B**) depict the mean of the plotted dots for each condition.

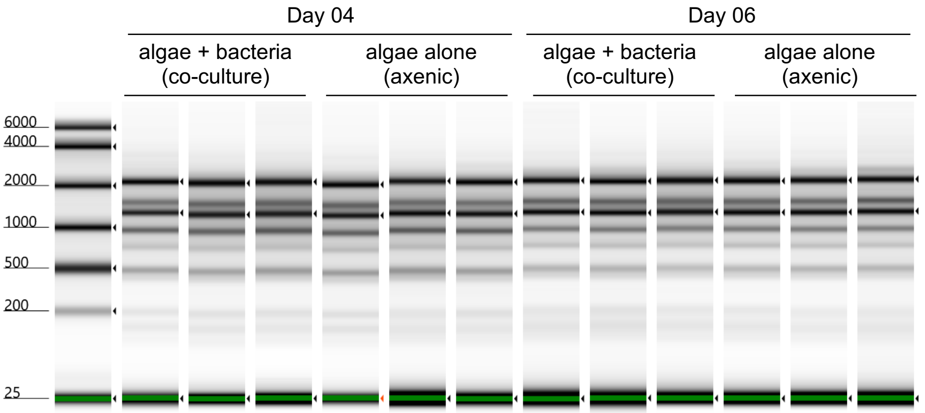

Sample ID | replicate	co-culture day 04 | rep 1	co-culture day 04 | rep 2	co-culture day 04 | rep 3	axenic day 04 | rep 1	axenic day 04 | rep 2	axenic day 04 | rep 3	co-culture day 06 | rep 1	co-culture day 06 | rep 2	co-culture day 06 | rep 3	axenic day 06 | rep 1	axenic day 06 | rep 2	axenic day 06 | rep 3
Qubit [ng/ul]	146.4	116	105.4	155.8	68	108.8	369	427	441	418	342	342
Tapestation [ng/ul]	105.4	108.4	94.6	146.8	61	93.4	343	388	407	385	308	333
Tapestation (RIN)	6.7	6.3	6	6.4	6.4	6.6	7.2	7	6.9	7.2	7.3	7.3

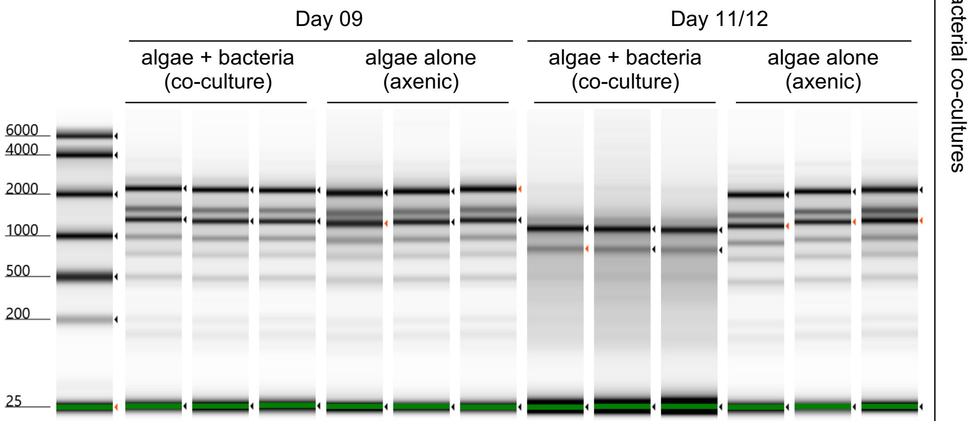

Sample ID | replicate	co-culture day 09 | rep 1	co-culture day 09 | rep 2	co-culture day 09 | rep 3	axenic day 09 | rep 1	axenic day 09 | rep 2	axenic day 09 | rep 3	co-culture day 11/12 | rep 1	co-culture day 11/12 | rep 2	co-culture day 11/12 | rep 3	axenic day 11/12 | rep 1	axenic day 11/12 | rep 1	axenic day 11/12 | rep 1
Qubit [ng/ul]	756	622	608	664	688	633	103.5	99.7	70.8	684	775	667
Tapestation [ng/ul]	595	460	462	563	600	570	57.8	61.8	40.6	506	592	520
Tapestation (RIN)	7.6	7.6	7.8	6.9	7.5	7.5	4.6	4.4	4.2	7.5	7.6	7.1

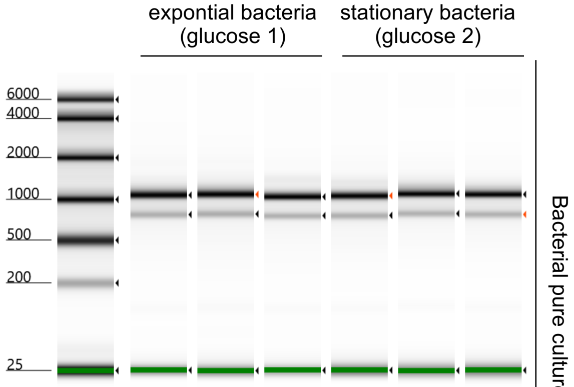

Sample ID | replicate	glucose 1 | rep 1	glucose 1 | rep 2	glucose 1 | rep 3	glucose 2 | rep 1	glucose 2 | rep 2	glucose 2 | rep 3
Qubit [ng/ul]	334	274	360	469	511	427
Tapestation [ng/ul]	232	214	256	400	427	362
Tapestation (RIN)	8.6	8.4	8.6	8.5	8.8	8.7

Fig. S2: Quality control and quantification of RNA samples extracted for co-culture RNA-sequencing analysis. Tapestation intensities were scaled to samples. Arrows indicate ribosomal subunits. RIN—RNA integrity numbers as determined by the Tapestation software.

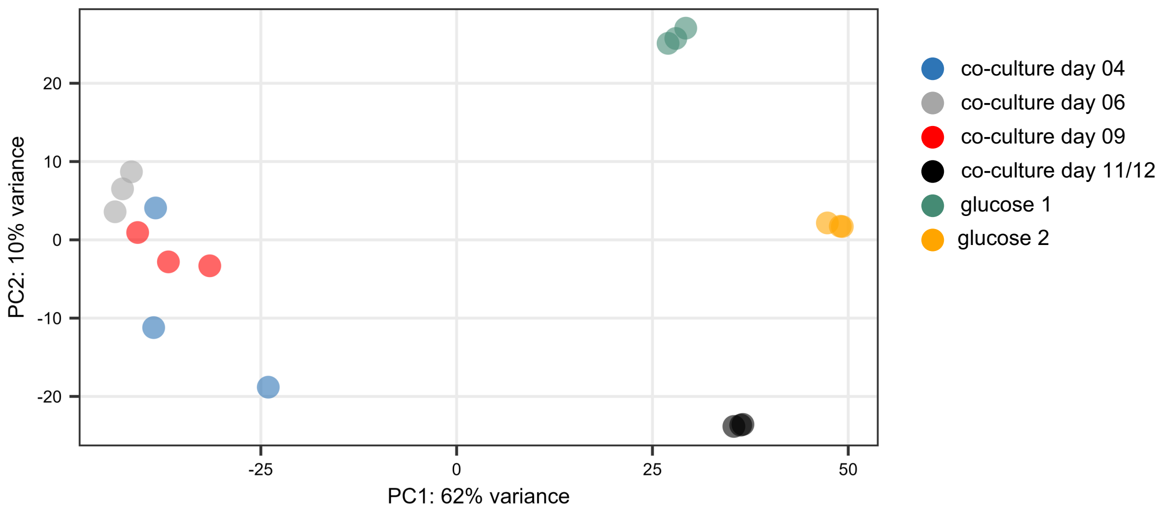

Fig. S3: Characterization of bacterial gene transcription profiles in the presence and absence of algae. Bacterial transcription profiles were similar in the presence of live algae (co-culture day 04, 06 and 09), but shifted when algal death was evident (co-culture day 11/12), or in the absence of algae (glucose 1 and 2). Each condition was analyzed as biological triplicate. Based on the close clustering of co-culture samples with live algae (co-culture day 04, 06 and 09), these nine samples were treated as replicates and compared to samples of exponentially growing bacteria on glucose (glucose 1) for differential gene expression analysis. The PCA plot was generated using the DESeq2::plotPCA function with default options (ntop=500; top 500 variable genes) and DESeq2::rlog transformed read counts as input.

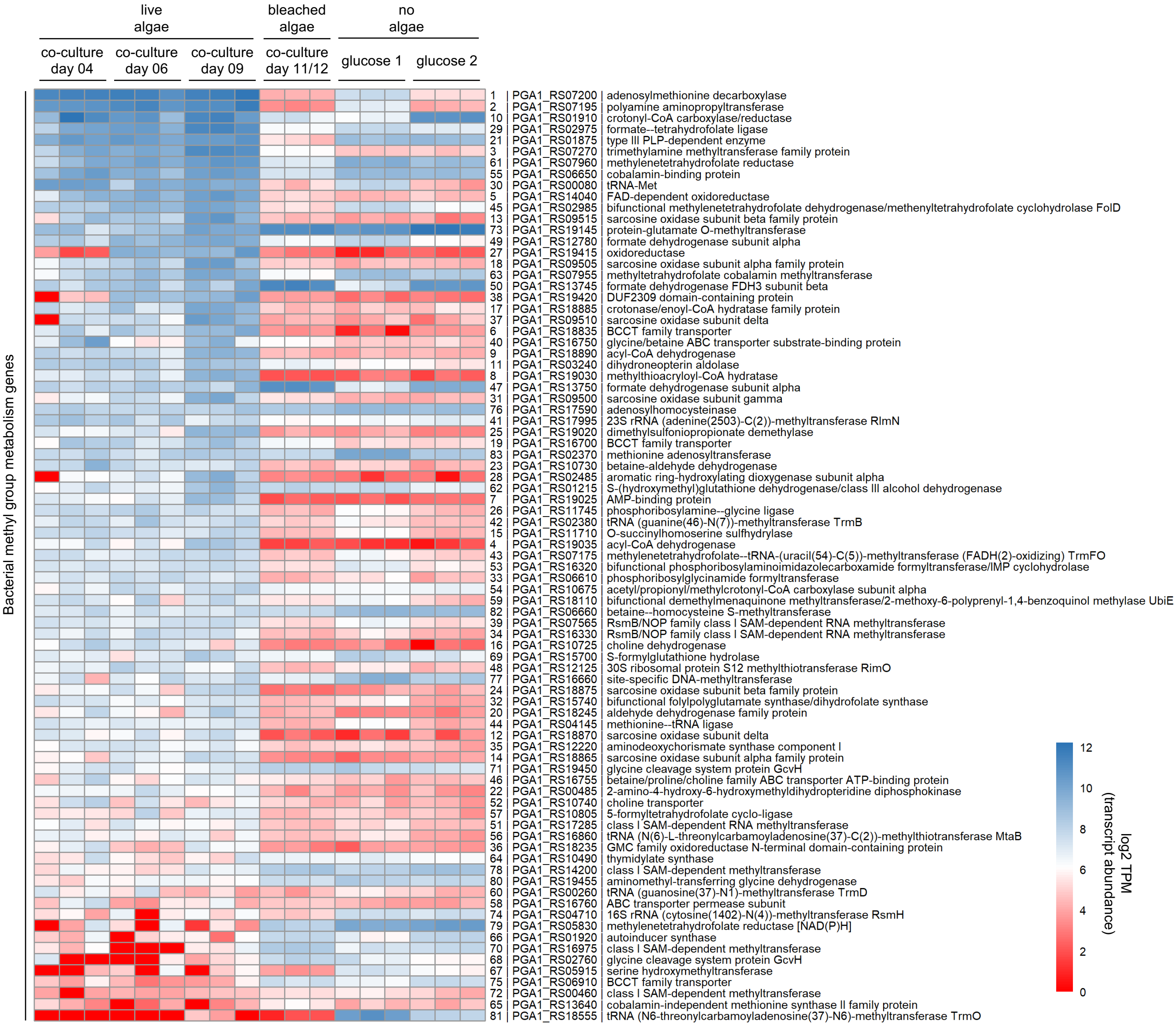

Fig. S4: Transcript abundances of 83 manually curated bacterial methyl group metabolism genes in the presence and absence of algae. Transcript abundances were similar in co-cultures with live algae (co-culture day 04, 06 and 09), but shifted when algal death was evident (co-culture day 11/12), or in the absence of algae (glucose 1 and 2). Rows were sorted from highest to lowest mean transcript abundances in co-culture samples with live algae (co-culture day 04, 06 and 09). Transcript abundances are presented as log2 TPM (transcripts per kilobase million) normalized paired-end read counts. Row names include gene numbers (as appears throughout the current study), followed by the RefSeq gene locus tag accession and the RefSeq functional annotation.

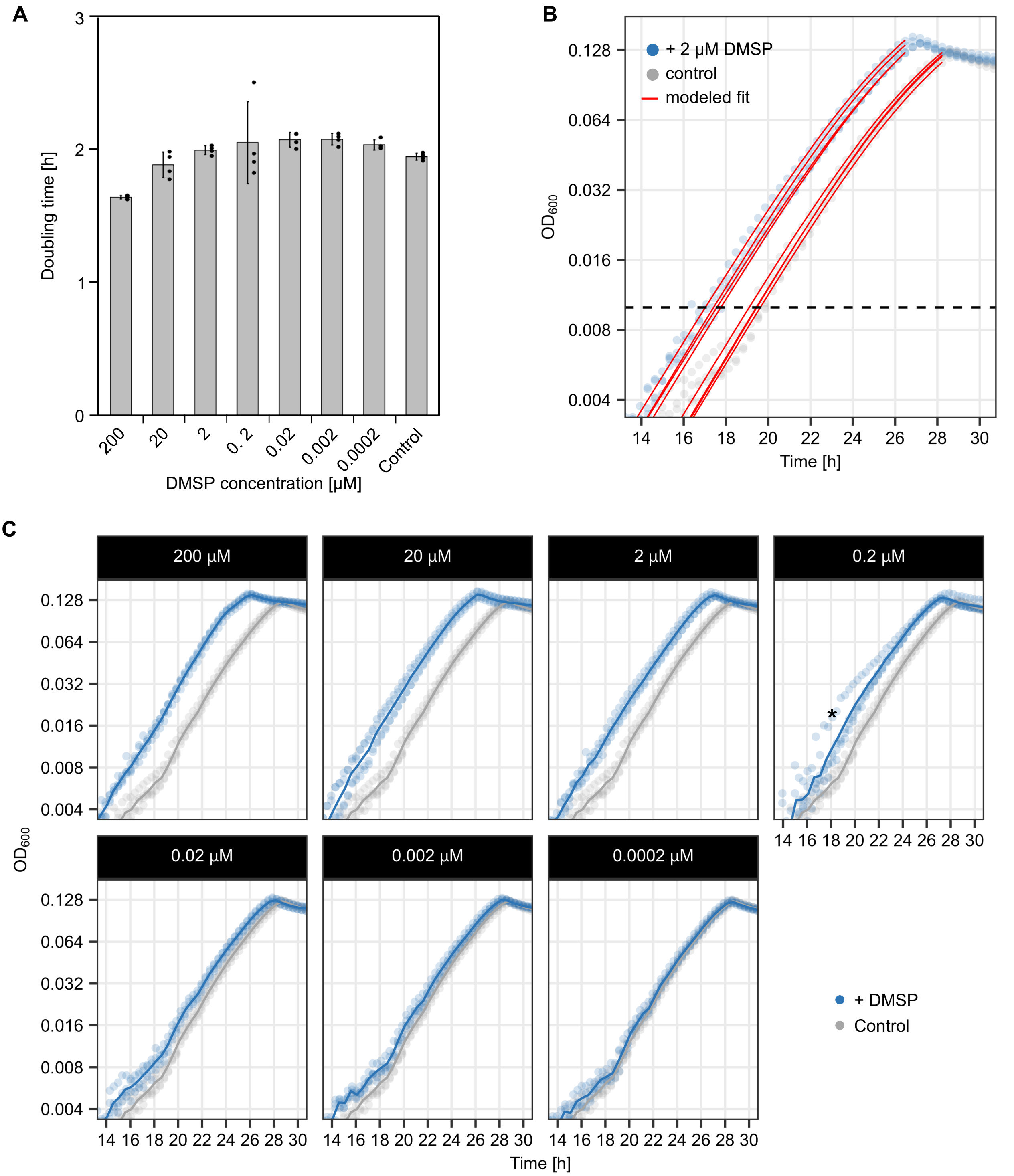

Fig. S5: Bacterial growth dynamics in response to DMSP. (**A**) Doubling times of bacterial pure cultures supplemented with different DMSP concentrations, calculated from the modeled fit. Error bars indicate standard deviations of the mean for four biological replicates, represented by dots. (**B**) Lag times were determined for individual replicates by fitting an exponential growth function (red lines) and determining the time at which each replicate reached OD_{600} 0.01 (dashed line). (**C**) Growth curves of bacterial cultures supplemented with different DMSP concentrations (indicated above the panels). The OD_{600} measurements were used to calculate lag times (Fig. 2C) and doubling times (panel **B**). The asterisk marks an outlier replicate that was not included in the calculations. Dots represent measurements for individual cultures and lines depict a smoothed average of four biological replicates.

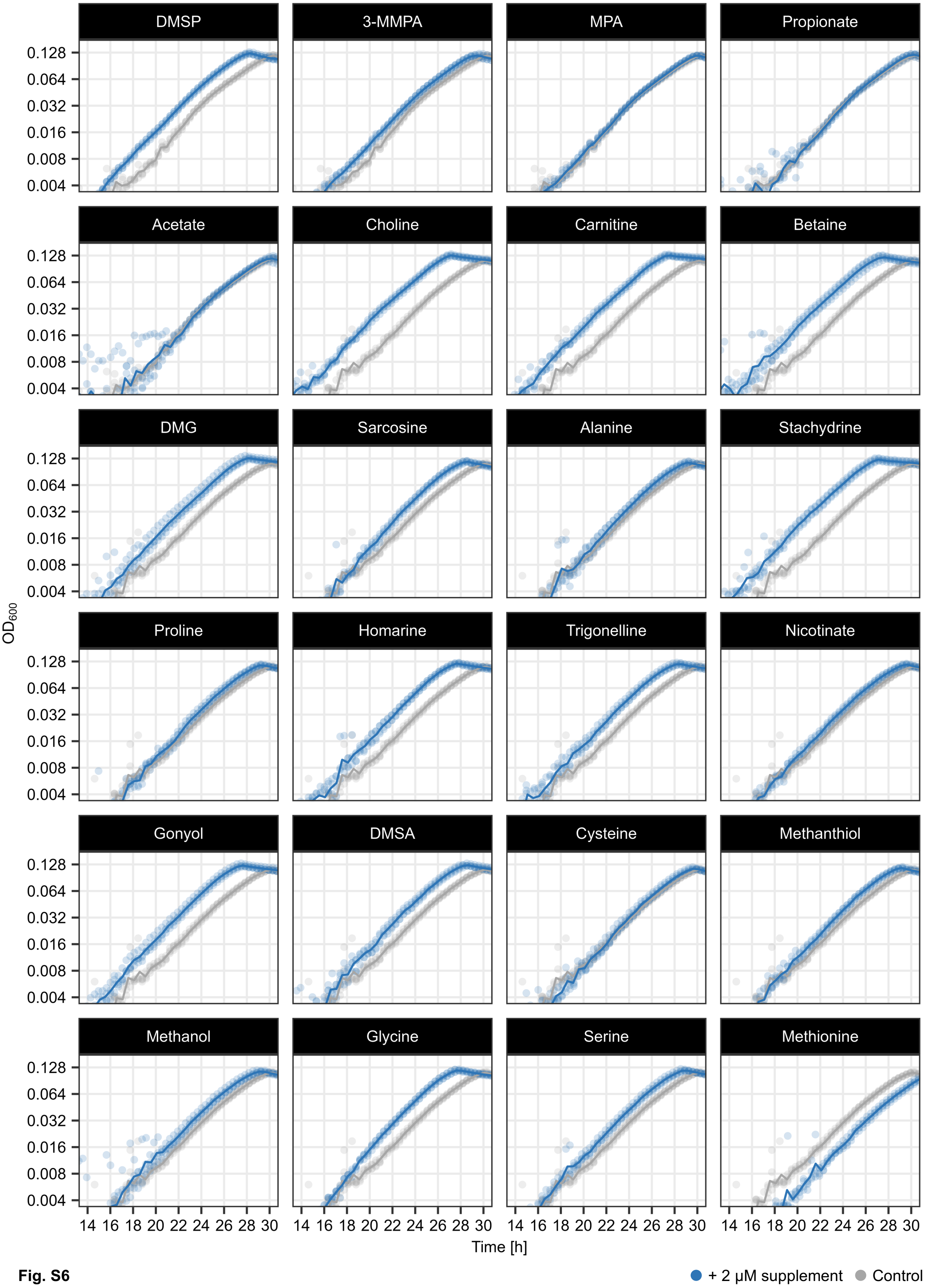
DMSP
3-MMPA
MPA
Propionate
Acetate
Choline
Carnitine
Betaine
DMG
Sarcosine
Alanine
Stachydrine
Proline
Homarine
Trigonelline
Nicotinate
Gonyol
DMSA
Cysteine
Methanthiol
Methanol
Glycine
Serine
Methionine
0.128
0.064
0.032
0.016
0.008
0.004
OD_{600}
14 16 18 20 22 24 26 28 30
Time [h]
+ 2 µM supplement
Control

Fig. S6

Fig. S6: Bacterial growth dynamics in response to different supplements. Growth curves of supplemented cultures (blue) were compared to growth curves of control cultures (grey). Experiments were conducted with four biological replicates. Growth dynamics in response to DMSP analogues (DMSP, 3-MMPA, MPA, propionate, acetate) and other methylated compounds (choline, carnitine, betaine, DMG, sarcosine, alanine, stachydrine, proline, homarine, trigonelline, nicotinate, gonyol, DMSA, cysteine, methanthiol, methanol, glycine, serine, methionine) were measured in artificial seawater medium (ASW_b) with 1 mM glucose and 2 µM of the respective supplement. The OD_{600} measurements were used to calculate lag times (Fig. 3A-B) and doubling times (Fig. S7). Dots represent measurements for individual cultures and lines depict a smoothed average of four biological replicates.

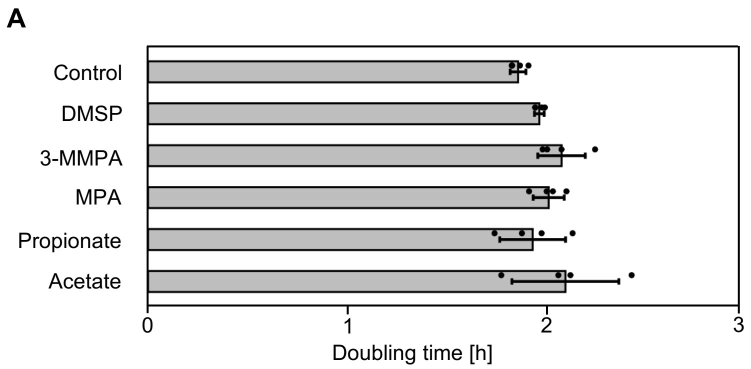

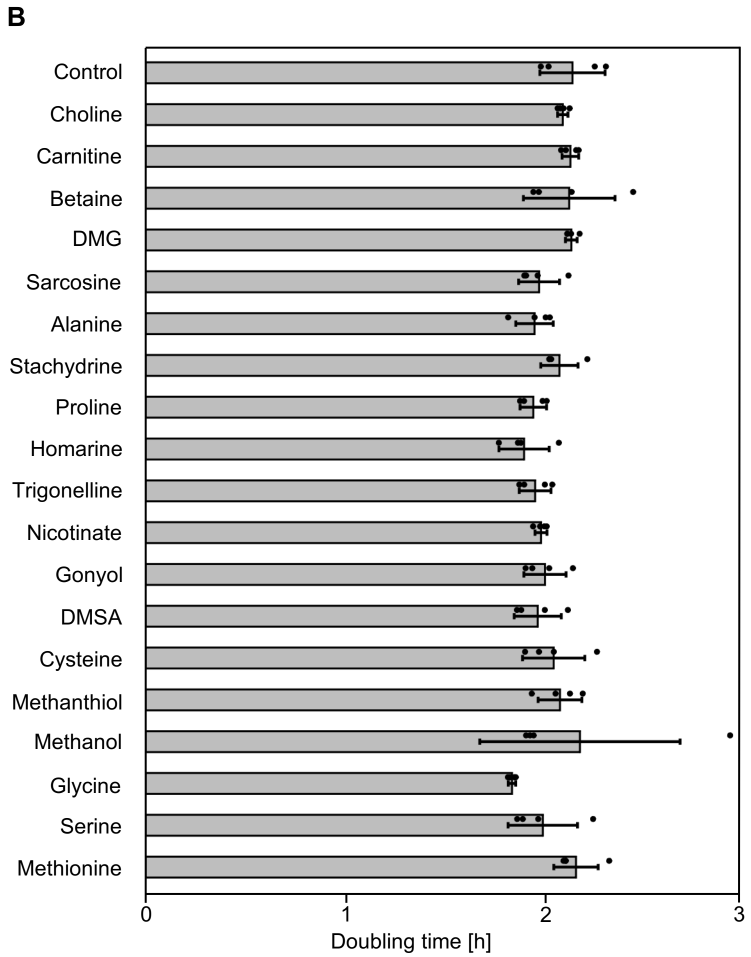

Fig. S7: Doubling times of *P. inhibens* bacteria grown in the presence of different supplements. Bacterial growth dynamics were monitored in response to (**A**) analogues of DMSP and (**B**) other supplements, mainly methylated compounds. Cultivations were conducted with four biological replicates in artificial seawater medium (ASW_b) with 1 mM glucose and 2 μM of the respective supplement. Error bars indicate standard deviations of the mean for four biological replicates, represented by dots. Differences between control cultures and supplemented treatment cultures were not significant using a two-way ANOVA test.

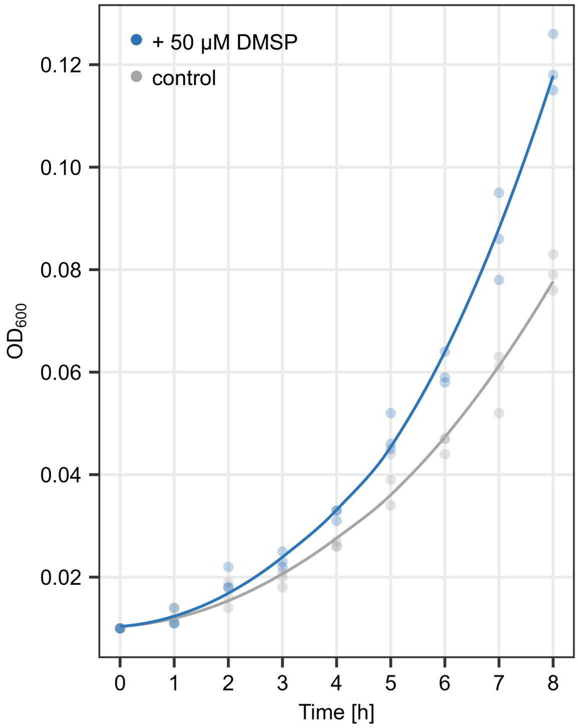

Fig. S8: Growth dynamics of *P. inhibens* bacteria in large volume cultures initiated with a denser inoculum and more DMSP. Cultures were inoculated with OD_{600} = OD 0.01 in 60 mL ASW_b medium (1 mM glucose) and supplemented with 50 µM DMSP. A comparatively high starting OD together with more DMSP yielded sufficient biomass for lag phase RNA-sequencing and [^{13}C-methyl] DMSP LC-MS analysis. Dots represent measurements for individual cultures and lines depict a smoothed average of three biological replicates.

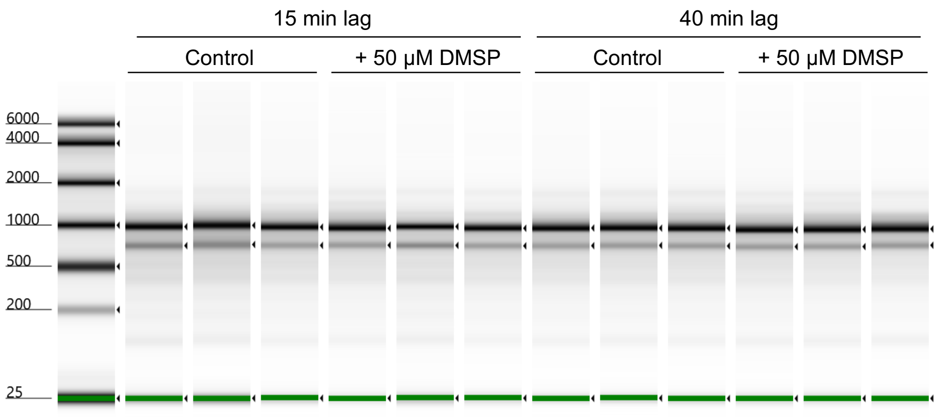

Sample ID | replicate	Control (15 min lag) | rep 1	Control (15 min lag) | rep 1	Control (15 min lag) | rep 1	DMSP (15 min lag) | rep 1	DMSP (15 min lag) | rep 2	DMSP (15 min lag) | rep 3	Control (40 min lag) | rep 1	Control (40 min lag) | rep 2	Control (40 min lag) | rep 3	DMSP (40 min lag) | rep 1	DMSP (40 min lag) | rep 2	DMSP (40 min lag) | rep 3
Qubit [ng/ul]	245	195	346	444	240	372	361	337	335	434	460	478
Tapestation [ng/ul]	171	155	272	412	199	281	335	297	310	309	303	353
Tapestation (RIN)	6.1	5.9	6.0	6.3	6.5	6.1	6.1	6.2	6.3	6.4	6.6	6.8

Fig. S9: Quality control and quantification of RNA samples extracted from *P. inhibens* bacterial pure cultures during the lag phase. Tapestation intensities were scaled to samples. Arrows indicate ribosomal subunits. RIN—RNA integrity numbers as determined by the Tapestation software.

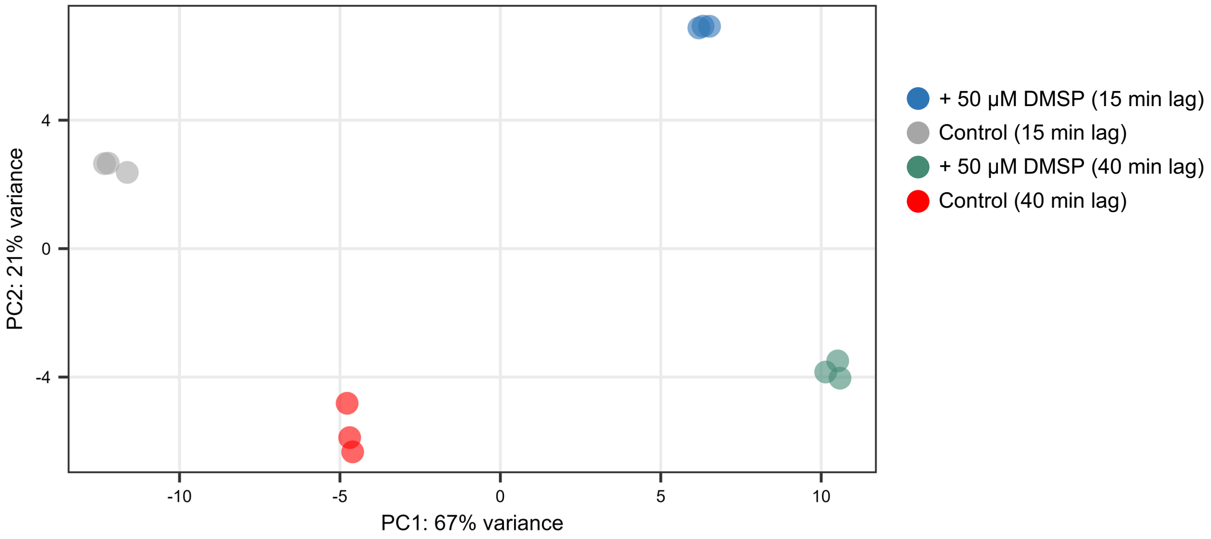

Fig. S10: Characterization of bacterial gene transcription profiles during the lag phase in cultures supplemented with DMSP compared to control cultures. Exposure to 50 µM DMSP altered gene transcription profiles during the lag phase, as evident by a pronounced separation of supplemented cultures and control cultures on the PC1 axis. RNA was sampled 15 min and 40 min after transfer of stationary phase bacteria into fresh medium containing 1 mM glucose. Cultures were initiated with a density of OD_{600} = 0.01 (Fig. S8). The PCA plot was generated using the DESeq2::plotPCA function with default options (ntop=500; top 500 variable genes) and DESeq2::rlog transformed read counts as input.

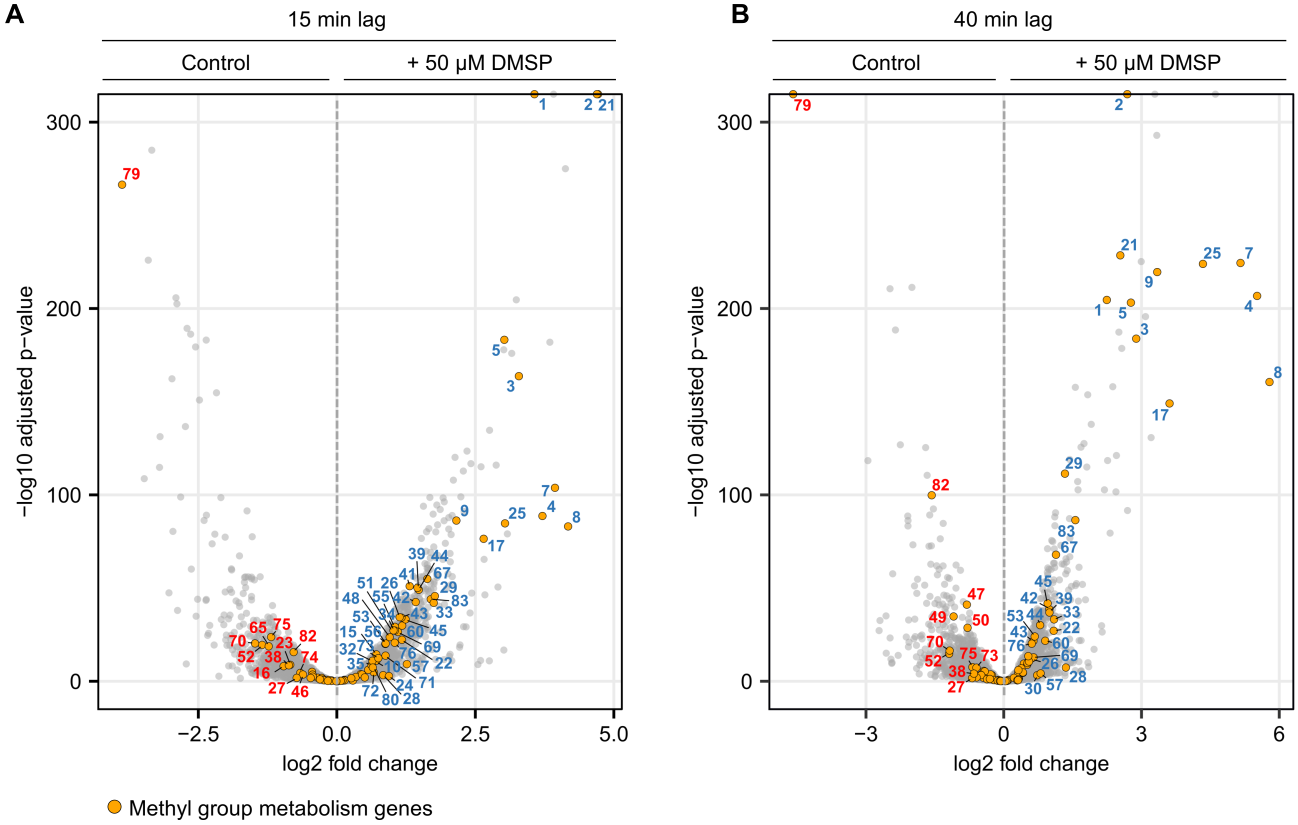

Fig. S11: Methyl group metabolism genes were upregulated in DMSP-supplemented lag phase bacteria compared to control cultures. The transcriptional response was measured (**A**) 15 min and (**B**) 40 min after initiation of the cultures. Orange dots depict 83 methyl group metabolism genes of interest (see table S3 for gene accessions and functional annotations). Gene numbers in blue and red indicate genes that were significantly upregulated or downregulated in the presence of DMSP compared to control cultures, respectively (thresholds for coloring: adjusted *p*-value < 0.05; log2 fold change < -0.585 and > +0.585). Overall, 43 and 29 methyl group metabolism genes were upregulated, while 12 and 12 genes were downregulated after 15 min and 40 min, respectively. The most upregulated genes were involved in DMSP catabolism (gene 4, 7, 8, 9, 17, 25) and spermidine synthesis (gene 1, 2 and 21), while the most downregulated gene was associated with the oxidation of methyl groups (gene 79).

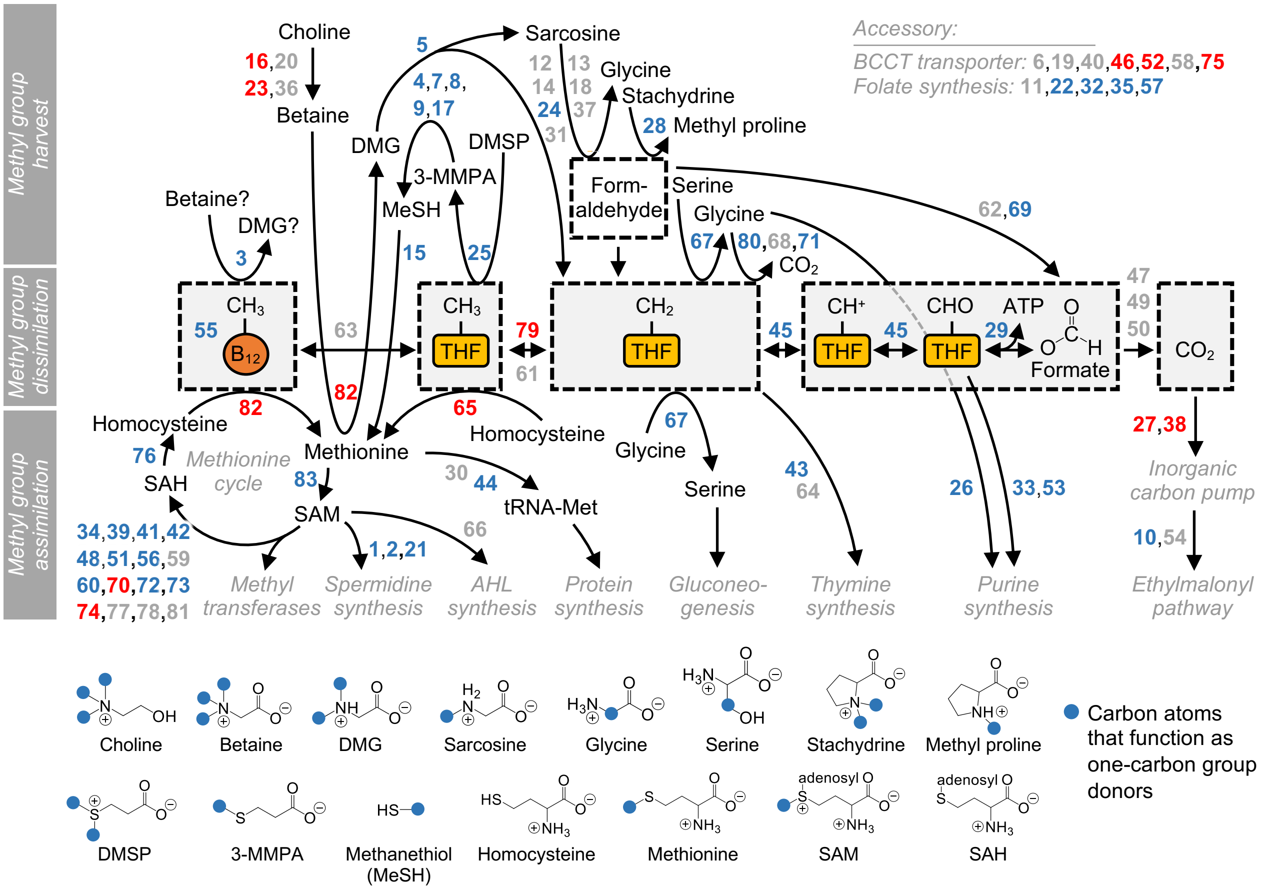

Fig. S12: Transcriptional response of *P. inhibens* bacteria towards DMSP during the lag phase. The presented data were generated by harvesting DMSP-supplemented and non-supplemented cultures 15 min after initiation, using biological triplicates (based on data presented in figs. S8-S11, tables S3, S6-S7 and data S2). Numbers in blue, grey and red indicate genes that were upregulated (43 genes), unchanged (28 genes) or downregulated (12 genes) in cultures supplemented with DMSP compared to control cultures, respectively (thresholds for coloring: adjusted *p*-value < 0.05; log2 fold change < -0.585 and > +0.585).

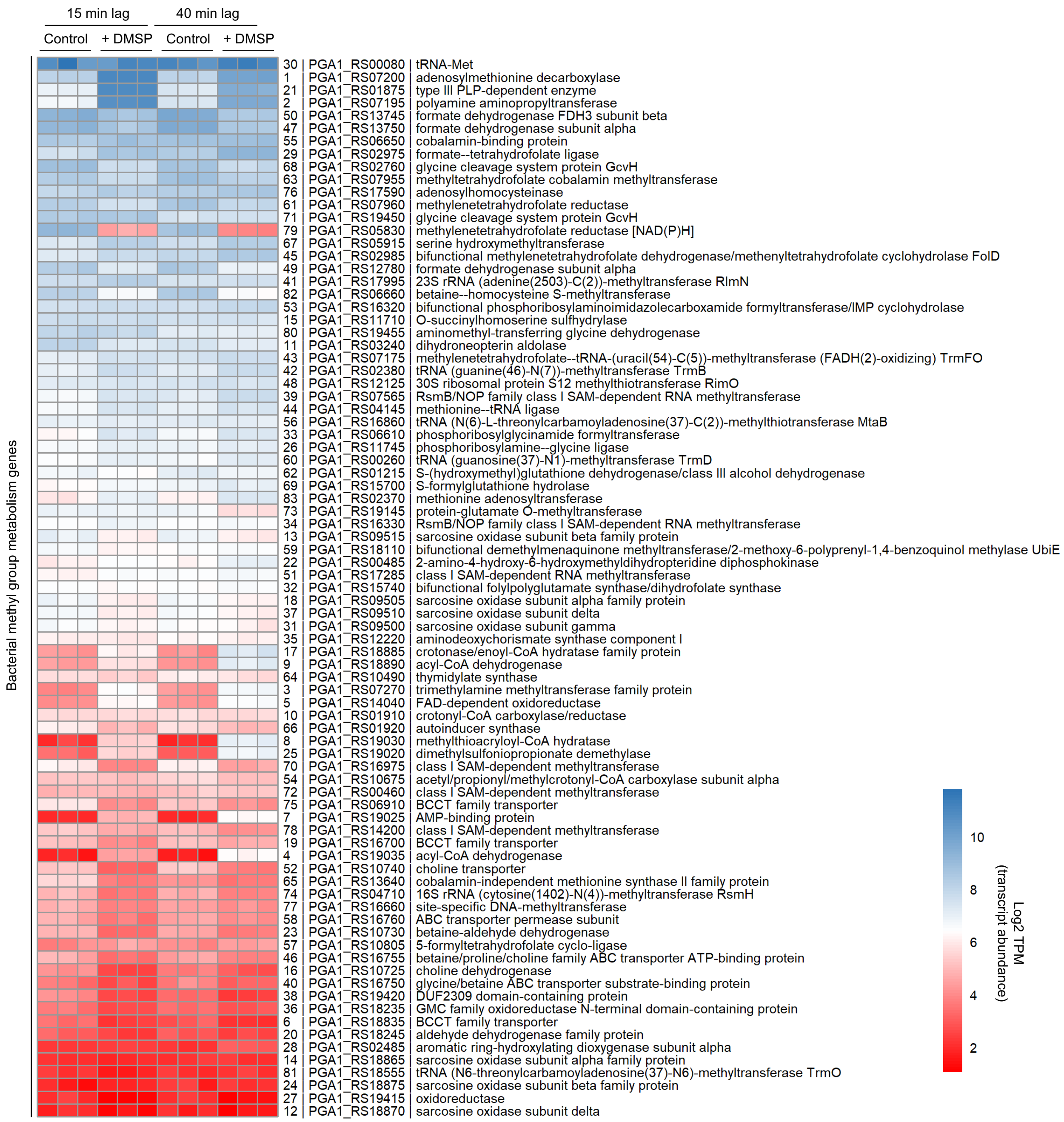

Fig. S13: Transcript abundances of methyl group metabolism genes during the lag phase of *P. inhibens* bacteria supplemented with 50 µM DMSP compared to control cultures. Rows were sorted from highest to lowest mean transcript abundances over all samples. Transcript abundances are presented as log2 TPM (transcripts per kilobase million) normalized paired- end read counts. Row names include gene numbers as appear throughout the current study, followed by the RefSeq gene locus tag accession and the RefSeq functional annotations. Based on data presented in figs. S8-S12, tables S3, S6-S7 and data S2.

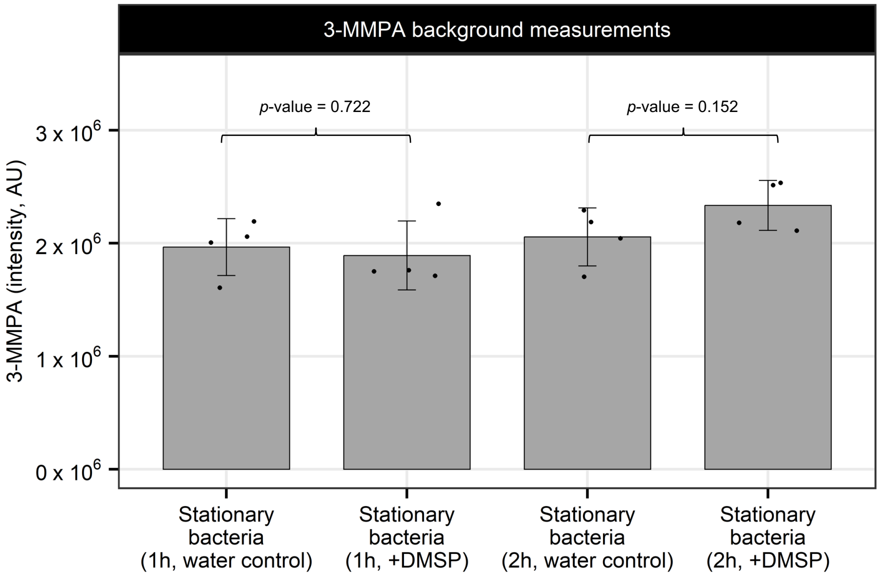

Fig. S14: Measurements of 3-MMPA in stationary phase bacteria, prior to generating crude extracts for enzymatic reactions. Samples of stationary bacteria were taken after incubating bacteria for either 1 or 2 hours with 2 µM DMSP (or water as control). For comparison with Fig. 4C, results were normalized to protein concentrations. Measured 3-MMPA levels were similar across all samples. Bars represent the average of four biological replicates with error bars showing the standard deviation. Two-tailed Student's t-Test was used to calculate *p*-values.

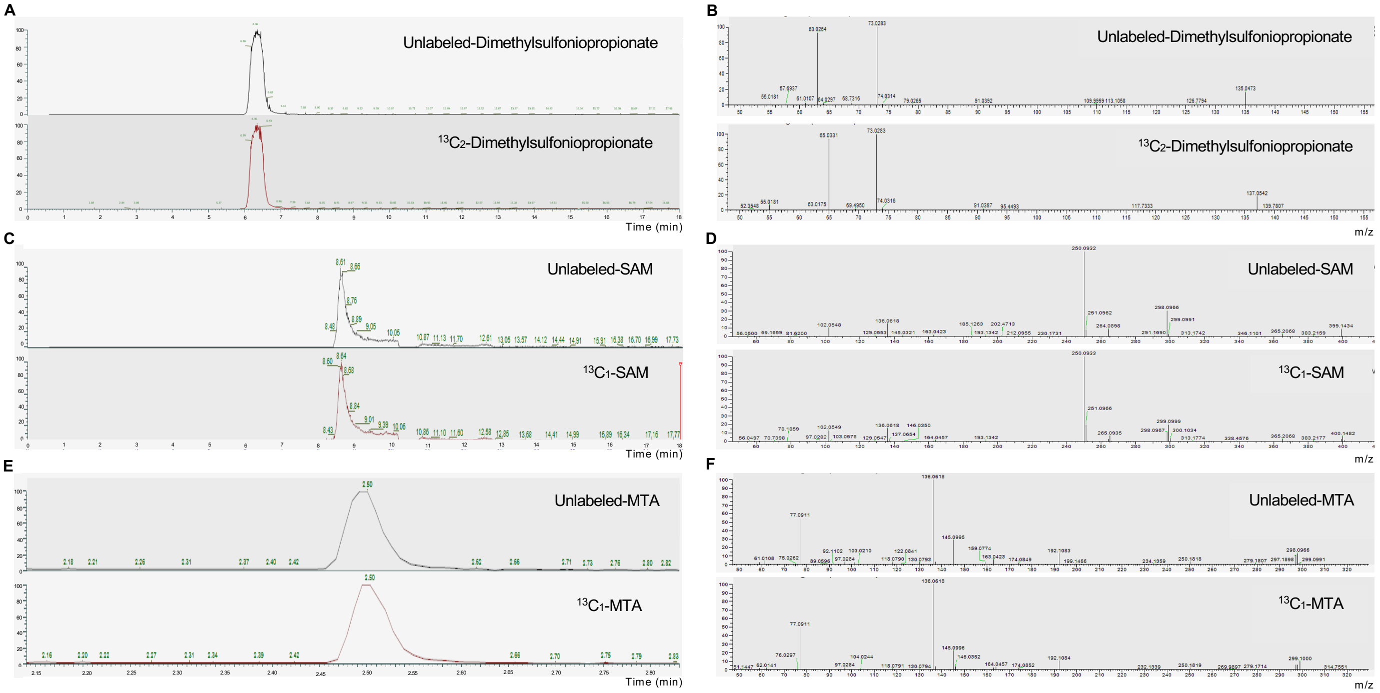

Fig. S15: Representative LC-MS/MS chromatograms and MS/MS spectra of detected unlabeled and labeled compounds. (**A-B**) Dimethylsulfoniopropionate (DMSP) and $^{13}C_2$-DMSP. (**C-D**) unlabeled-*S*-Adenosylmethionine (SAM) and $^{13}C_1$-SAM. (**E-F**) unlabeled-5'-*S*-Methyl-5'-thioadenosine (MTA) and $^{13}C_1$-MTA. The observed mass shifts are associated with the ^{13}C isotope.

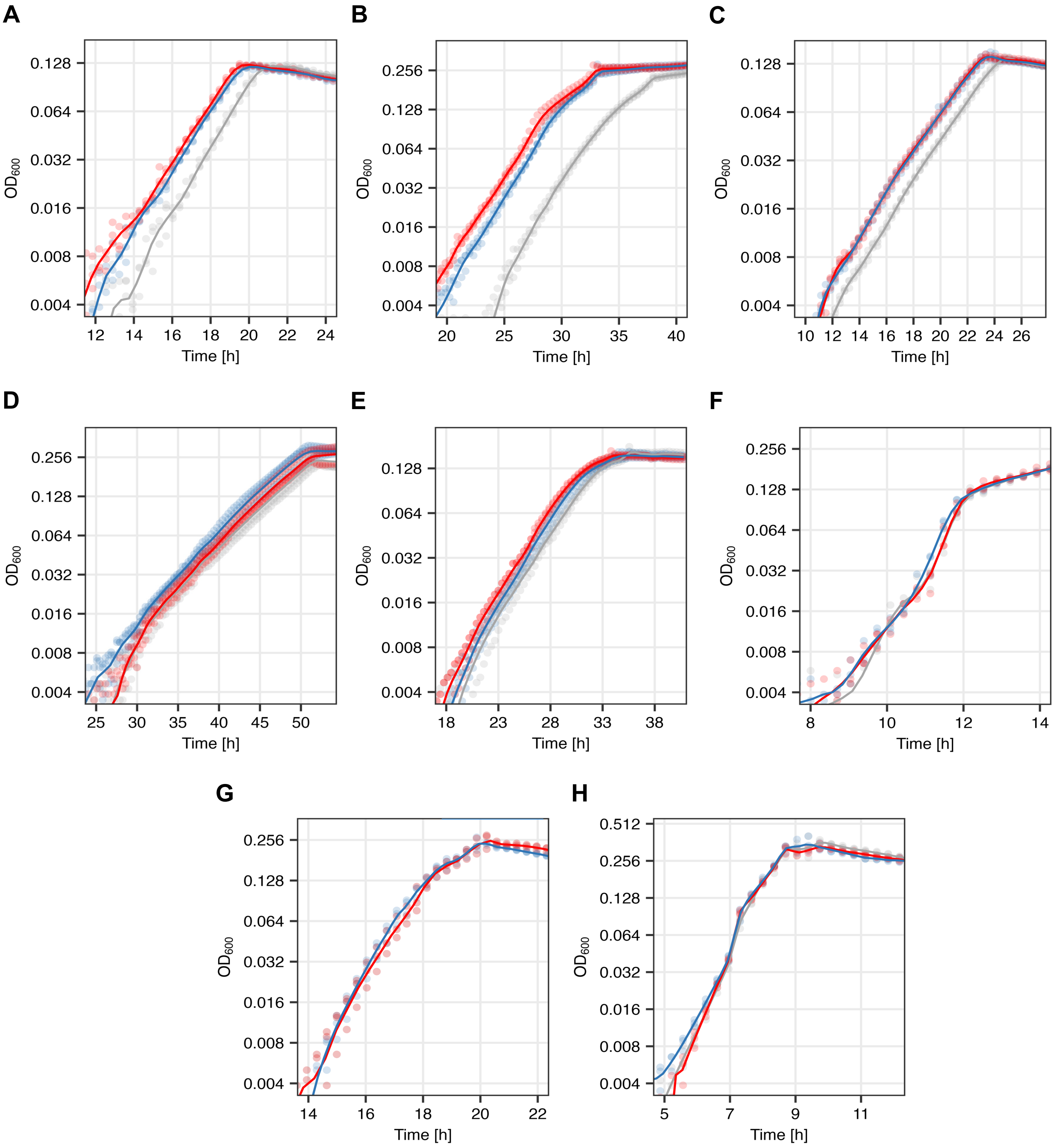

Fig. S16: Marine bacteria (**A**) *Sulfitobacter pontiacus* iR4, (**B**) *Labrenzia* sp. DT40, (**C**) *Vibrio* sp. DT22, (**D**) *Phaeobacter* sp. DT104, (**E**) *Pseudoalteromonas* sp. DT35, (**F**) *Marinomonas* sp. DT74, (**G**) *Labrenzia* sp. DT112 and (**H**) *Vibrio* sp. DT18, exhibit various lag reduction capabilities in response to 2 µM DMSP (blue) or betaine (red) when compared to control cultures (grey). Optical Density (OD_{600}) measurements are presented in logarithmic scale. Dots represent measurements for individual cultures and lines depict a smoothed average of three replicates.

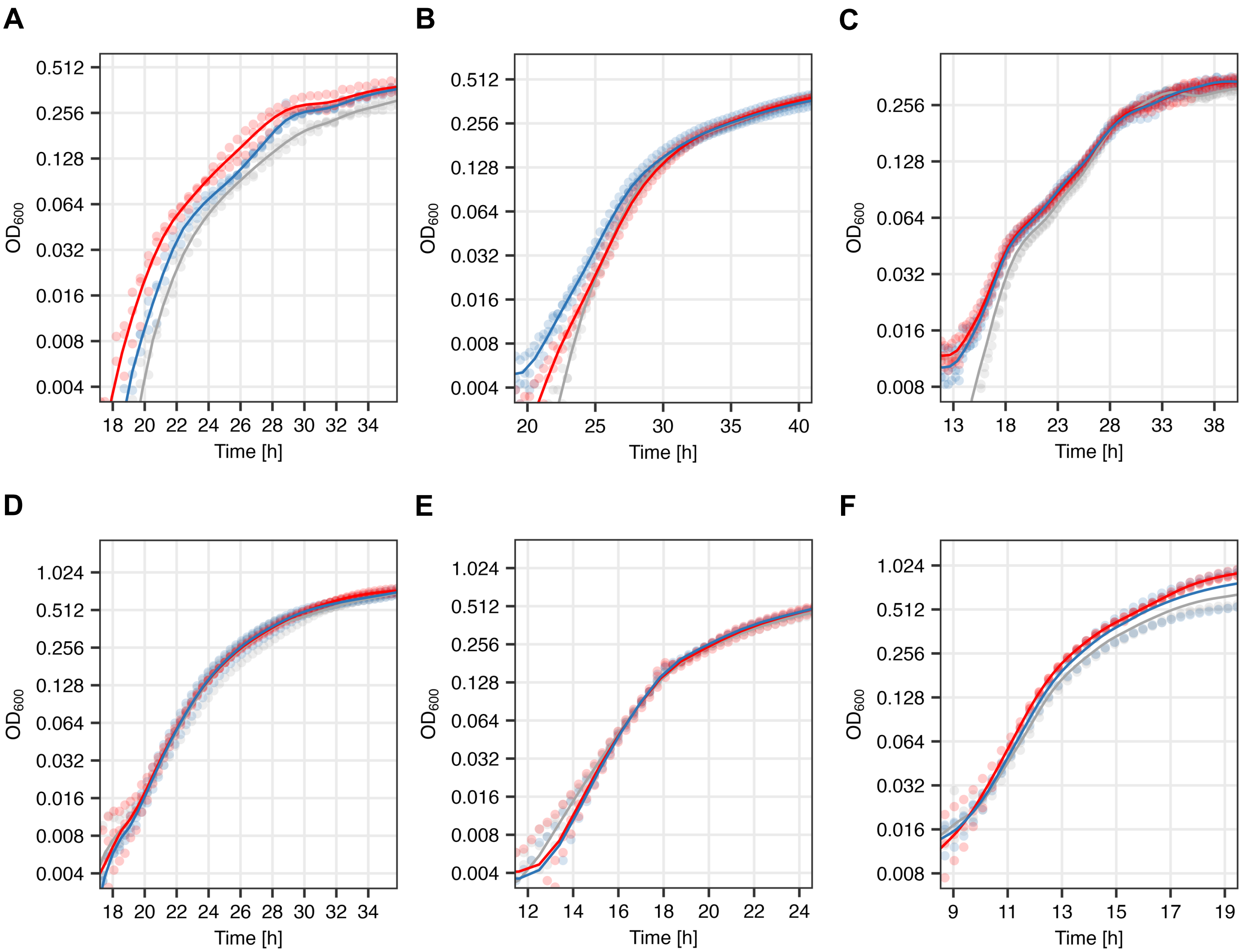

Fig. S17: Plant-associated bacteria (**A**) *Bacillus subtilis* 168, (**B**) *Pseudomonas koreensis* PK, (**C**) *Pseudomonas aeruginosa* PAO1, (**D**) *Ewingella americana* EA, (**E**) *Lelliottia amnigena* LA, and (**F**) *Enterobacter aerogenes* Ea, exhibit various lag reduction capabilities in response to 2 µM DMSP (blue) or 2 µM betaine (red) when compared to control cultures (grey). Optical Density (OD_{600}) measurements are presented in logarithmic scale. Dots represent measurements for individual cultures and lines depict a smoothed average of three replicates.

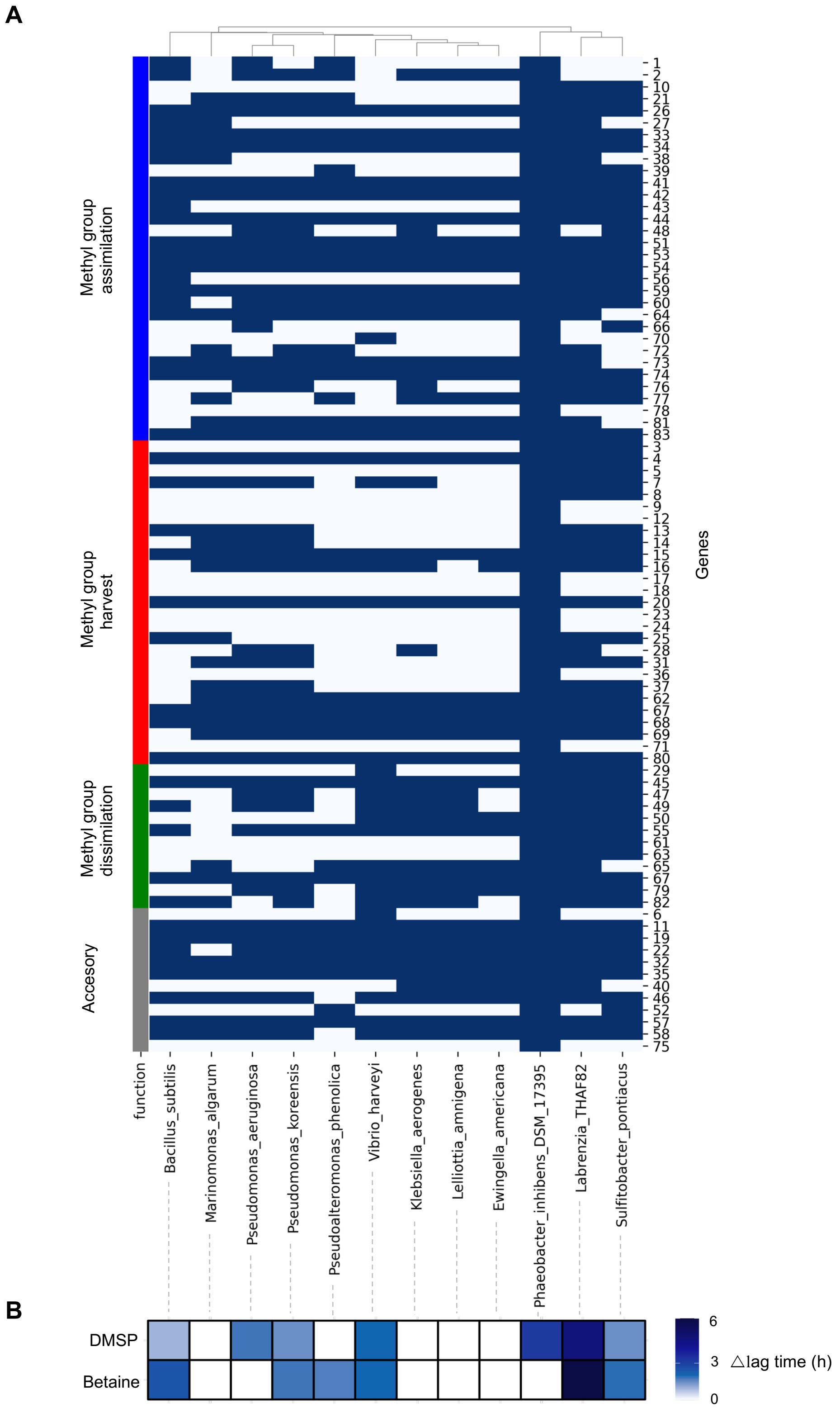

Fig. S18: Methyl group-related metabolism in algal-associated and plant-associated bacteria. (**A**) Orthology clusters indicate the presence (blue) or absence (white) of methyl group metabolism genes that were identified in *P. inhibens* (genes numbers correspond to table S3) among the genomes of the tested algal-associated and plant-associated bacteria (Table 1, Extended Data figs. S16 and S17), as determined by OrthoFinder[1]. OrthoFinder employs MCL[2] and FastME[3] to delineate orthogroups, with default settings retained. Diamond[4] blast results from an all-against-all search were utilized as input. In cases where a genome was unavailable, a reference genome was used (see table S8 for list of genomes with NCBI accession numbers). (**B**) The heatmap shows the diverse lag phase reduction capabilities (in hours) in response to DMSP and betaine among the tested bacteria (Table 1, figs. S16 and S17).

A

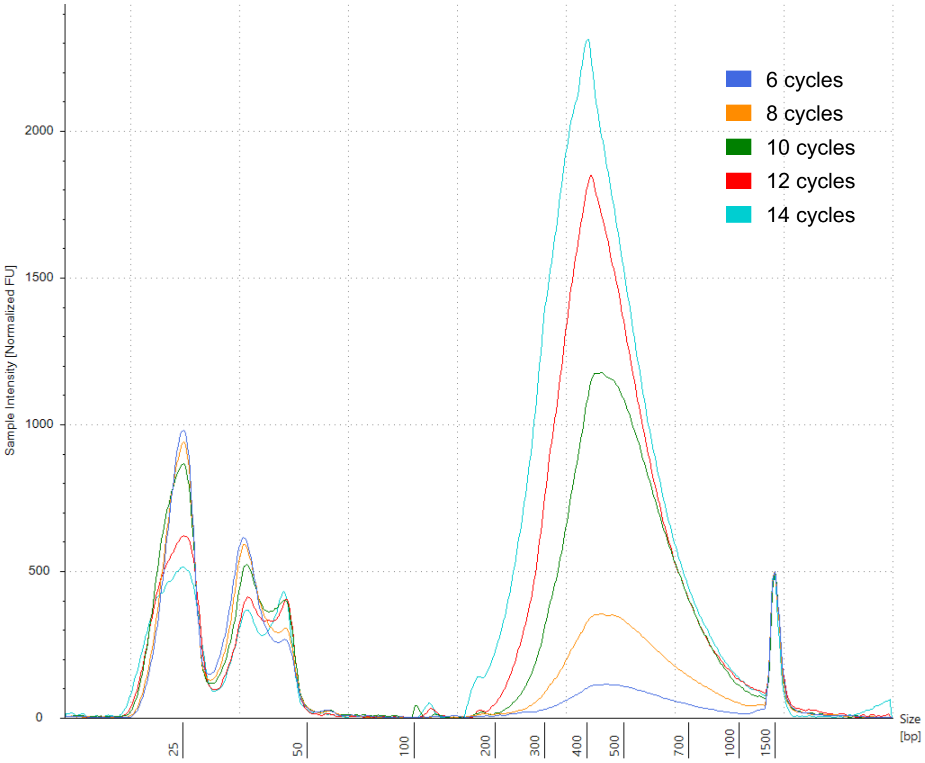

B

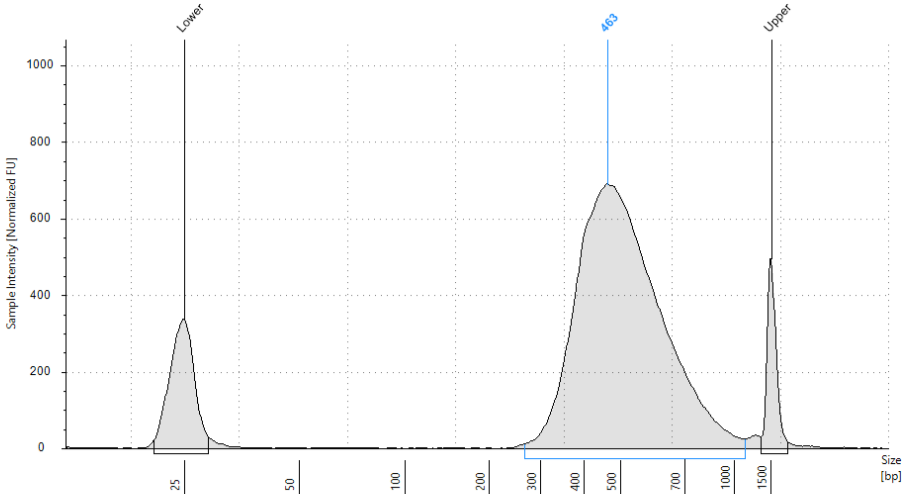

Fig. S19: Quality control of calibration RNA-sequencing library. (**A**) Overlay of electropherograms generated for libraries amplified with different PCR cycles. (**B**) Electropherogram of purified RNA-sequencing library. Data were generated with the High Sensitivity D1000 ScreenTape assay (Agilent Technologies, Santa Clara, CA, USA). The purified library exhibited a concentration of 2.97 ng/µl, based on Qubit 1X dsDNA High Sensitivity assay (Thermo Fisher Scientific, Waltham, MA, US

A

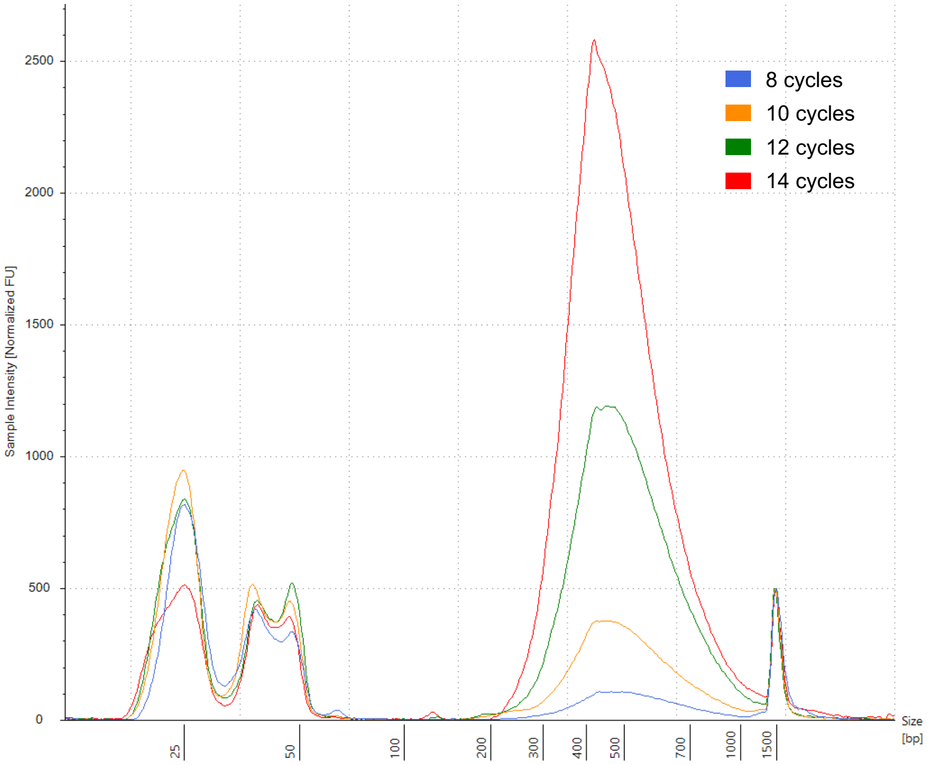

B

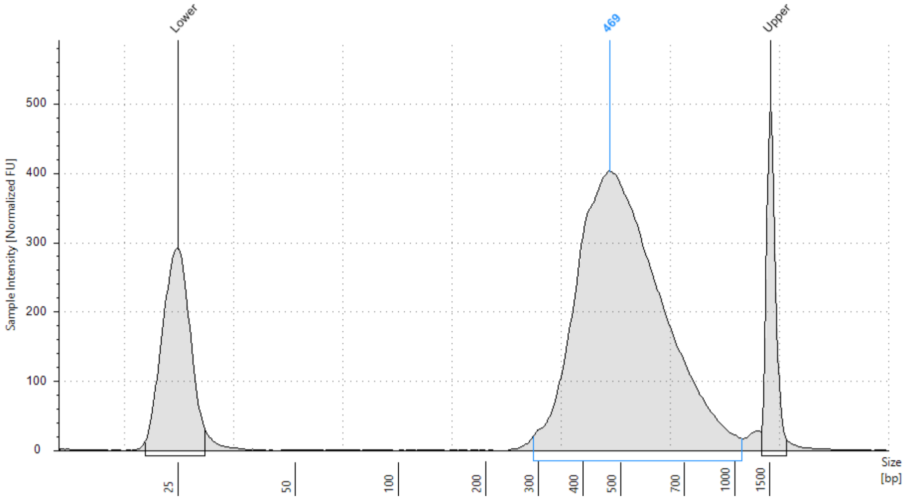

Fig. S20: Quality control of co-culture RNA-sequencing library. (**A**) Overlay of electropherograms generated for libraries amplified with different PCR cycles. (**B**) Electropherogram of purified co-culture RNA-sequencing library. Data were generated with the High Sensitivity D1000 ScreenTape assay (Agilent Technologies, Santa Clara, CA, USA). The purified library exhibited a concentration of 1.95 ng/µl, based on Qubit 1X dsDNA High Sensitivity assay (Thermo Fisher Scientific, Waltham, MA, USA).

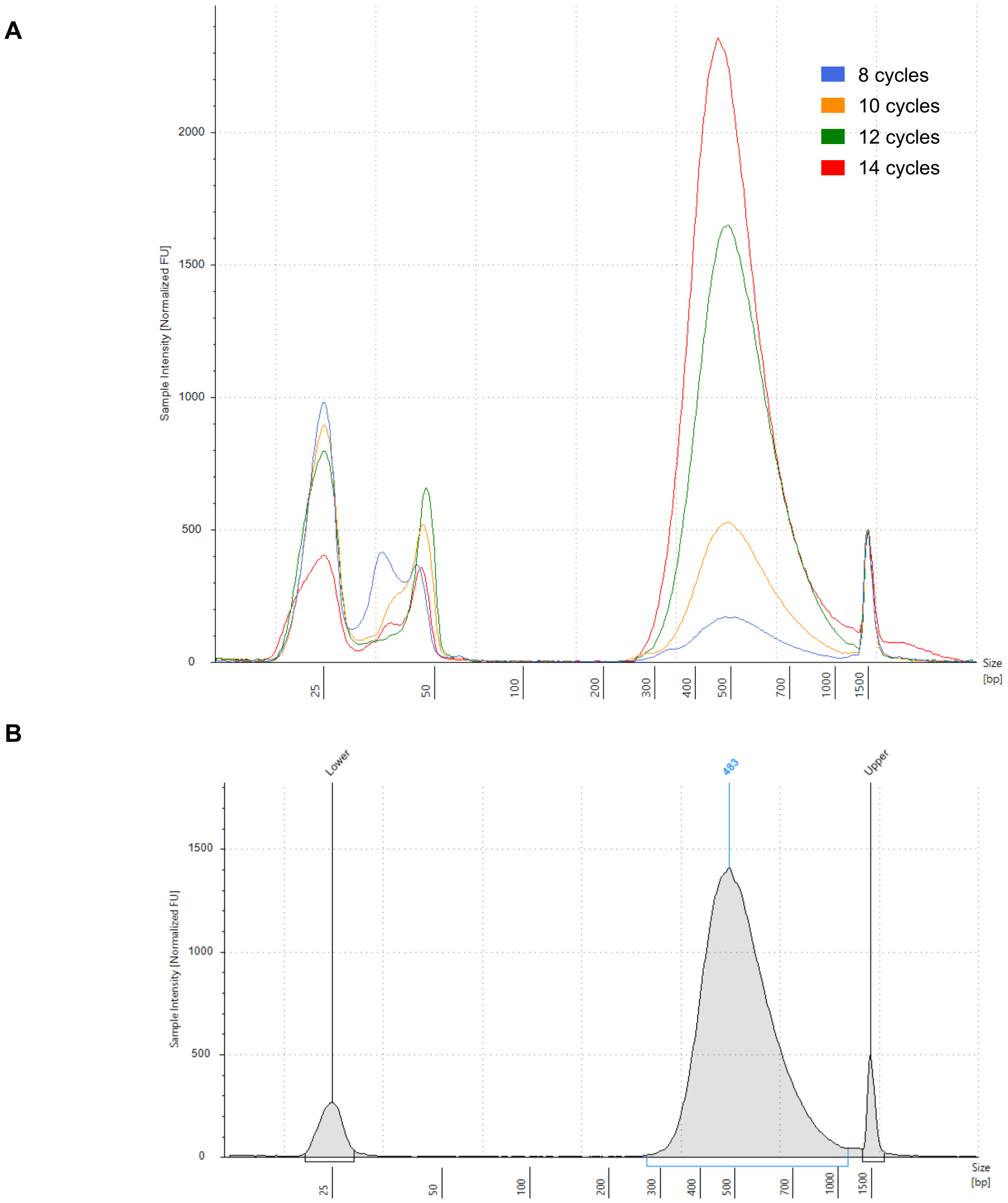

Fig. S21: Quality control of lag phase RNA-sequencing library. (**A**) Overlay of electropherograms generated for libraries amplified with different PCR cycles. (**B**) Electropherogram of purified lag phase RNA-sequencing library. Data were generated with the High Sensitivity D1000 ScreenTape assay (Agilent Technologies, Santa Clara, CA, USA). The purified library exhibited a concentration of 6.87 ng/µl, based on Qubit 1X dsDNA High Sensitivity assay (Thermo Fisher Scientific, Waltham, MA, USA).

Table S1: Sequencing yields of co-culture RNA-sequencing run.

	Cycles	Yield (Gbp)	% > Q30	Reads (M)	Reads passing filter (M)
Total	118	449.52	92.47	11,522	7,750
Read 1	64	244.139	91.60	5,761	3,875
Read 2	54	205.381	93.51	5,761	3,875

Table S2: Algal and bacterial read counts per sample of co-culture RNA-sequencing run.

Sample ID | replicate	Paired-end reads (after demultiplexing)	Paired-end reads (after cutadapt)	*Emiliania huxleyi* feature counts		*Phaeobacter inhibens* feature counts	
			total	non-rRNA	total	non-rRNA
**Samples with bacteria**						
co-culture day 04 | rep 1	394,493,942	393,728,523	359,778,099	356,869,622	392,785	389,312
co-culture day 04 | rep 2	82,196,551	82,043,741	75,153,364	74,496,050	386,857	384,464
co-culture day 04 | rep 3	54,291,481	54,204,536	49,610,317	49,154,523	378,032	375,186
co-culture day 06 | rep 1	345,539,973	344,369,218	312,349,003	310,170,228	211,954	206,050
co-culture day 06 | rep 2	356,221,349	355,409,747	322,917,441	321,222,154	207,351	204,656
co-culture day 06 | rep 3	441,433,612	440,437,425	402,400,748	398,384,445	227,898	221,668
co-culture day 09 | rep 1	470,080,858	468,929,331	424,468,336	420,392,969	503,047	500,235
co-culture day 09 | rep 2	330,459,894	329,572,907	297,519,953	295,253,525	426,485	423,914
co-culture day 09 | rep 3	566,156,857	564,972,691	512,310,003	508,633,530	430,678	427,880
co-culture day 11/12 | rep 1	46,392,253	45,102,945	22,016,187	20,011,612	14,968,563	14,592,173
co-culture day 11/12 | rep 2	39,800,421	38,213,653	19,992,702	18,072,191	10,833,700	10,613,582
co-culture day 11/12 | rep 3	43,723,899	41,714,542	22,274,938	20,246,580	11,197,490	10,953,892
glucose 1 | rep 1	4,023,097	4,022,839	15,974	15,963	3,785,341	3,531,816
glucose 1 | rep 2	4,161,707	4,161,252	12,306	12,227	3,972,265	3,794,756
glucose 1 | rep 3	4,355,903	4,355,619	4,968	4,926	4,168,592	3,946,424
glucose 2 | rep 1	4,464,358	4,463,708	1,899	1,794	4,090,721	3,753,760
glucose 2 | rep 2	4,571,322	4,569,155	7,835	7,719	4,242,487	3,723,849
glucose 2 | rep 3	4,471,401	4,470,558	40,114	39,608	4,127,911	3,408,995
**Samples without bacteria**						
axenic day 04 | rep 1	35,230,673	35,165,646	32,384,125	32,121,703	1,113	910
axenic day 04 | rep 2	33,432,598	33,352,029	30,413,097	30,071,739	383	211
axenic day 04 | rep 3	31,872,791	31,808,747	29,301,614	29,038,128	326	143
axenic day 06 | rep 1	39,435,756	39,334,511	36,033,965	35,753,053	189	154
axenic day 06 | rep 2	38,039,758	37,919,471	34,508,267	34,263,463	846	325
axenic day 06 | rep 3	40,960,442	40,882,202	37,357,061	37,018,630	358	292
axenic day 09 | rep 1	44,861,993	44,756,241	40,695,380	39,867,427	428	199
axenic day 09 | rep 2	45,383,172	45,280,741	41,185,604	40,801,350	656	247
axenic day 09 | rep 3	49,497,859	49,349,804	44,802,627	44,382,768	1,789	534
axenic day 11/12 | rep 1	41,514,091	41,407,679	37,368,996	37,188,706	8,579	8,139
axenic day 11/12 | rep 2	45,675,704	45,534,841	41,097,200	40,869,669	585	459
axenic day 11/12 | rep 3	40,972,617	40,868,606	36,908,030	36,679,879	831	551

* Bacterial reads in algal cultures without bacteria (axenic algal cultures) are due to algal genes with high similarity to bacterial genes. These reads account for roughly 1,000 reads in average per sample, which represent 0.003% of the total reads. Importantly, these reads should not be interpreted as a contamination since potential contaminations were closely monitored (see Materials and Methods).

Table S3: Methyl group metabolism genes.

Gene no.	RefSeq accession	Old accession	RefSeq product	GenBank product	Kegg product
1	PGA1_RS07200	PGA1_c14480	adenosylmethionine decarboxylase	S-adenosylmethionine decarboxylase SpeH	speD, AMD1; S-adenosylmethionine decarboxylase [EC:4.1.1.50]
2	PGA1_RS07195	PGA1_c14470	polyamine aminopropyltransferase	spermidine synthase SpeE	speE, SRM, SPE3; spermidine synthase [EC:2.5.1.16]
3	PGA1_RS07270	PGA1_c14630	trimethylamine methyltransferase family protein	trimethylamine methyltransferase MttB	mttB; trimethylamine---corrinoid protein Co-methyltransferase [EC:2.1.1.250]
4	PGA1_RS19035	PGA1_262p01860	acyl-CoA dehydrogenase	putative acyl-Co-A dehydrogenase	dmdC; 3-(methylsulfanyl)propanoyl-CoA dehydrogenase [EC:1.3.99.41]
5	PGA1_RS14040	PGA1_c28230	FAD-dependent oxidoreductase	dimethylglycine dehydrogenase DmgdH	DMGDH; dimethylglycine dehydrogenase [EC:1.5.8.4]
6	PGA1_RS18835	PGA1_262p01440	BCCT family transporter	BCCT transporter	opuD, betL; glycine betaine transporter
7	PGA1_RS19025	PGA1_262p01840	AMP-binding protein	ATP-dependent AMP binding enzyme	dmdB; 3-(methylthio)propionyl---CoA ligase [EC:6.2.1.44]
8	PGA1_RS19030	PGA1_262p01850	methylthioacryloyl-CoA hydratase	putative enoyl-CoA hydratase	dmdD; (methylthio)acryloyl-CoA hydratase [EC:4.2.1.155]
9	PGA1_RS18890	PGA1_262p01550	acyl-CoA dehydrogenase	acyl-CoA dehydrogenase	dmdC; 3-(methylsulfanyl)propanoyl-CoA dehydrogenase [EC:1.3.99.41]
10	PGA1_RS01910	PGA1_c03870	crotonyl-CoA carboxylase/reductase	crotonyl-CoA reductase	ccr; crotonyl-CoA carboxylase/reductase [EC:1.3.1.85]
11	PGA1_RS03240	PGA1_c06530	dihydroneopterin aldolase	dihydroneopterin aldolase-like protein	folB; 7,8-dihydroneopterin aldolase/epimerase/oxygenase [EC:4.1.2.25 5.1.99.8 1.13.11.81]
12	PGA1_RS18870	PGA1_262p01510	sarcosine oxidase subunit delta	sarcosine oxidase, delta subunit	mgdB; methylglutamate dehydrogenase subunit B [EC:1.5.99.5]
13	PGA1_RS09515	PGA1_c19170	sarcosine oxidase subunit beta family protein	sarcosine oxidase subunit beta	soxB; sarcosine oxidase, subunit beta [EC:1.5.3.24 1.5.3.1]
14	PGA1_RS18865	PGA1_262p01500	sarcosine oxidase subunit alpha family protein	sarcosine oxidase subunit alpha	mgdC; methylglutamate dehydrogenase subunit C [EC:1.5.99.5]
15	PGA1_RS11710	PGA1_c23540	O-succinylhomoserine sulfhydrylase	O-succinylhomoserine sulfhydrylase MetZ	metZ; O-succinylhomoserine sulfhydrylase [EC:2.5.1.-]
16	PGA1_RS10725	PGA1_c21660	choline dehydrogenase	choline dehydrogenase BetA	betA, CHDH; choline dehydrogenase [EC:1.1.99.1]
17	PGA1_RS18885	PGA1_262p01540	crotonase/enoyl-CoA hydratase family protein	putative enoyl-CoA hydratase / isomerase	dmdD; (methylthio)acryloyl-CoA hydratase [EC:4.2.1.155]
18	PGA1_RS09505	PGA1_c19150	sarcosine oxidase subunit alpha family protein	sarcosine oxidase subunit alpha	soxA; sarcosine oxidase, subunit alpha [EC:1.5.3.24 1.5.3.1]
19	PGA1_RS16700	PGA1_c33650	BCCT family transporter	putative transporter, BCCT family	
20	PGA1_RS18245	PGA1_262p00230	aldehyde dehydrogenase family protein	betaine aldehyde dehydrogenase BetB	ALDH; aldehyde dehydrogenase (NAD+) [EC:1.2.1.3]
21	PGA1_RS01875	PGA1_c03800	type III PLP-dependent enzyme	putative lysine/ornithine decarboxylase	E4.1.1.17, ODC1, speC, speF; ornithine decarboxylase [EC:4.1.1.17]
22	PGA1_RS00485	PGA1_c00990	2-amino-4-hydroxy-6-hydroxymethyldihydropteridine diphosphokinase	2-amino-4-hydroxy-6-hydroxymethyldihydropteridine pyrophosphokinase FolK	folK; 2-amino-4-hydroxy-6-hydroxymethyldihydropteridine diphosphokinase [EC:2.7.6.3]
23	PGA1_RS10730	PGA1_c21670	betaine-aldehyde dehydrogenase	betaine aldehyde dehydrogenase BetB	betB, gbsA; betaine-aldehyde dehydrogenase [EC:1.2.1.8]
24	PGA1_RS18875	PGA1_262p01520	sarcosine oxidase subunit beta family protein	sarcosine oxidase 3 subunit beta	mgdA; methylglutamate dehydrogenase subunit A [EC:1.5.99.5]
25	PGA1_RS19020	PGA1_262p01830	dimethylsulfoniopropionate demethylase	putative aminomethyltransferase	dmdA; dimethylsulfoniopropionate demethylase [EC:2.1.1.269]
26	PGA1_RS11745	PGA1_c23610	phosphoribosylamine--glycine ligase	phosphoribosylamine--glycine ligase PurD	purD; phosphoribosylamine---glycine ligase [EC:6.3.4.13]
27	PGA1_RS19415	PGA1_78p00230	oxidoreductase	putative NADH-ubiquinone oxidoreductase	ndhF; NAD(P)H-quinone oxidoreductase subunit 5 [EC:7.1.1.2]
28	PGA1_RS02485	PGA1_c05010	aromatic ring-hydroxylating dioxygenase subunit alpha	putative dioxygenase	stc2, hpbB; stachydrine N-demethylase [EC:1.14.13.247]
29	PGA1_RS02975	PGA1_c05990	formate--tetrahydrofolate ligase	formate--tetrahydrofolate ligase Fhs	fhs; formate--tetrahydrofolate ligase [EC:6.3.4.3]
30	PGA1_RS00080	PGA1_c00160	tRNA-Met	tRNA-Met	
31	PGA1_RS09500	PGA1_c19140	sarcosine oxidase subunit gamma	sarcosine oxidase-like protein	soxG; sarcosine oxidase, subunit gamma [EC:1.5.3.24 1.5.3.1]
32	PGA1_RS15740	PGA1_c31680	bifunctional folylpolyglutamate synthase/dihydrofolate synthase	putative bifunctional enzyme FolC	folC; dihydrofolate synthase / folylpolyglutamate synthase [EC:6.3.2.12 6.3.2.17]
33	PGA1_RS06610	PGA1_c13270	phosphoribosylglycinamide formyltransferase	phosphoribosylglycinamide formyltransferase PurN	purN; phosphoribosylglycinamide formyltransferase 1 [EC:2.1.2.2]
34	PGA1_RS16330	PGA1_c32880	RsmB/NOP family class I SAM-dependent RNA methyltransferase	putative ribosomal RNA small subunit methyltransferase B	rsmB, sun; 16S rRNA (cytosine967-C5)-methyltransferase [EC:2.1.1.176]
35	PGA1_RS12220	PGA1_c24550	aminodeoxychorismate synthase component I	putative para-aminobenzoate synthase component 1	pabB; para-aminobenzoate synthetase component I [EC:2.6.1.85]
36	PGA1_RS18235	PGA1_262p00210	GMC family oxidoreductase N-terminal domain-containing protein	alcohol dehydrogenase AlkJ	betA, CHDH; choline dehydrogenase [EC:1.1.99.1]
37	PGA1_RS09510	PGA1_c19160	sarcosine oxidase subunit delta	sarcosine oxidase 2 subunit delta	soxD; sarcosine oxidase, subunit delta [EC:1.5.3.24 1.5.3.1]
38	PGA1_RS19420	PGA1_78p00240	DUF2309 domain-containing protein	hypothetical protein	K09822; uncharacterized protein
39	PGA1_RS07565	PGA1_c15230	RsmB/NOP family class I SAM-dependent RNA methyltransferase	rRNA methyltransferase-like protein	rsmB, sun; 16S rRNA (cytosine967-C5)-methyltransferase [EC:2.1.1.176]
40	PGA1_RS16750	PGA1_c33750	glycine/betaine ABC transporter substrate-binding protein	putative glycine betaine transport system substrate binding protein	proX; glycine betaine/proline transport system substrate-binding protein
41	PGA1_RS17995	PGA1_c36240	23S rRNA (adenine(2503)-C(2))-methyltransferase RlmN	ribosomal RNA large subunit methyltransferase N	rlmN; 23S rRNA (adenine2503-C2)-methyltransferase [EC:2.1.1.192]
42	PGA1_RS02380	PGA1_c04800	tRNA (guanine(46)-N(7))-methyltransferase TrmB	tRNA (guanine-N(7)-)-methyltransferase TrmB	trmB, METTL1, TRM8; tRNA (guanine-N7-)-methyltransferase [EC:2.1.1.33]
43	PGA1_RS07175	PGA1_c14430	methylenetetrahydrofolate--tRNA-(uracil(54)-C(5))-methyltransferase (FADH(2)-oxidizing) TrmFO	methylenetetrahydrofolate-tRNA-(uracil-5-)-methyltransferase	trmFO, gid; methylenetetrahydrofolate--tRNA-(uracil-5-)-methyltransferase [EC:2.1.1.74]
44	PGA1_RS04145	PGA1_c08340	methionine--tRNA ligase	methionyl-tRNA synthetase MetG	MARS, metG; methionyl-tRNA synthetase [EC:6.1.1.10]
45	PGA1_RS02985	PGA1_c06010	bifunctional methylenetetrahydrofolate dehydrogenase/methenyltetrahydrofolate cyclohydrolase FolD	bifunctional protein FolD	folD; methylenetetrahydrofolate dehydrogenase (NADP+) / methenyltetrahydrofolate cyclohydrolase [EC:1.5.1.5 3.5.4.9]
46	PGA1_RS16755	PGA1_c33760	betaine/proline/choline family ABC transporter ATP-binding protein	putative glycine betaine transport ATP-binding protein	proV; glycine betaine/proline transport system ATP-binding protein [EC:7.6.2.9]
47	PGA1_RS13750	PGA1_c27650	formate dehydrogenase subunit alpha	putative formate dehydrogenase H	fdoG, fdhF, fdwA; formate dehydrogenase major subunit [EC:1.17.1.9]

Table S3 (continued)

Gene no.	RefSeq accession	Old accession	RefSeq product	GenBank product	Kegg product
48	PGA1_RS12125	PGA1_c24370	30S ribosomal protein S12 methylthiotransferase RimO	ribosomal protein S12 methylthiotransferase RimO	rimO; ribosomal protein S12 methylthiotransferase [EC:2.8.4.4]
49	PGA1_RS12780	PGA1_c25740	formate dehydrogenase subunit alpha	formate dehydrogenase H	fdoG, fdhF, fdwA; formate dehydrogenase major subunit [EC:1.17.1.9]
50	PGA1_RS13745	PGA1_c27640	formate dehydrogenase FDH3 subunit beta	formate dehydrogenase iron-sulfur subunit	fdoH, fdsB; formate dehydrogenase iron-sulfur subunit
51	PGA1_RS17285	PGA1_c34810	class I SAM-dependent RNA methyltransferase	putative RNA methyltransferase	rumA; 23S rRNA (uracil1939-C5)-methyltransferase [EC:2.1.1.190]
52	PGA1_RS10740	PGA1_c21690	choline transporter	transporter, BCCT family	opuD, betL; glycine betaine transporter
53	PGA1_RS16320	PGA1_c32860	bifunctional phosphoribosylaminoimidazolecarboxamide formyltransferase/IMP cyclohydrolase	bifunctional purine biosynthesis protein PurH	purH; phosphoribosylaminoimidazolecarboxamide formyltransferase / IMP cyclohydrolase [EC:2.1.2.3 3.5.4.10]
54	PGA1_RS10675	PGA1_c21540	acetyl/propionyl/methylcrotonyl-CoA carboxylase subunit alpha	propionyl-CoA carboxylase alpha chain	PCCA, pccA; propionyl-CoA carboxylase alpha chain [EC:6.4.1.3]
55	PGA1_RS06650	PGA1_c13350	cobalamin-binding protein	putative dimethylamine corrinoid protein	
56	PGA1_RS16860	PGA1_c33960	tRNA (N(6)-L-threonylcarbamoyladenosine(37)-C(2))-methylthiotransferase MtaB	MiaB-like tRNA modifying enzyme	mtaB; threonylcarbamoyladenosine tRNA methylthiotransferase MtaB [EC:2.8.4.5]
57	PGA1_RS10805	PGA1_c21820	5-formyltetrahydrofolate cyclo-ligase	5-formyltetrahydrofolate cycloligase-like protein	MTHFS; 5-formyltetrahydrofolate cyclo-ligase [EC:6.3.3.2]
58	PGA1_RS16760	PGA1_c33770	ABC transporter permease subunit	putative ABC transporter permease protein	proW; glycine betaine/proline transport system permease protein
59	PGA1_RS18110	PGA1_c36480	bifunctional demethylmenaquinone methyltransferase/2-methoxy-6-polyprenyl-1,4-benzoquinol methylase UbiE	ubiquinone/menaquinone biosynthesis methyltransferase UbiE	ubiE; demethylmenaquinone methyltransferase / 2-methoxy-6-polyprenyl-1,4-benzoquinol methylase [EC:2.1.1.163 2.1.1.201]
60	PGA1_RS00260	PGA1_c00520	tRNA (guanosine(37)-N1)-methyltransferase TrmD	tRNA (guanine-N(1)-)-methyltransferase TrmD	trmD; tRNA (guanine37-N1)-methyltransferase [EC:2.1.1.228]
61	PGA1_RS07960	PGA1_c16050	methylenetetrahydrofolate reductase	hypothetical protein	metF, MTHFR; methylenetetrahydrofolate reductase (NADH) [EC:1.5.1.54]
62	PGA1_RS01215	PGA1_c02510	S-(hydroxymethyl)glutathione dehydrogenase/class III alcohol dehydrogenase	S-(hydroxymethyl)glutathione dehydrogenase FrmA	frmA, ADH5, adhC; S-(hydroxymethyl)glutathione dehydrogenase / alcohol dehydrogenase [EC:1.1.1.284 1.1.1.1]
63	PGA1_RS07955	PGA1_c16040	methyltetrahydrofolate cobalamin methyltransferase	pterin binding domain-containing protein	metH, MTR; 5-methyltetrahydrofolate--homocysteine methyltransferase [EC:2.1.1.13]
64	PGA1_RS10490	PGA1_c21180	thymidylate synthase	thymidylate synthase ThyA	thyA, TYMS; thymidylate synthase [EC:2.1.1.45]
65	PGA1_RS13640	PGA1_c27430	cobalamin-independent methionine synthase II family protein	2-hydroxypropyl-CoM lyase-like protein	metE; 5-methyltetrahydropteroyltriglutamate--homocysteine methyltransferase [EC:2.1.1.14]
66	PGA1_RS01920	PGA1_c03890	autoinducer synthase	putative acyl-homoserine-lactone synthase RaiI	raiI; acyl homoserine lactone synthase [EC:2.3.1.184]
67	PGA1_RS05915	PGA1_c11870	serine hydroxymethyltransferase	serine hydroxymethyltransferase GlyA	glyA, SHMT; glycine hydroxymethyltransferase [EC:2.1.2.1]
68	PGA1_RS02760	PGA1_c05560	glycine cleavage system protein GcvH	glycine cleavage system protein GcvH	gcvH, GCSH; glycine cleavage system H protein
69	PGA1_RS15700	PGA1_c31600	S-formylglutathione hydrolase	S-formylglutathione hydrolase FghA	frmB, ESD, fghA; S-formylglutathione hydrolase [EC:3.1.2.12]
70	PGA1_RS16975	PGA1_c34190	class I SAM-dependent methyltransferase	methyltransferase-like protein	
71	PGA1_RS19450	PGA1_78p00300	glycine cleavage system protein GcvH	glycine cleavage system protein GcvH	gcvH, GCSH; glycine cleavage system H protein
72	PGA1_RS00460	PGA1_c00940	class I SAM-dependent methyltransferase	putative methyltransferase	
73	PGA1_RS19145	PGA1_262p02100	protein-glutamate O-methyltransferase	methyltransferase, cheR type	cheR; chemotaxis protein methyltransferase CheR [EC:2.1.1.80]
74	PGA1_RS04710	PGA1_c09490	16S rRNA (cytosine(1402)-N(4))-methyltransferase RsmH	S-adenosyl-L-methionine-dependent methyltransferase MraW	mraW, rsmH; 16S rRNA (cytosine1402-N4)-methyltransferase [EC:2.1.1.199]
75	PGA1_RS06910	PGA1_c13880	BCCT family transporter	putative transporter, BCCT family	TC.BCT; betaine/carnitine transporter, BCCT family
76	PGA1_RS17590	PGA1_c35420	adenosylhomocysteinase	adenosylhomocysteinase AhcY	E3.3.1.1, ahcY; adenosylhomocysteinase [EC:3.3.1.1]
77	PGA1_RS16660	PGA1_c33570	site-specific DNA-methyltransferase	modification methylase BabI	ccrM; modification methylase [EC:2.1.1.72]
78	PGA1_RS14200	PGA1_c28550	class I SAM-dependent methyltransferase	methyltransferase	evaC; methylation protein EvaC
79	PGA1_RS05830	PGA1_c11700	methylenetetrahydrofolate reductase [NAD(P)H]	5,10-methylenetetrahydrofolate reductase MetF	metF, MTHFR; methylenetetrahydrofolate reductase (NADH) [EC:1.5.1.54]
80	PGA1_RS19455	PGA1_78p00310	aminomethyl-transferring glycine dehydrogenase	glycine dehydrogenase	GLDC, gcvP; glycine dehydrogenase [EC:1.4.4.2]
81	PGA1_RS18555	PGA1_262p00860	tRNA (N6-threonylcarbamoyladenosine(37)-N6)-methyltransferase TrmO	hypothetical protein	TRMO, trmO; tRNA (adenine37-N6)-methyltransferase [EC:2.1.1.-]
82	PGA1_RS06660	PGA1_c13370	betaine--homocysteine S-methyltransferase	putative homocysteine S-methyltransferase	metH, MTR; 5-methyltetrahydrofolate--homocysteine methyltransferase [EC:2.1.1.13]
83	PGA1_RS02370	PGA1_c04780	methionine adenosyltransferase	S-adenosylmethionine synthetase MetK	metK, MAT; S-adenosylmethionine synthetase [EC:2.5.1.6]

Table S4. Comparison of lag phase shortening responses in *P. inhibens* bacteria induced by various compounds. Differences in lag phase shortening upon treatment with 2 µM of various compounds were reported in Figs. 3A and 3B, and the statistical significance of the difference between responses was calculated. The comparison of lag phase shortening induced by various methylated compounds within the same group ("Methylated compound groups" in the table) was assessed using Tukey's multiple comparisons test. In cases where there were only two compounds in a group, an unpaired two-tailed t-test was conducted. *p*-value thresholds: **** ≤ 0.0001, *** ≤ 0.001, ** ≤ 0.01, * ≤ 0.05, ns [not significant] > 0.05.

Methylated compound groups	Compounds comparison	Significance	*p*-value
DMSP (and analogues)	Acetate vs. Propionate	ns	0.5294
	Acetate vs. MPA	ns	0.9998
	Acetate vs. 3-MMPA	**	0.0019
	Acetate vs. DMSP	****	<0.0001
	Propionate vs. MPA	ns	0.4357
	Propionate vs. 3-MMPA	***	0.0001
	Propionate vs. DMSP	****	<0.0001
	MPA vs. 3-MMPA	**	0.0027
	MPA vs. DMSP	****	<0.0001
	3-MMPA vs. DMSP	**	0.0033
N-methylated quaternary ammonium compounds (and analogues)	Betaine vs. Choline	ns	0.2249
	Betaine vs. DMG	ns	0.6042
	Betaine vs. Sarcosine	***	0.0003
	Betaine vs. Alanine	****	<0.0001
	Betaine vs. Carnitine	ns	0.9925
	Choline vs. DMG	*	0.0101
	Choline vs. Sarcosine	****	<0.0001
	Choline vs. Alanine	****	<0.0001
	Choline vs. Carnitine	ns	0.4946
	DMG vs. Sarcosine	**	0.0079
	DMG vs. Alanine	***	0.0002
	DMG vs. Carnitine	ns	0.2993
	Sarcosine vs. Alanine	ns	0.5489
	Sarcosine vs. Carnitine	****	<0.0001
	Alanine vs. Carnitine	****	<0.0001
Stachydrine (and analogues)	Proline vs. Stachydrine	****	<0.0001
N-methylated aromatic compounds (and analogues)	Trigonelline vs. Homarine	ns	0.7399
	Trigonelline vs. Nicotinic acid	*	0.0316
	Homarine vs. Nicotinic acid	*	0.01
S-methylated sulfonium compounds (and analogues)	Gonyol vs. DMSA	*	0.0299
	Gonyol vs. Cysteine	***	0.0001
	DMSA vs. Cysteine	**	0.0052
One-carbon compounds	Methanol vs. Methanthiol	ns	0.7912
C1 group donating amino acids	Glycine vs. Serine	ns	0.6225
	Glycine vs. Methionine	****	<0.0001
	Serine vs. Methionine	****	<0.0001

Table S5: Estimated methyl group requirements per *P. inhibens* bacterial cell. Values based on *E. coli* (Neidhardt *et al.,*[5]). Calculations indicate that a single *P. inhibens* cell requires 190.1 amol of C1 groups to synthesize the building blocks needed for one cell duplication. If a freshly initiated *P. inhibens* culture, at a density of 25,000 CFU/mL, assimilates all available DMSP methyl groups during the lag phase, then the bacterial population requires 2.4 nM of DMSP to duplicate. Indeed, similar DMSP concentrations—2 nM—induced significant lag phase shortening (Fig. 2C).

Methyl groups can also undergo dissimilation to generate ATP, producing one ATP per methyl group (Fig. 1C; gene 29). According to the provided growth-associated maintenance cost (GAM) in the metabolic model of *P. inhibens*[6], a single *P. inhibens* cell requires 23,800 amol ATP to conduct one cell duplication during exponential growth with glucose. If a freshly initiated *P. inhibens* culture (25,000 CFU/mL) were to rely entirely on the dissimilation of DMSP methyl groups during the lag phase to meet its ATP requirements, the bacterial population would need 298 nM of DMSP to fulfill 100% of the ATP costs for one duplication. In our experiments, 2 nM DMSP already induced significant lag phase shortening (Fig. 2C). This concentration can cover only 0.7% of the bacterial ATP needs.

Importantly, the presented values in this calculation are estimations and may likely underestimate bacterial requirements. However, it is noteworthy that the difference in assimilatory versus dissimilatory needs, which can be both fulfilled by methyl groups, spans two orders of magnitude.

**Building blocks that are C1 sinks**	**ATP (RNA)**	**GTP (RNA)**	**dATP (DNA)**	**dGTP (DNA)**	**dTTP (DNA)**	**Histidine[1] (proteins)**	**Methionine (proteins)**
Class	Purine	Purine	Purine	Purine	Thymine	Amino acid	Amino acid
**Experimentally determined amount of building block in *E. coli* B/r[3]**							
[µmol building block/ g dried cells]	165.0	203.0	24.7	25.4	24.7	90.0	146.0
[amol building block/ cell]	46.2	56.8	6.9	7.1	6.9	25.2	40.9
**Incorporation of C1 groups during building block synthesis in *P. inhibens***							
Amount of C1 groups incorporated per building block	2	2	2	2	1	1	1
Key enzyme(s)	1: Phosphoribosyl-glycinamide formyltransferase 2: Phosphoribosyl-aminoimidazolecarboxamide formyltransferase	1: Phosphoribosyl-glycinamide formyltransferase 2: Phosphoribosyl-aminoimidazolecarboxamide formyltransferase	1: Phosphoribosyl-glycinamide formyltransferase 2: Phosphoribosyl-aminoimidazolecarboxamide formyltransferase	1: Phosphoribosyl-glycinamide formyltransferase 2: Phosphoribosyl-aminoimidazolecarboxamide formyltransferase	Thymidylate synthase	Phosphoribosyl-aminoimidazolecarboxamide formyltransferase	Methionine synthase
C1 group donors	1: CHO-THF 2: CHO-THF	1: CHO-THF 2: CHO-THF	1: CHO-THF 2: CHO-THF	1: CHO-THF 2: CHO-THF	CH_2-THF	CHO-THF	CH_3-cobalamin, DMSP, betaine[2]
Accession (gene name, RefSeq, GennBank, protein)	1: *purN*, PGA1_RS06610, PGA1_c13270, WP_014874448.1 2: *purH*, PGA1_RS16320, PGA1_c32860, WP_014881359.1	1: *purN*, PGA1_RS06610, PGA1_c13270, WP_014874448.1 2: *purH*, PGA1_RS16320, PGA1_c32860, WP_014881359.1	1: *purN*, PGA1_RS06610, PGA1_c13270, WP_014874448.1 2: *purH*, PGA1_RS16320, PGA1_c32860, WP_014881359.1	1: *purN*, PGA1_RS06610, PGA1_c13270, WP_014874448.1 2: *purH*, PGA1_RS16320, PGA1_c32860, WP_014881359.1	*thyA*, PGA1_RS10490, PGA1_c21180, WP_014880433.1	*purH*, PGA1_RS16320, PGA1_c32860, WP_014881359.1	*bmt*, PGA1_RS06660, PGA1_c13370, WP_014879827.1
Genes	1: 33 2: 53	1: 33 2: 53	1: 33 2: 53	1: 33 2: 53	64	53	82
Amount of C1 groups produced per building block[3]	1	1	1	1	0	0	0
Net C1 group requirements per cell [amol C1 group/cell]	46.2	56.8	6.9	7.1	6.9	25.2	40.9

[1] Consumption of C1 groups during histidine synthesis occurs indirectly. Histidine is synthesized by condensing the adenine backbone of ATP with the ribose sugar 5-phospho-α-D-ribose 1-diphosphate (PRPP). The side product of histidine synthesis is 5-amino-1-(5-phospho-D-ribosyl)imidazole-4-carboxamide (AICAR). AICAR is channeled into the lower branch of the purine synthesis pathway to regenerate the initially consumed ATP. Thus, ATP regeneration consumes one formyl group per synthesized histidine.
histidine.

[2] Methionine synthesis in *P. inhibens* was reported to depend on the methionine synthase enzyme Bmt and a cobalamin binding protein. It was thus concluded that CH_3-cobalamine is the methyl group donor for Bmt[7]. However, the utilization of other methyl group donors by Bmt cannot be excluded[8].

[3] Purine synthesis consumes two C1 groups and releases one C1 group per produced building block[5]. The release of one C1 group arises from the incorporation of glycine during purine synthesis (Fig. 1C; gene 26: phosphoribosylamine—glycine ligase). Glycine is produced by converting glucose (via 3-phosphoglycerate) to serine and then to glycine, which releases one C1 group (Fig. 1C; gene 67: serine hydroxymethyltransferase).

Table S6: Sequencing yields of lag phase RNA-sequencing run.

	Cycles	Yield (Gbp)	% > Q30	Reads (M)	Reads passing filter (M)
Total	168	20.77	90.78	338.1	249.3
Read 1	89	11.01	90.11	169.1	124.7
Read 2	79	9.76	91.55	169.1	124.7

Table S7: Bacterial read counts per sample of lag phase RNA-sequencing run.

Sample ID | replicate	Paired-end reads (after demultiplexing)	Paired-end reads (after cutadapt)	*Phaeobacter inhibens* feature counts	
			total	non-rRNA
Control (15 min lag) | rep 1	5,173,156	5,172,928	4,912,488	4,786,267
Control (15 min lag) | rep 2	6,256,704	6,256,419	5,938,559	5,783,725
Control (15 min lag) | rep 3	5,656,899	5,656,722	5,360,067	5,214,107
DMSP (15 min lag) | rep 1	7,299,641	7,299,366	6,798,582	6,591,949
DMSP (15 min lag) | rep 2	4,585,425	4,585,264	4,232,884	4,090,929
DMSP (15 min lag) | rep 3	7,399,140	7,398,864	6,915,209	6,708,565
Control (40 min lag) | rep 1	5,816,609	5,816,342	5,537,252	5,402,090
Control (40 min lag) | rep 2	6,157,332	6,157,141	5,857,923	5,714,672
Control (40 min lag) | rep 3	5,616,052	5,615,798	5,376,958	5,257,722
DMSP (40 min lag) | rep 1	5,862,598	5,862,395	5,554,342	5,414,854
DMSP (40 min lag) | rep 2	6,068,667	6,068,472	5,711,286	5,562,658
DMSP (40 min lag) | rep 3	5,405,311	5,405,127	5,067,317	4,933,000

Table S8: Bacterial genomes used for analysis of methyl group-related metabolism in algal-associated and plant-associated bacteria (see fig. S18).

**Bacteria**	**NCBI RefSeq / repository**
Labrenzia sp. THAF82	GCF_009363435.1
Vibrio harveyi SB1	GCF_030060435.1
Marinomonas algarum E8	GCF_020532605.1
Pseudoalteromonas phenolica KCTC 12086	GCF_001444405.1
Bacillus subtilis 168	GCF_000009045.1
Pseudomonas koreensis LMG 21318	GCF_900101415.1
Pseudomonas aeruginosa PAO1	GCF_000006765.1
Ewingella americana CCUG 14506T	GCF_008693045.1
Lelliottia amnigena FDAARGOS_1445	GCF_019355955.1
Klebsiella aerogenes Ka37751 (previously *Enterobacter aerogenes*)	GCF_007632255.1
Sulfitobacter pontiacus iR4	https://doi.org/10.5281/zenodo.7520022

Table S9: Key Resources Table

REAGENT or RESOURCE	SOURCE	IDENTIFIER
Bacterial and algal strains		
Emiliania huxleyi CCMP3266	Bigelow National Center for Marine Algae and Microbiota (NCMA)	N/A
Phaeobacter inhibens DSM 17395; wild type	German Collection of Microorganism and Cell Cultures (DSMZ)	N/A
Phaeobacter inhibens DSM 17395; *dmdA::aacC1* (Gm^r) KO mutant	This paper	ES146
Vibrio sp. DT22	Despotović *et al*.[9]	N/A
Labrenzia sp. DT40	Despotović *et al*.[9]	N/A
Phaeobacter sp. DT104	Despotović *et al*.[9]	N/A
Pseudoalteromonas sp. DT35	Despotović *et al*.[9]	N/A
Labrenzia sp. DT112	Despotović *et al*.[9]	N/A
Vibrio sp. DT18	Despotović *et al*.[9]	N/A
Marinomonas sp. DT74	Despotović *et al*.[9]	N/A
Sulfitobacter pontiacus iR4	Beiralas *et al*.[10]	N/A
Bacillus subtilis 168	Bacteriology Unit of The Weizmann Institute of Science, Israel	N/A
Pseudomonas koreensis PK	Friedman lab strain collection[11]	N/A
Ewingella americana EA	Friedman lab strain collection[11]	N/A
Pseudomonas aeruginosa PAO1	Friedman lab strain collection	N/A
Lelliottia amnigena LA	Friedman lab strain collection[11]	N/A
Enterobacter aerogenes Ea	Friedman lab strain collection	N/A
Chemicals, peptides, and recombinant proteins		
Yeast extract	Sigma-Aldrich, Merck	Cat#Y1625
Tryptone	Sigma-Aldrich, Merck	Cat#T7293
Sea salts	Sigma-Aldrich, Merck	Cat#S9883
Bacto™ Agar	BD	Cat#214010
L1 Medium Kit - Si (includes f/2 vitamins, L1 trace elements and L1 nutrients solutions)	Bigelow National Center for Marine Algae and Microbiota (NCMA)	Cat#MKL11000L-Si
D-(+)-Glucose	Sigma-Aldrich, Merck	Cat#G7021
Dimethylpropiothetin HCl, ≥ 96.0%, (dimethylsulfoniopropionate, DMSP)	Sigma-Aldrich, Merck	Cat#80828; CAS: 4337-33-1
3-(Methylthio)propionic acid, 98%, (3-*S*-methylmercaptopropionate, 3-MMPA)	Alfa Aesar	Cat#L12861; CAS: 646-01-5
3-Mercaptopropionic acid, 99%, (MPA)	Alfa Aesar	Cat#A13261; CAS: 107-96-0
Sodium propionate, ≥99%	Sigma-Aldrich, Merck	Cat#P1880; CAS: 137-40-6

Sodium acetate, ≥99%	Sigma-Aldrich, Merck	Cat#S5636;<br>CAS: 127-09-3
Choline chloride, ≥98%	Sigma-Aldrich, Merck	Cat#C7527;<br>CAS: 67-48-1
L-Carnitine, ≥98%	Sigma-Aldrich, Merck	Cat#C0158;<br>CAS: 541-15-1
Betaine, ≥99%	Sigma-Aldrich, Merck	Cat#61962;<br>CAS: 107-43-7
N,*N*-Dimethylglycine HCl, 99%	Acros Organics	Cat#204660050;<br>CAS: 2491-06-7
Sarcosine, 98%	Alfa Aesar	Cat#A14594;<br>CAS: 107-97-1
L-Alanine, ≥98%	Sigma-Aldrich, Merck	Cat#A7627;<br>CAS: 56-41-7
Stachydrine HCl, 98%, (proline betaine)	Angene	Cat#AG00ILKM;<br>CAS: 4136-37-2
L-Proline, 99%	Alfa Aesar	Cat#A10199;<br>CAS: 147-85-3
Homarine, ≥95%	Santa Cruz Biotechnology	Cat#sc-470671;<br>CAS: 445-30-7
Trigonelline, 98%	Angene	Cat#AG003V2L;<br>CAS: 6138-41-6
Nicotinic acid, ≥98%	Sigma-Aldrich, Merck	Cat#N4126;<br>CAS: 59-67-6
Gonyol	Kindly provided by Kathleen Thume and Georg Pohnert; synthesized according to Gebser and Pohnert[12]	N/A
Sulfobetaine HCl, (DMSA, dimethylsulfonioacetate, dimethylthetin)	Toronto Research Chemicals	Cat#S699400;<br>CAS: 4727-41-7
L-Cysteine, 98+%	Acros Organics	Cat#173600250;<br>CAS: 52-90-4
Sodium methanthiolate, ≥90% (MeSH)	Sigma-Aldrich, Merck	Cat#71742;<br>CAS: 5188-07-8
Methanol, ≥99.9%	Sigma-Aldrich, Merck	Cat#1060072500;<br>CAS: 67-56-1
Glycine, 99%	Acros Organics	Cat#385900500;<br>CAS: 56-40-6
L-Serine, 99%	Acros Organics	Cat#132660250;<br>CAS: 56-45-1
L-Methionine, 98+%	Acros Organics	Cat#166160250;<br>CAS: 63-68-3
DL-Methionine methylsulfonium Cl, 99%, (*S*-methylmethionine; SMM)	mcule	Cat#MCULE-1226059178;<br>CAS: 3493-12-7
L-Homocysteine, 95%	Angene	Cat#AG0037FN<br>CAS: 6027-13-0
Cell Free Amino Acid Mixture - ^{13}C,^{15}N	Sigma-Aldrich, Merck	Cat#767964
β-Mercaptoethanol, 99%	Sigma-Aldrich, Merck	Cat#M3148<br>CAS: 60-24-2
n-Hexadecane, 99%	Alfa Aeser	Cat#A10322
Critical commercial assays, kits and consumables		

Buffer RLT	QIAGEN	Cat#79216
Low Binding Silica Grinding Beads (100 µm)	Spex® SamplePrep	Cat#2168
ISOLATE II RNA Mini Kit	Bioline, Meridian Bioscience	Cat#BIO-52072
TURBO™ DNase (2 U/µL)	Invitrogen, Thermo Fisher Scientific	Cat#AM2238
RNAClean XP magnetic beads	Beckman Coulter	Cat#A63987
Qubit™ RNA High Sensitivity (HS) Assay Kit	Invitrogen, Thermo Fisher Scientific	Cat#Q32852
Qubit™ 1X dsDNA High Sensitivity (HS) Assay Kit	Invitrogen, Thermo Fisher Scientific	Cat#Q33230
RNA ScreenTape Analysis	Agilent Technologies	Cat#5067-5576
High Sensitivity D1000 ScreenTape	Agilent Technologies	Cat#5067-5584
riboPOOL depletion kit with Pan-Bacteria probes	siTOOLs Biotech	Cat#dp-K024-000026
riboPOOL depletion kit with *E. huxleyi* probes	siTOOLs Biotech	Cat#dp-K024-000033
FastAP thermosensitive alkaline phosphatase, 1 U/µl	Thermo Fisher Scientific	Cat#EF0651
RNA Clean & Concentrator-5 columns	Zymo Research	Cat#R1015
SuperScript™ IV First-Strand Synthesis System	Invitrogen, Thermo Fisher Scientific	Cat#18091200
T4 RNA ligase 1 (ssRNA Ligase), 30,000 U/ml	New England Biolabs	Cat#M0437M
T4 polynucleotide kinase (PNK), 10 U/µl	New England Biolabs	Cat#M0201S
RNase inhibitor, murine, 40 U/µl	New England Biolabs	Cat#M0314S
SUPERase-IN, 20 U/µl	Invitrogen, Thermo Fisher Scientific	Cat#AM2694
AccuPrime™ *Taq* DNA Polymerase, high fidelity	Invitrogen, Thermo Fisher Scientific	Cat#12346086
NovaSeq 6000 S1 Reagent Kit (100 cycles)	Illumina	Cat#20012865
NextSeq 500/550 Mid Output Kit v2.5 (150 Cycles)	Illumina	Cat#20024904
NucleoSpin Gel and PCR Clean-up Kit	MACHEREY-NAGEL	Cat#740609
Phusion High Fidelity DNA polymerase	Thermo Fisher Scientific	Cat#F530S
QIAprep Spin Miniprep Kit	QIAGEN	Cat#271040
Millex®-GV Filter Unit (Sterile), pore size 0.22 µm	Millipore, Merck	Cat#SLGV033RS
Deposited data		
Co-culture RNA-sequencing reads (dual RNA-seq); demultiplexed, trimmed, QC filtered	This paper	BioProject: PRJNA976961
Lag phase RNA-sequencing reads; demultiplexed, trimmed, QC filtered	This paper	BioProject: PRJNA977030
Emiliania huxleyi CCMP3266 sGenome assembly	Sperfeld *et al.*[13]	https://zenodo.org/record/5702921
Emiliania huxleyi CCMP3266 sGenome annotation version 2	This paper	data S3
Phaeobacter inhibens DSM 17395 genome assembly	Thole *et al.*[14]	assembly accession: GCA_000154765.2

Phaeobacter inhibens DSM 17395 genome annotations (RefSeq)	O'Leary *et al.*[15]	https://ftp.ncbi.nlm.nih.gov/genomes/refseq/bacteria/Phaeobacter_inhibens/latest_assembly_versions/GCF_000154765.2_ASM15476v2/
Phaeobacter inhibens DSM 17395 genome annotations (GenBank)	Thole *et al.*[14]	https://ftp.ncbi.nlm.nih.gov/genomes/genbank/bacteria/Phaeobacter_inhibens/latest_assembly_versions/GCA_000154765.2_ASM15476v2/
Phaeobacter inhibens DSM 17395 KEGG metabolic pathways annotation map	Kanehisa and Goto[16]	https://www.genome.jp/pathway/pga01100 (pga01100)
Phaeobacter inhibens DSM 17395 BioCyc annotations (v27.0)	Karp *et al.*[17]	https://biocyc.org/organism-summary?object=GCF_000154765
Oligonucleotides		
1. *dmdA* KO upstream forward primer with homology to pCR™II-TOPO™: GTAACGGCCGCCAGTGTGCTGGAATTCGCCCTTGCCGCCGCATGGCAAACACCG	This paper	N/A
2. *dmdA* KO upstream reverse primer with homology to gentamycin cassette: GCATTACAGTTTACGAACCGAACAGGCTTATGTCAATCAGGTGCTCATGGTTTTGGCGGC	This paper	N/A
3. *dmdA* KO downstream forward primer with homology to gentamycin cassette: GGTGGGCTGCCCTTCCTGGTTGGCTTGGTTTCCGATGCAGTTTGCGTGGTGCG	This paper	N/A
4. *dmdA* KO downstream reverse primer with homology to pCR™II-TOPO™: CCGCCAGTGTGATGGATATCTGCAGAATTCGCCCTTCGCGCGTCGGCGATCTCTG	This paper	N/A
5. *dmdA* KO verification forward primer: GGACGCCATCGCGCTGAAC	This paper	N/A
6. *dmdA* KO verification reverse primer: GGCTGGCTTCTCATGATCTCGC	This paper	N/A
7. Gentamycin cassette forward primer: GTTGACATAAGCCTGTTCG	This paper	N/A
8. Gentamycin cassette reverse primer: GTTAGGTGGCGGTACTTGG	This paper	N/A
9. Gentamycin cassette verification forward primer: GTGCAAGCAGATTACGGTGACG	This paper	N/A
10. Gentamycin cassette verification reverse primer: GAGCCTACATGTGCGAATGATGC	This paper	N/A
Recombinant DNA		
pDN12: *dmdA* KO plasmid (Upstream *dmdA*-Gm-Downstream *dmdA*-TOPOII)	This paper	N/A
pBBR1MCS-5	Kovach *et al.*[18]	N/A

pCR™II-TOPO™	Invitrogen	Cat#K466040
Software and algorithms		
fastq-multx; v1.3.1	Aronesty[19]	https://github.com/brwnj/fastq-multx
Cutadapt; v1.18	Martin[20]	https://anaconda.org/bioconda/cutadapt
STAR; v2.7.5c	Dobin *et al.*[21]	https://github.com/alexdobin/STAR
featureCounts; v2.0.0	Liao *et al.*[22]	https://subread.sourceforge.net/
DeSeq2; v1.36.0	Love *et al.*[23]	https://bioconductor.org/packages/release/bioc/html/DESeq2.html
R; v4.2.0	R Core Team[24]	https://www.R-project.org/
ggplot2; v3.4.1	Wickham[25]	https://ggplot2.tidyverse.org
Growthcurver; v0.3.1	Sprouffske and Wagner[26]	https://github.com/sprouffske/growthcurver
multcomp; v1.4-23	Hothorn *et al.*[27]	https://cran.r-project.org/web/packages/multcomp/index.html
Compound Discoverer™ Software; v3.3	Thermo Fisher Scientific	Cat#OPTON-31061
OrthoFinder	Emms and Kelly [1]	https://github.com/davidemms/OrthoFinder
FastME 2.0	Lefort *et al.*[3]	http://www.atgc-montpellier.fr/fastme/
MCL	Van Dongen *et al.* [2]	https://github.com/micans/mcl
Diamond	Buchfink *et al.*[4]	https://github.com/bbuchfink/diamond
apglm method	Zhu *et al.*[28]	https://github.com/mikelove/apeglmPaper
Databases and collections		
BioCyc Pathway/Genome Database Collection	Karp *et al.*[17]	N/A
NCBI Reference Sequence Database (RefSeq)	O'Leary *et al.*[15]	N/A
Kyoto Encyclopedia of Genes and Genomes (KEGG)	Kanehisa and Goto[16]	N/A
GenBank submitter annotations	Thole *et al.*[14]	N/A
UniProt Archive (UniParc)	The UniProt Consortium[29]	N/A
UniProt database	Paysan-Lafosse *et al.*[30]	N/A
Basic Local Aligment Search Tool (BLAST)	Altschul *et al.*[31]	N/A

Data S1: *P. inhibens* feature table (genes) with results from co-cultivation RNA-sequencing run. The dataset includes bacterial gene accession numbers, functional annotations, transcript abundances (TPM normalized read counts) and results of DESeq2 differential gene expression analysis. (Fig. 1, figs. S1A, S3-S4, tables S1-S2).
https://doi.org/10.5281/zenodo.10980548

Data S2: *P. inhibens* feature table (genes) with results from lag phase RNA-sequencing run. The dataset includes bacterial gene accession numbers, functional annotations, transcript abundances (TPM normalized read counts) and results of DESeq2 differential gene expression analysis. (Fig. 4A, figs. S10-S13, tables S6-S7).
https://doi.org/10.5281/zenodo.10980548

Data S3: *Emiliania huxleyi* CCMP3266 sGenome gene annotation file version 2 (GFF3 format).
https://doi.org/10.5281/zenodo.10980548

Data S4: Feature quantification obtained using Compound Discoverer. The table presents the output analysis using Compound Discoverer (v3.3) with a putative identification of metabolites. Each identified metabolite (each row) contains a sub-table under the + tab (on the left side) that specifies the feature quantification in each analyzed sample. Sample ID appears in the “Study File ID” column in each sub-table. Values in the columns “Exchange Rate [%]: 0” and “Exchange Rate [%]: 1” represent relative abundances of the molecules in their unlabeled form and with single ^{13}C-label (M+1 isotopologue), respectively. Compounds marked with “1” in the “Tags” column were further validated using standards. The average M+1 isotope abundance [%] for *S*-Adenosylmethionine (SAM) and 5'-*S*-Methyl-5'-thioadenosine (MTA)—as reported in Fig. 4D—were calculated by averaging the values in the “Exchange Rate [%]: 1” column from samples supplemented with ^{13}C-labeled and unlabeled DMSP, respectively. Detailed information regarding the isotope abundances of SAM and MTA can be found in the tab “SAM and MTA isotope abundance” in the table. In the tab “Features Positive Mode” samples F10, F12, F14, and F16 were supplemented with ^{13}C-labeled DMSP and samples F2, F4, F6, F8 were supplemented with unlabeled DMSP. In the tab “Features Negative Mode” samples F2, F3, F4, and F5 were supplemented with ^{13}C-labeled DMSP and samples F10, F11, F12, F13 were supplemented with unlabeled DMSP.
https://doi.org/10.5281/zenodo.10980548

References for Supplementary Materials

1. Emms, D.M., and Kelly, S. (2019). OrthoFinder: phylogenetic orthology inference for comparative genomics. Genome Biology *20*, 238. 10.1186/s13059-019-1832-y.
2. Van Dongen, S. (2008). Graph Clustering Via a Discrete Uncoupling Process. SIAM J. Matrix Anal. Appl. *30*, 121–141. 10.1137/040608635.
3. Lefort, V., Desper, R., and Gascuel, O. (2015). FastME 2.0: A Comprehensive, Accurate, and Fast Distance-Based Phylogeny Inference Program. Molecular Biology and Evolution *32*, 2798–2800. 10.1093/molbev/msv150.
4. Buchfink, B., Reuter, K., and Drost, H.-G. (2021). Sensitive protein alignments at tree-of-life scale using DIAMOND. Nat Methods *18*, 366–368. 10.1038/s41592-021-01101-x.
5. Neidhardt, F.C., Ingraham, J.L., and Schaechter, M. (1990). Physiology of the bacterial cell: a molecular approach (Springer).
6. Koblitz, J., Will, S.E., Riemer, S.A., Ulas, T., Neumann-Schaal, M., and Schomburg, D. (2021). The metano modeling toolbox MMTB: an intuitive, web-based toolbox introduced by two use cases. Metabolites *11*, 113. 10.3390/metabo11020113.
7. Price, M.N., Zane, G.M., Kuehl, J.V., Melnyk, R.A., Wall, J.D., Deutschbauer, A.M., and Arkin, A.P. (2018). Filling gaps in bacterial amino acid biosynthesis pathways with high-throughput genetics. PLOS Genetics *14*, e1007147. 10.1371/journal.pgen.1007147.
8. Barra, L., Fontenelle, C., Ermel, G., Trautwetter, A., Walker, G.C., and Blanco, C. (2006). Interrelations between glycine betaine catabolism and methionine biosynthesis in *Sinorhizobium meliloti* strain 102F34. Journal of Bacteriology *188*, 7195–7204. 10.1128/JB.00208-06.
9. Despotović, D., Aharon, E., Trofimyuk, O., Dubovetskyi, A., Cherukuri, K.P., Ashani, Y., Eliason, O., Sperfeld, M., Leader, H., Castelli, A., et al. (2022). Utilization of diverse organophosphorus pollutants by marine bacteria. Proceedings of the National Academy of Sciences *119*, e2203604119. 10.1073/pnas.2203604119.
10. Beiralas, R., Ozer, N., and Segev, E. (2023). Abundant Sulfitobacter marine bacteria protect Emiliania huxleyi algae from pathogenic bacteria. ISME COMMUN. *3*, 1–10. 10.1038/s43705-023-00311-y.
11. Kehe, J., Ortiz, A., Kulesa, A., Gore, J., Blainey, P.C., and Friedman, J. (2021). Positive interactions are common among culturable bacteria. Science Advances *7*, eabi7159. 10.1126/sciadv.abi7159.
12. Gebser, B., and Pohnert, G. (2013). Synchronized regulation of different zwitterionic metabolites in the osmoadaption of phytoplankton. Marine Drugs *11*, 2168–2182. 10.3390/md11062168.
13. Sperfeld, M., Yahalomi, D., and Segev, E. (2022). Resolving the microalgal gene landscape at the strain level: a novel hybrid transcriptome of *Emiliania huxleyi* CCMP3266. Applied and Environmental Microbiology *88*, e01418-21. 10.1128/AEM.01418-21.
14. Thole, S., Kalhoefer, D., Voget, S., Berger, M., Engelhardt, T., Liesegang, H., Wollherr, A., Kjelleberg, S., Daniel, R., Simon, M., et al. (2012). *Phaeobacter gallaeciensis* genomes from globally opposite locations reveal high similarity of adaptation to surface life. ISME J *6*, 2229–2244. 10.1038/ismej.2012.62.
15. O'Leary, N.A., Wright, M.W., Brister, J.R., Ciufo, S., Haddad, D., McVeigh, R., Rajput, B., Robbertse, B., Smith-White, B., Ako-Adjei, D., et al. (2016). Reference sequence (RefSeq) database at NCBI: current status, taxonomic expansion, and functional annotation. Nucleic Acids Research *44*, D733–D745. 10.1093/nar/gkv1189.
16. Kanehisa, M., and Goto, S. (2000). KEGG: Kyoto Encyclopedia of Genes and Genomes. Nucleic Acids Research *28*, 27–30. 10.1093/nar/28.1.27.
17. Karp, P.D., Billington, R., Caspi, R., Fulcher, C.A., Latendresse, M., Kothari, A., Keseler, I.M., Krummenacker, M., Midford, P.E., Ong, Q., et al. (2019). The BioCyc collection of microbial genomes and metabolic pathways. Briefings in Bioinformatics *20*, 1085–1093. 10.1093/bib/bbx085.
18. Kovach, M.E., Elzer, P.H., Steven Hill, D., Robertson, G.T., Farris, M.A., Roop, R.M., and Peterson, K.M. (1995). Four new derivatives of the broad-host-range cloning vector pBBR1MCS, carrying different antibiotic-resistance cassettes. Gene *166*, 175–176. 10.1016/0378-1119(95)00584-1.
19. Aronesty, E. (2013). Comparison of sequencing utility programs. The Open Bioinformatics Journal *7*. 10.2174/1875036201307010001.
20. Martin, M. (2011). Cutadapt removes adapter sequences from high-throughput sequencing reads. EMBnet.journal *17*, 10–12. 10.14806/ej.17.1.200.
21. Dobin, A., Davis, C.A., Schlesinger, F., Drenkow, J., Zaleski, C., Jha, S., Batut, P., Chaisson, M., and Gingeras, T.R. (2013). STAR: ultrafast universal RNA-seq aligner. Bioinformatics *29*, 15–21. 10.1093/bioinformatics/bts635.
22. Liao, Y., Smyth, G.K., and Shi, W. (2014). featureCounts: An efficient general purpose program for assigning sequence reads to genomic features. Bioinformatics *30*, 923–930. 10.1093/bioinformatics/btt656.

23. Love, M.I., Huber, W., and Anders, S. (2014). Moderated estimation of fold change and dispersion for RNA-seq data with DESeq2. Genome Biology *15*, 550. 10.1186/s13059-014-0550-8.

24. R Core Team (2022). R: A Language and Environment for Statistical Computing. R Foundation for Statistical Computing, Vienna, Austria. URL https://www.R-project.org/.

25. Wickham, H. (2016). ggplot2: Elegant Graphics for Data Analysis 2nd ed. 2016. (Springer International Publishing : Imprint: Springer) 10.1007/978-3-319-24277-4.

26. Sprouffske, K., and Wagner, A. (2016). Growthcurver: an R package for obtaining interpretable metrics from microbial growth curves. BMC Bioinformatics *17*, 172. 10.1186/s12859-016-1016-7.

27. Hothorn, T., Bretz, F., and Westfall, P. (2008). Simultaneous inference in general parametric models. Biometrical Journal *50*, 346–363. 10.1002/bimj.200810425.

28. Zhu, A., Ibrahim, J.G., and Love, M.I. (2019). Heavy-tailed prior distributions for sequence count data: removing the noise and preserving large differences. Bioinformatics *35*, 2084–2092. 10.1093/bioinformatics/bty895.

29. The UniProt Consortium (2023). UniProt: the universal protein knowledgebase in 2023. Nucleic Acids Research 51, D523–D531. 10.1093/nar/gkac1052.

30. Paysan-Lafosse, T., Blum, M., Chuguransky, S., Grego, T., Pinto, B.L., Salazar, G.A., Bileschi, M.L., Bork, P., Bridge, A., Colwell, L., et al. (2023). InterPro in 2022. Nucleic Acids Research 51, D418–D427. 10.1093/nar/gkac993.

31. Altschul, S.F., Gish, W., Miller, W., Myers, E.W., and Lipman, D.J. (1990). Basic local alignment search tool. Journal of Molecular Biology 215, 403–410. 10.1016/S0022-2836(05)80360-2.
