# Supplementary material for: Algal methylated compounds shorten the lag phase of Phaeobacter inhibens bacteria
Source: Nat Microbiol. 2024 Jul 5;9(8):2006–21. doi: 10.1038/s41564-024-01742-6 (PMC11306105; doi:10.1038/s41564-024-01742-6)
Supplement: Supplementary file 2 — Reporting Summary [file 41564_2024_1742_MOESM2_ESM.pdf]

nature portfolio

Corresponding author(s): Einat Segev

Last updated by author(s): Apr 28, 2024

Reporting Summary

Nature Portfolio wishes to improve the reproducibility of the work that we publish. This form provides structure for consistency and transparency in reporting. For further information on Nature Portfolio policies, see our Editorial Policies and the Editorial Policy Checklist.

Statistics

For all statistical analyses, confirm that the following items are present in the figure legend, table legend, main text, or Methods section.

n/a	Confirmed	
☐	☒	The exact sample size (*n*) for each experimental group/condition, given as a discrete number and unit of measurement
☐	☒	A statement on whether measurements were taken from distinct samples or whether the same sample was measured repeatedly
☐	☒	The statistical test(s) used AND whether they are one- or two-sided<br>*Only common tests should be described solely by name; describe more complex techniques in the Methods section.*
☐	☒	A description of all covariates tested
☐	☒	A description of any assumptions or corrections, such as tests of normality and adjustment for multiple comparisons
☐	☒	A full description of the statistical parameters including central tendency (e.g. means) or other basic estimates (e.g. regression coefficient) AND variation (e.g. standard deviation) or associated estimates of uncertainty (e.g. confidence intervals)
☐	☒	For null hypothesis testing, the test statistic (e.g. *F*, *t*, *r*) with confidence intervals, effect sizes, degrees of freedom and *P* value noted<br>*Give P values as exact values whenever suitable.*
☒	☐	For Bayesian analysis, information on the choice of priors and Markov chain Monte Carlo settings
☒	☐	For hierarchical and complex designs, identification of the appropriate level for tests and full reporting of outcomes
☒	☐	Estimates of effect sizes (e.g. Cohen's *d*, Pearson's *r*), indicating how they were calculated

Our web collection on statistics for biologists contains articles on many of the points above.

Software and code

Policy information about availability of computer code

Data collection	A description of all commercial or open source tools and/or code used to collect the data, including settings, version numbers and citations, is reported in the Key Resources Table and/or the Method section of the manuscript. No custom code was generated.
Data analysis	A description of all commercial or open source tools and/or code used to analyze the data, including settings, version numbers and citations, is reported in the Key Resources Table and/or the Method section of the manuscript. No custom code was generated.<br>Briefly:<br>RNA-sequencing data analysis: read mapping was conducted with STAR v2.7.5c, reads that mapped to E. huxleyi features were counted using featureCounts from the subread package v2.0.0, to analyze sample variability, bacterial non-rRNA feature count results were normalized for sequencing depth and RNA composition using the DESeq2's median of ratios method, followed by a regularized log transformation and principle component analysis v1.36.0 with R version 4.2.0.<br>Growth data analysis: Results were plotted in R (v4.2.0) using ggplot2 (v3.4.1), Doubling times and lag times were calculated for each replicate using the Growthcurver package (v0.3.1).<br>LC-MS analysis: Data processing was conducetd using the Compound Discoverer software v3.3.<br>Bioinformatic analysis of methyl group-related metabolism in algal- associated and plant-associated bacteria: The analysis was conducted using OrthoFinder, which utilizes MCL and FastME algorithms with default settings.

For manuscripts utilizing custom algorithms or software that are central to the research but not yet described in published literature, software must be made available to editors and reviewers. We strongly encourage code deposition in a community repository (e.g. GitHub). See the Nature Portfolio guidelines for submitting code & software for further information.

Data

Policy information about availability of data

All manuscripts must include a data availability statement. This statement should provide the following information, where applicable:

- Accession codes, unique identifiers, or web links for publicly available datasets
- A description of any restrictions on data availability
- For clinical datasets or third party data, please ensure that the statement adheres to our policy

All data are available in the main text or the supplementary materials. Further information and requests for resources and reagents should be directed to and will be fulfilled by the lead contact, Einat Segev. Plasmids generated in this study, as well as bacteria that are not deposited in public strain collections, will be made available on reasonable request.
RNA-sequencing data have been deposited in the NCBI Sequence Read Archive (SRA) and are publicly available under accession PRJNA976961 (algal-bacterial RNA-seq) and PRJNA977030 (bacterial lag phase RNA-seq).
Data S1-S4 have been deposited in Zenodo and can be accessed here https://zenodo.org/records/10980548.
Bacterial methyl metabolism functional annotations were generated using NCBI RefSeq annotations (https://doi.org/10.1093/nar/gkv1189), partially curated GenBank submitter annotations (https://doi.org/10.1038/ismej.2012.62), KEGG annotations (https://doi.org/10.1093/nar/28.1.27), BioCyc (https://doi.org/10.1093/bib/bbx085) and the unifying UniProt Archive (UniParc https://doi.org/10.1093/nar/gkac1052 and InterPro database https://doi.org/10.1093/nar/gkac993).
Metabolomics raw data have been uploaded to the EMBL-EBI MetaboLights database with the identifier MTBLS9742 and can be accessed here https://www.ebi.ac.uk/metabolights/MTBLS9742. Data can also be obtained from the corresponding author Einat.Segev@weizmann.ac.il.

Research involving human participants, their data, or biological material

Policy information about studies with human participants or human data. See also policy information about sex, gender (identity/presentation), and sexual orientation and race, ethnicity and racism.

Reporting on sex and gender	not applicable
Reporting on race, ethnicity, or other socially relevant groupings	not applicable
Population characteristics	not applicable
Recruitment	not applicable
Ethics oversight	not applicable

Note that full information on the approval of the study protocol must also be provided in the manuscript.

Field-specific reporting

Please select the one below that is the best fit for your research. If you are not sure, read the appropriate sections before making your selection.

☒ Life sciences ☐ Behavioural & social sciences ☐ Ecological, evolutionary & environmental sciences

For a reference copy of the document with all sections, see nature.com/documents/nr-reporting-summary-flat.pdf

Life sciences study design

All studies must disclose on these points even when the disclosure is negative.

Sample size	No statistical method was used to predetermine sample size, samples included at least three replicates.
Data exclusions	No data was excluded.
Replication	All experiments were conducted using at least three replicates and all attempts of replication were successful.
Randomization	Samples were allocated randomly.
Blinding	The investigators were blinded to group allocation during data collection and/or analysis.

Reporting for specific materials, systems and methods

We require information from authors about some types of materials, experimental systems and methods used in many studies. Here, indicate whether each material, system or method listed is relevant to your study. If you are not sure if a list item applies to your research, read the appropriate section before selecting a response.

Materials & experimental systems

n/a	Involved in the study
☒	☐ Antibodies
☒	☐ Eukaryotic cell lines
☒	☐ Palaeontology and archaeology
☒	☐ Animals and other organisms
☒	☐ Clinical data
☒	☐ Dual use research of concern
☒	☐ Plants

Methods

n/a	Involved in the study
☒	☐ ChIP-seq
☒	☐ Flow cytometry
☒	☐ MRI-based neuroimaging
